# Supplementary figures and images for: Hsp47 promotes biogenesis of multi-subunit neuroreceptors in the endoplasmic reticulum (part 1 of 2)
Source: eLife. 2024 Jul 4;13:e84798. doi: 10.7554/eLife.84798 (PMC11257679; doi:10.7554/eLife.84798)

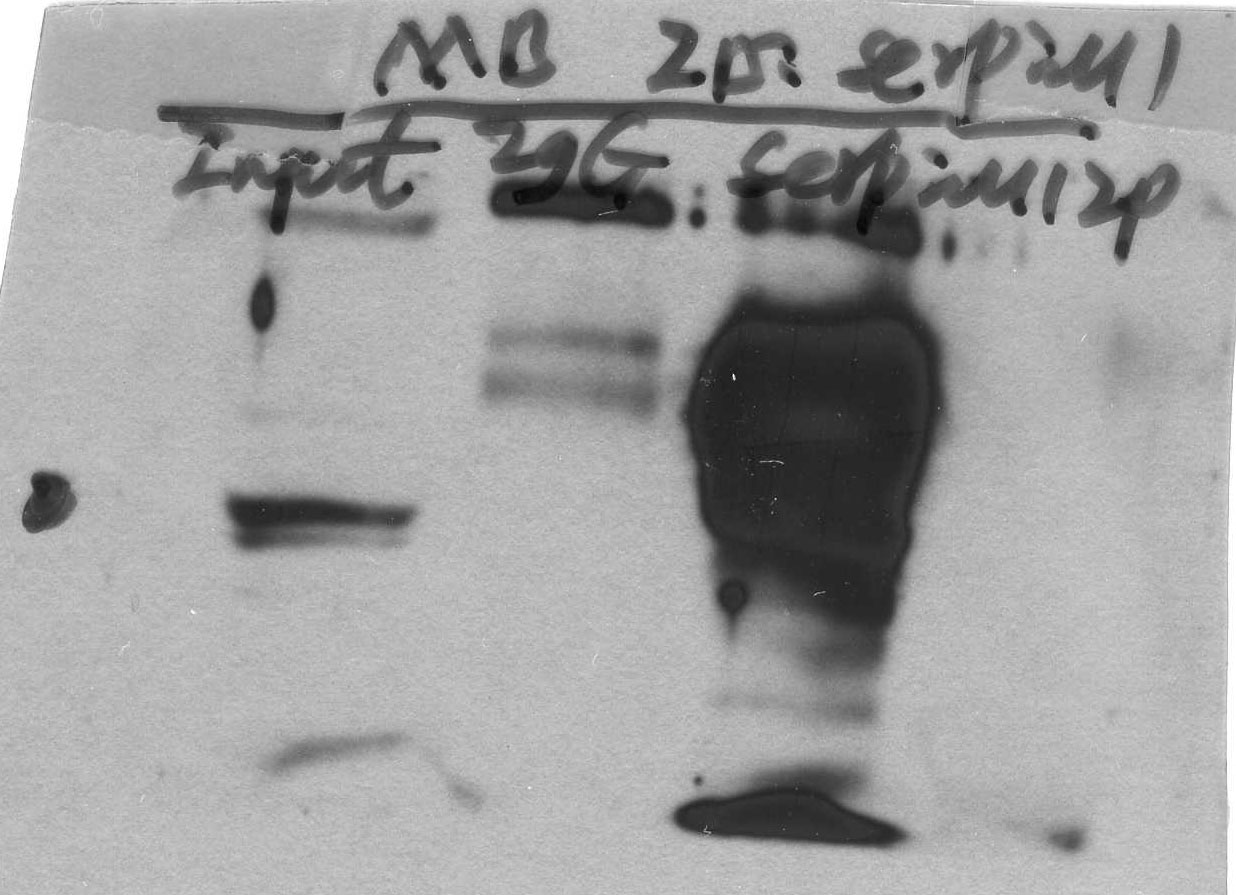

Supplement: Figure 1—source data 1. [file elife-84798-fig1-data1.zip › Figure 1-source data 1 /Figure 1-source data 1.jpg]

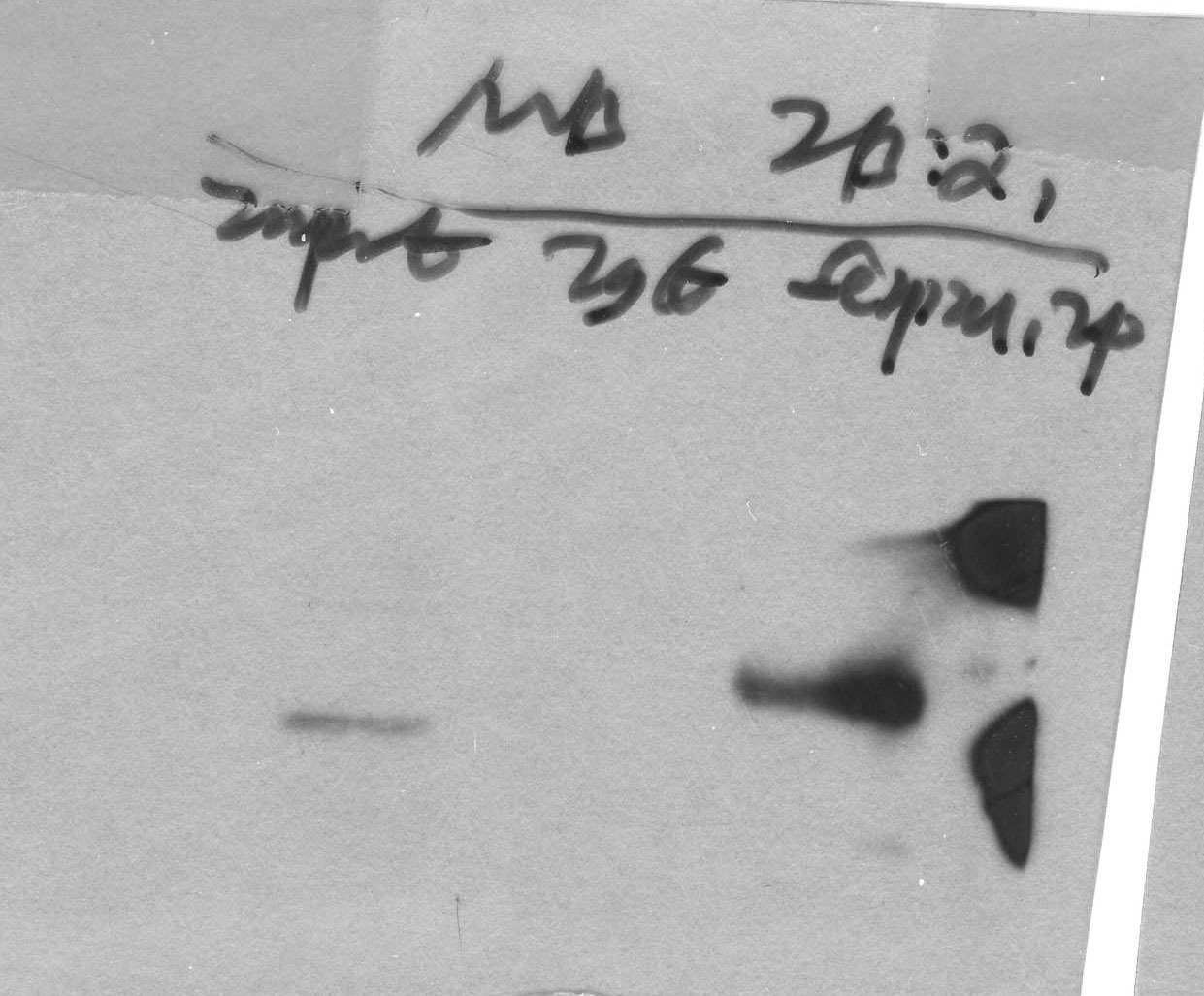

Supplement: Figure 1—source data 1. [file elife-84798-fig1-data1.zip › Figure 1-source data 2/Figure 1-source data 2.jpg]

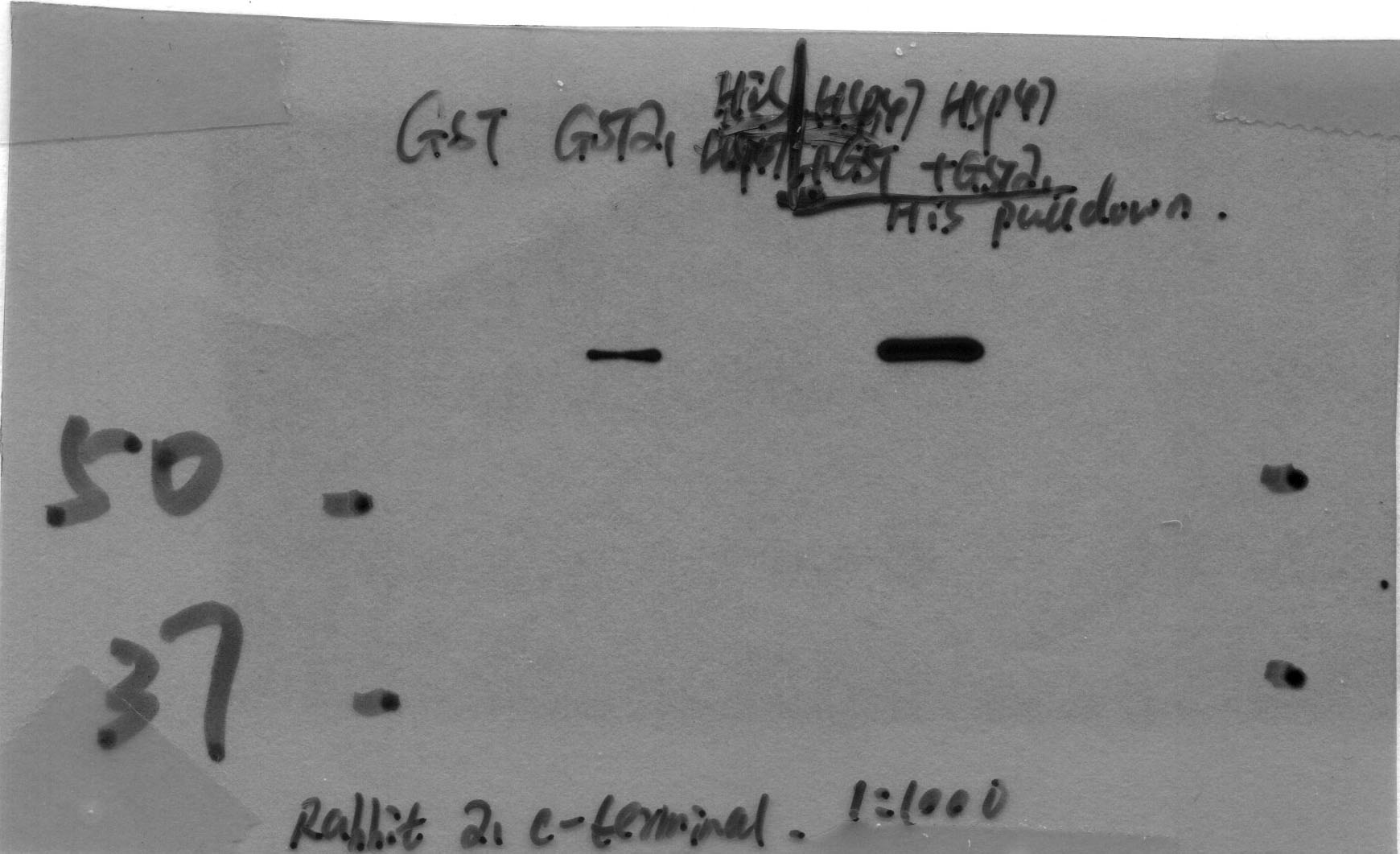

Supplement: Figure 1—source data 1. [file elife-84798-fig1-data1.zip › Figure 1-source data 3/Figure 1-source data 3.jpg]

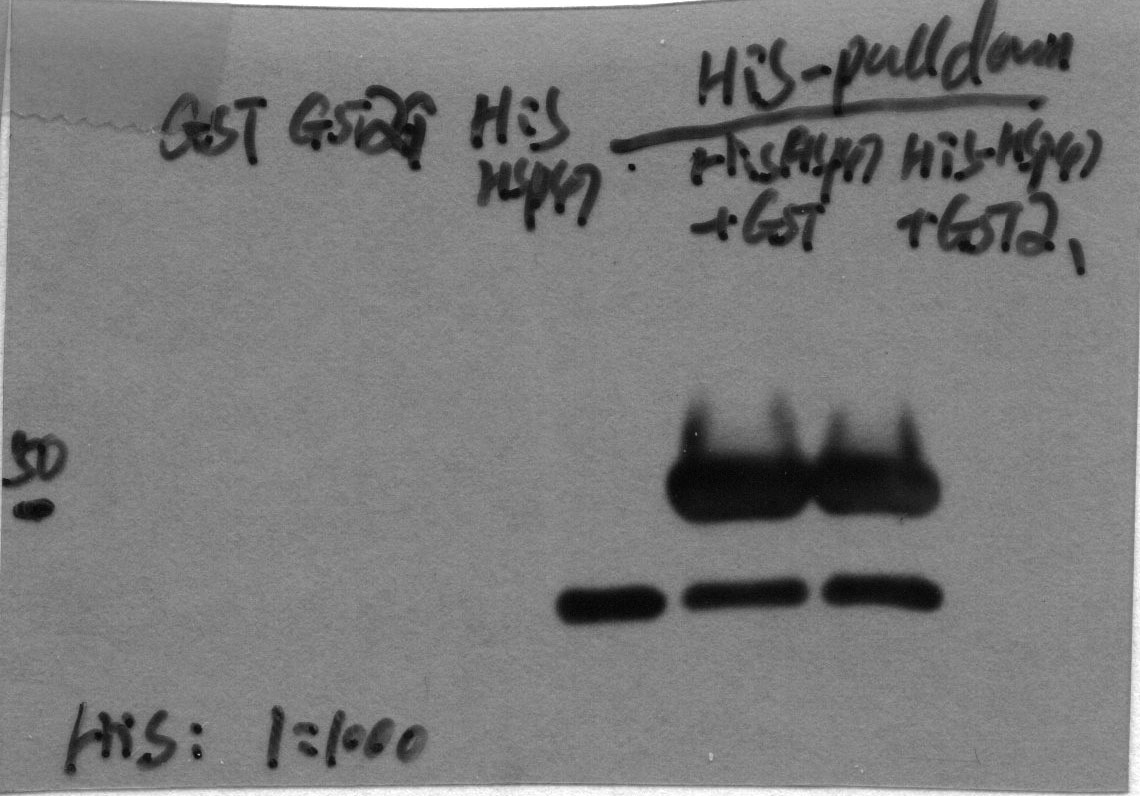

Supplement: Figure 1—source data 1. [file elife-84798-fig1-data1.zip › Figure 1-source data 4/Figure 1-source data 4.jpg]

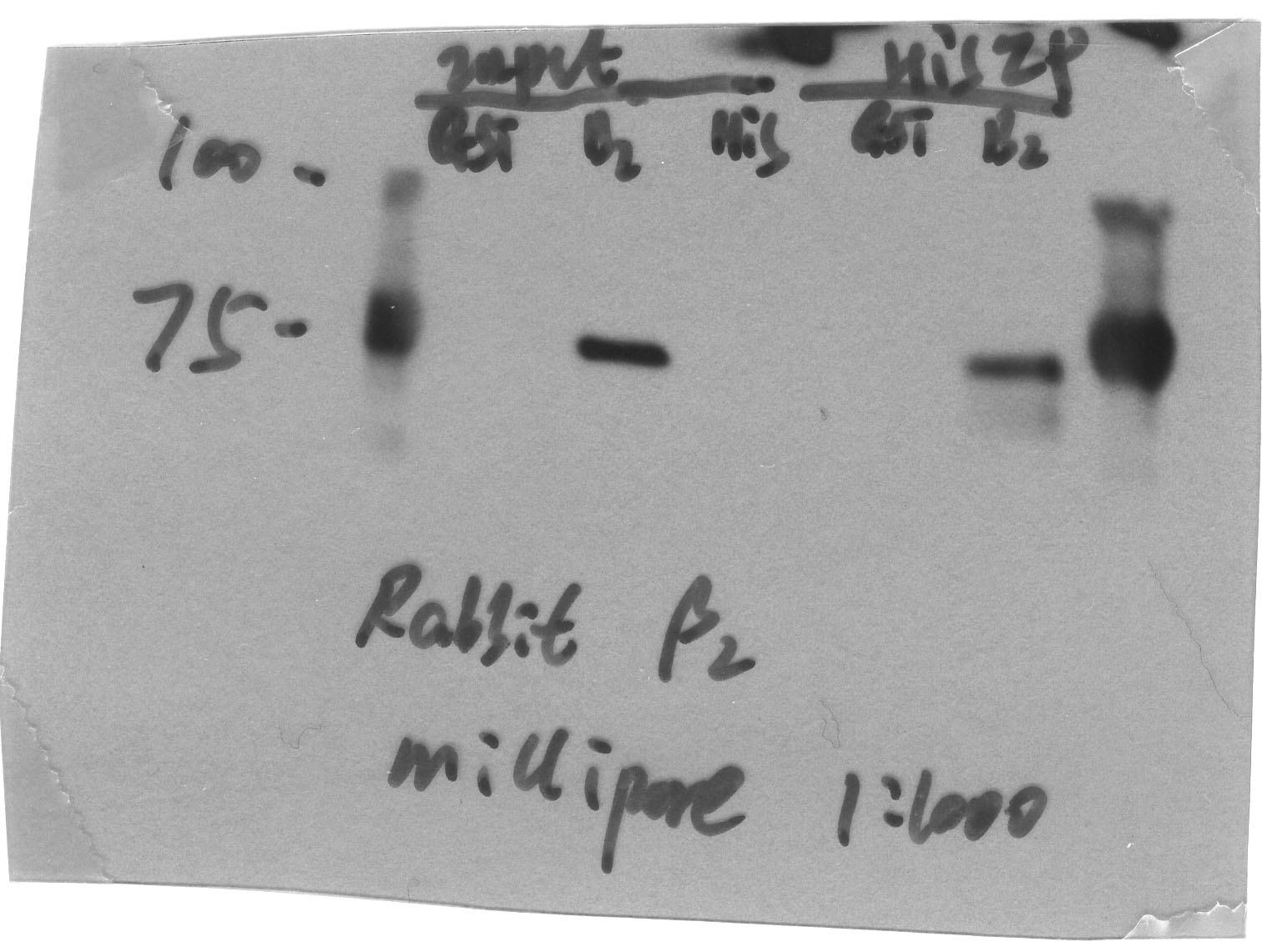

Supplement: Figure 1—source data 1. [file elife-84798-fig1-data1.zip › Figure 1-source data 5/Figure 1-source data 5.jpg]

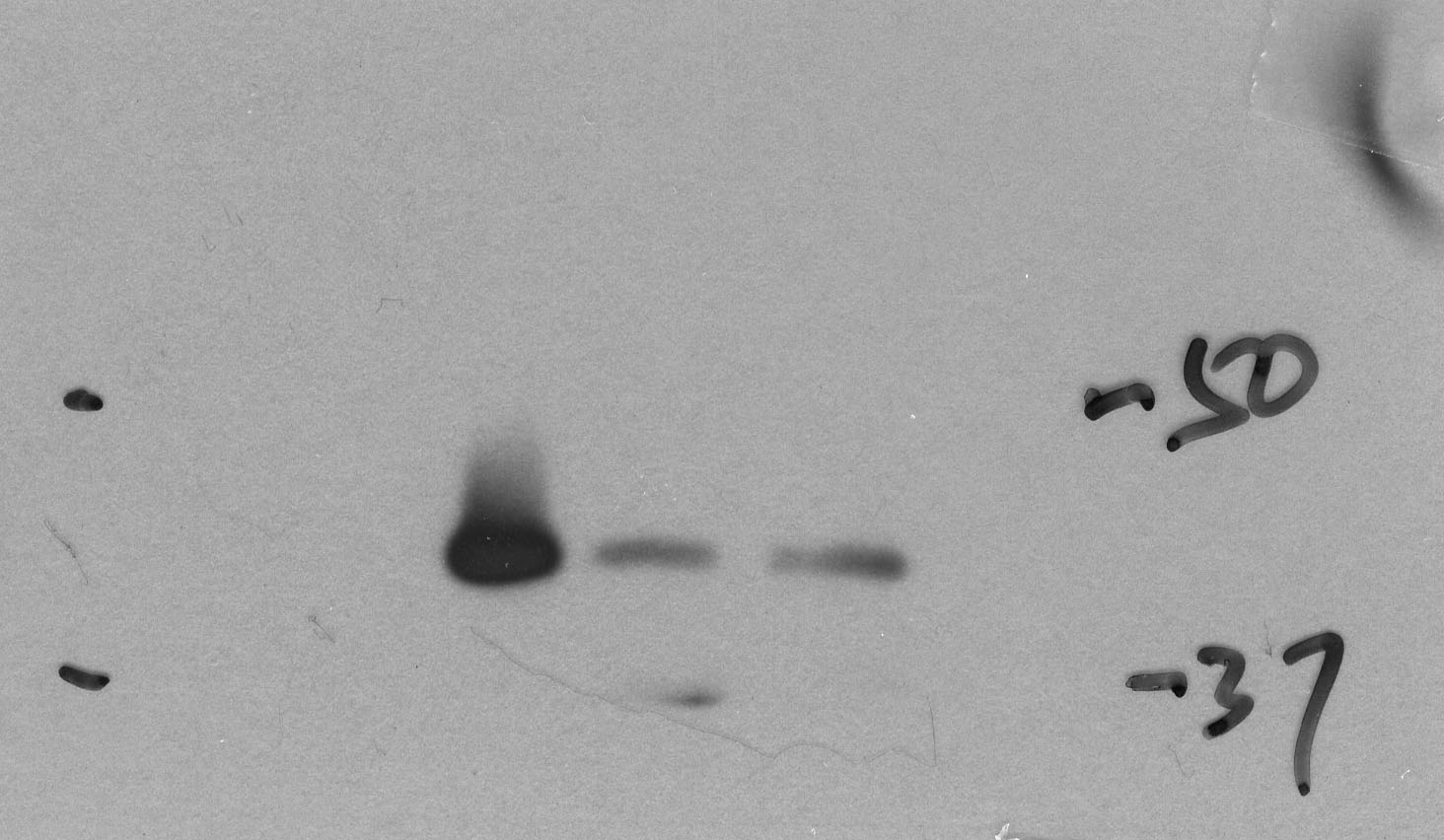

Supplement: Figure 1—source data 1. [file elife-84798-fig1-data1.zip › Figure 1-source data 6/Figure 1-source data 6.jpg]

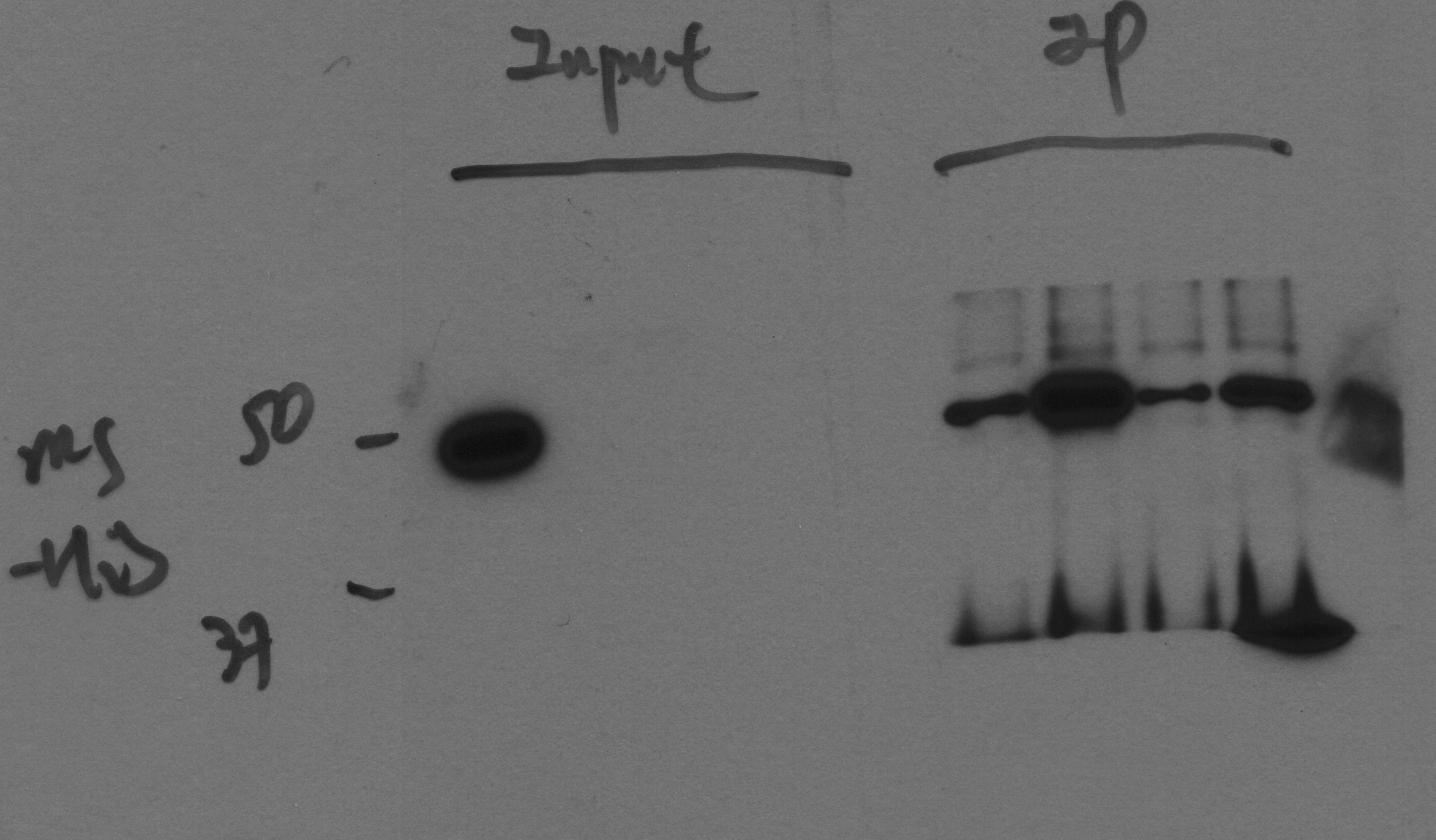

Supplement: Figure 1—figure supplement 2—source data 1. [file elife-84798-fig1-figsupp2-data1.zip › Figure 1-figure supplement 2-source data 1/Figure 1-figure supplement 2-source data 1.tif]

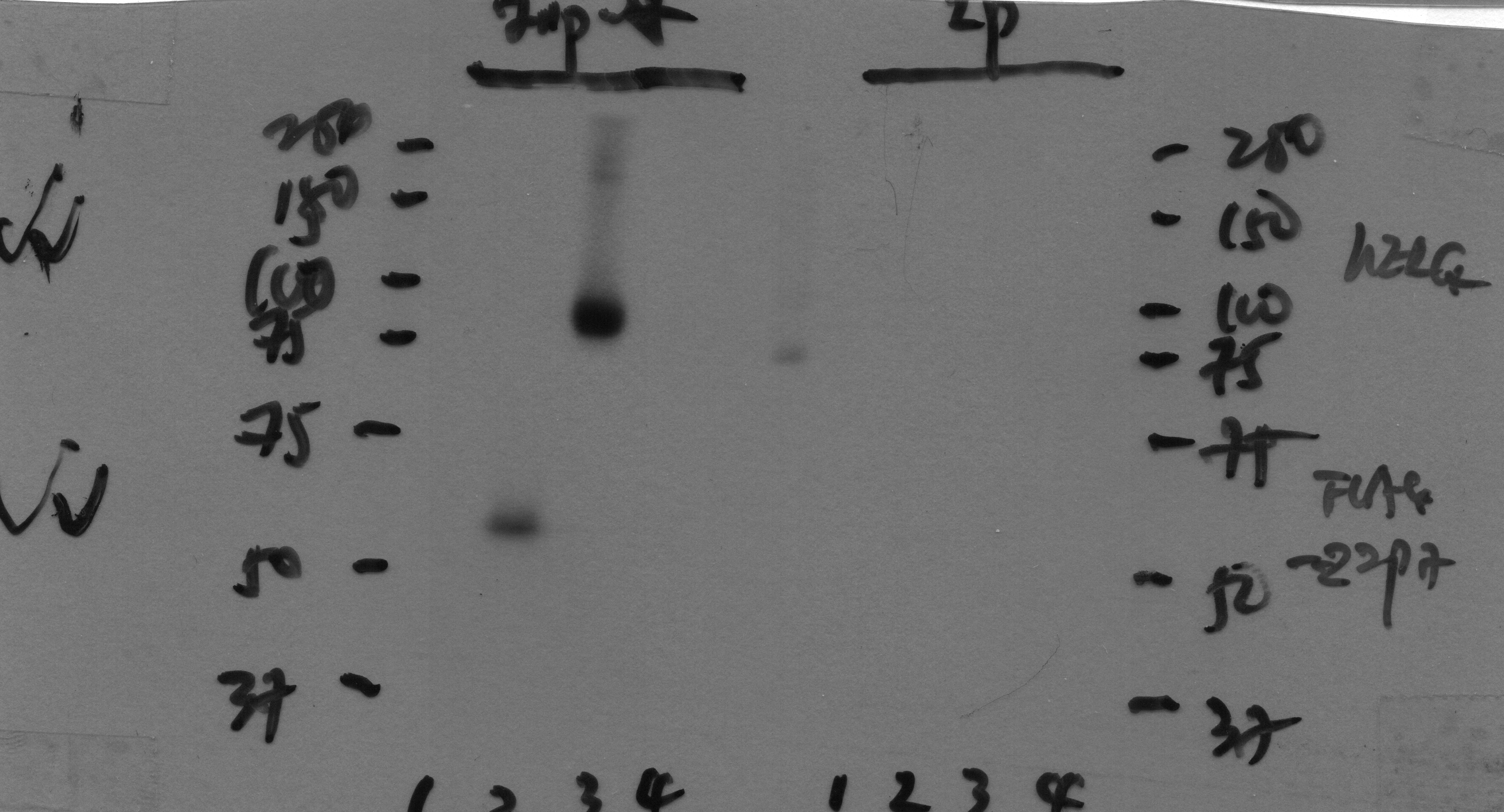

Supplement: Figure 1—figure supplement 2—source data 1. [file elife-84798-fig1-figsupp2-data1.zip › Figure 1-figure supplement 2-source data 2/Figure 1-figure supplement 2-source data 2.tif]

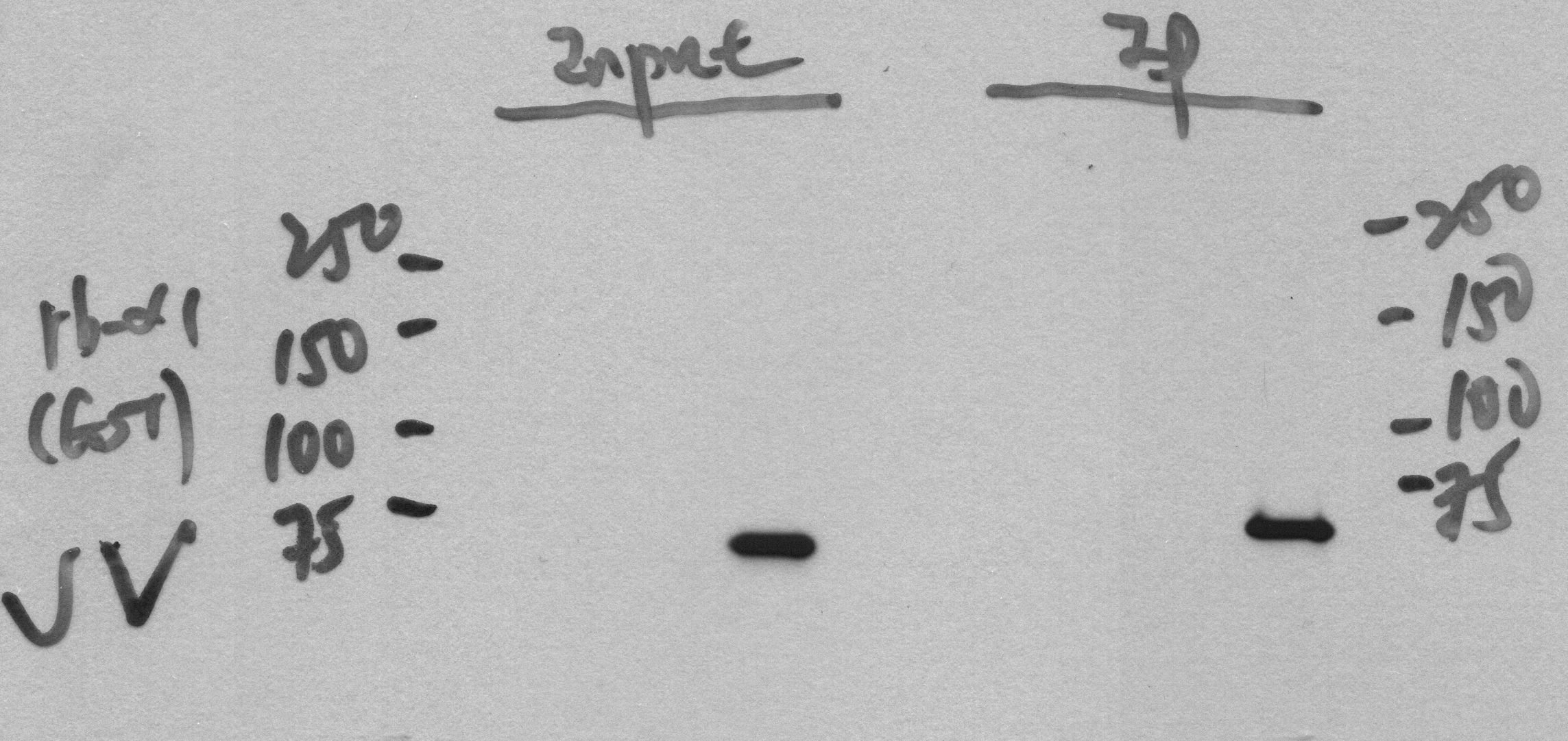

Supplement: Figure 1—figure supplement 2—source data 1. [file elife-84798-fig1-figsupp2-data1.zip › Figure 1-figure supplement 2-source data 3/Figure 1-figure supplement 2-source data 3.tif]

Figure 1—figure supplement 2A

Top row

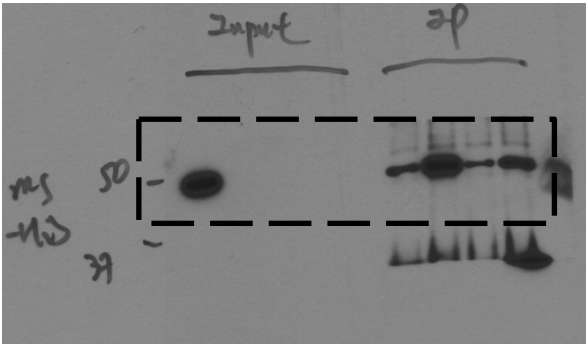

IB: His  
(Hsp47)

3rd row

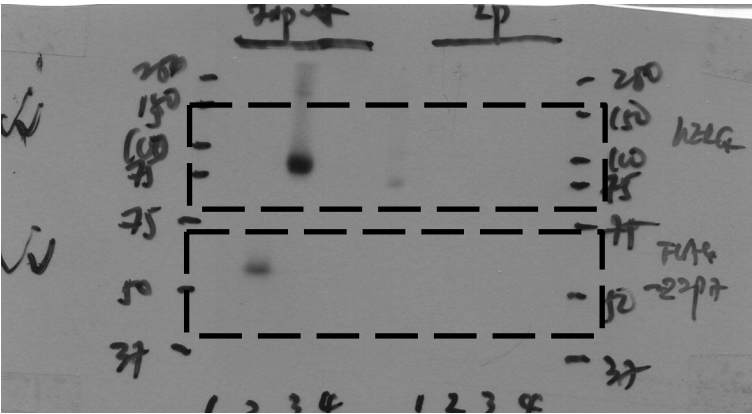

IB: hERG

2nd row

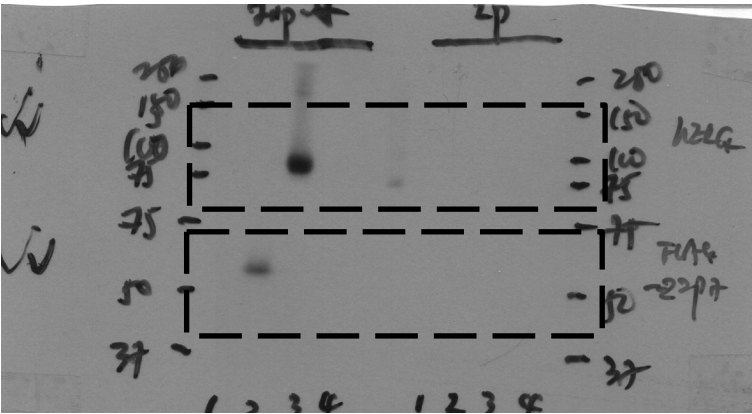

IB: FLAG  
(ZIP7)

Bottom row

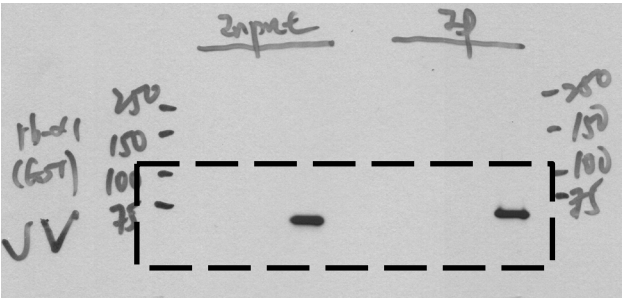

IB:  $\alpha 1$

Supplement: Figure 1—figure supplement 2—source data 2. [file elife-84798-fig1-figsupp2-data2.zip › Figure 1-figure supplement 2-source data 4/Figure 1-figure supplement 2-source data 4.pdf]

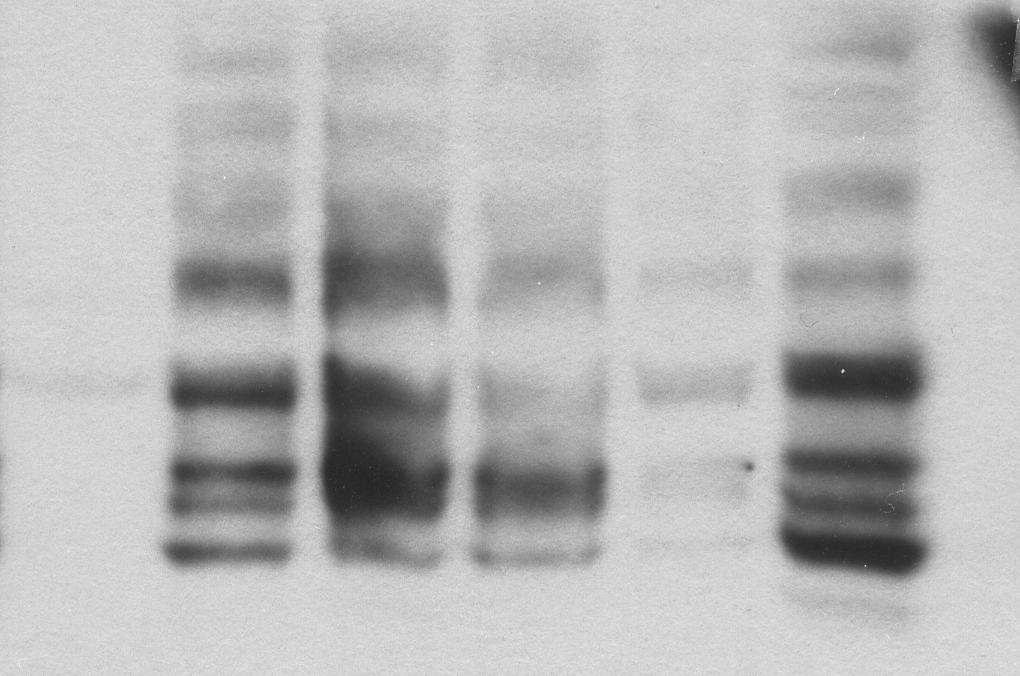

Supplement: Figure 2—figure supplement 1—source data 1. [file elife-84798-fig2-figsupp1-data1.zip › Figure 2-figure supplement 1-source data 1/Figure 2-figure supplement 1-source data 1.jpg]

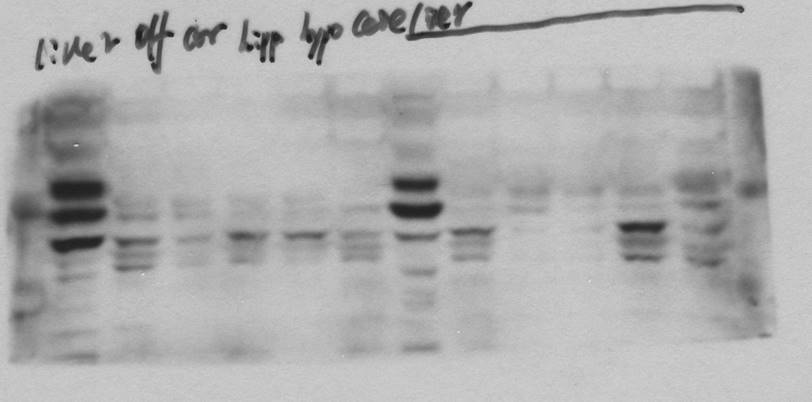

Supplement: Figure 2—figure supplement 1—source data 1. [file elife-84798-fig2-figsupp1-data1.zip › Figure 2-figure supplement 1-source data 2/Figure 2-figure supplement 1-source data 2.jpg]

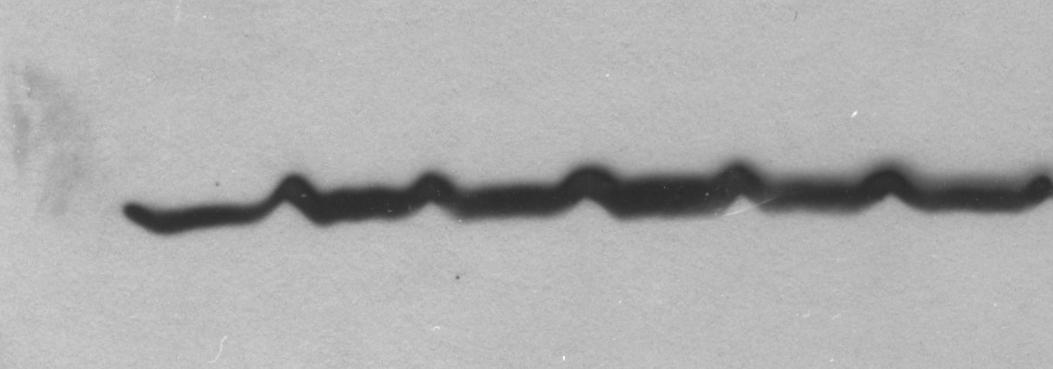

Supplement: Figure 2—figure supplement 1—source data 1. [file elife-84798-fig2-figsupp1-data1.zip › Figure 2-figure supplement 1-source data 3/Figure 2-figure supplement 1-source data 3.jpg]

Figure 2—figure supplement 1A

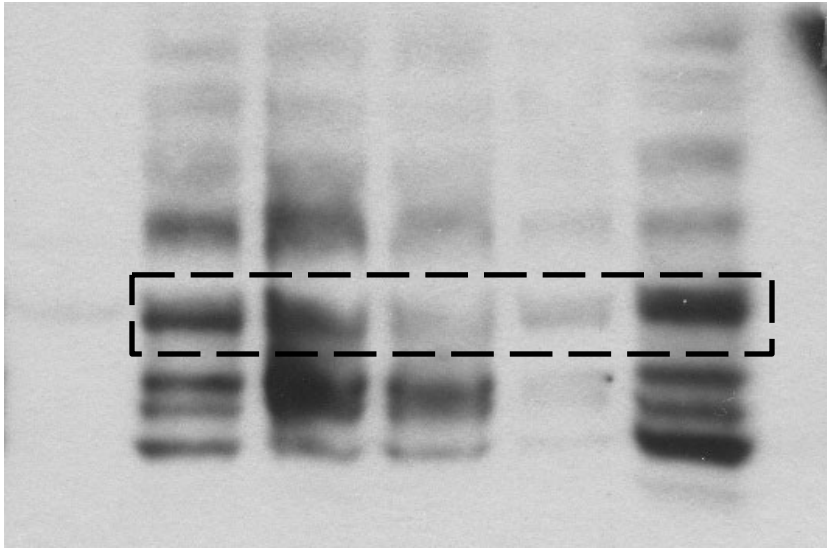

Top panel

IB:  $\beta$ 2/3

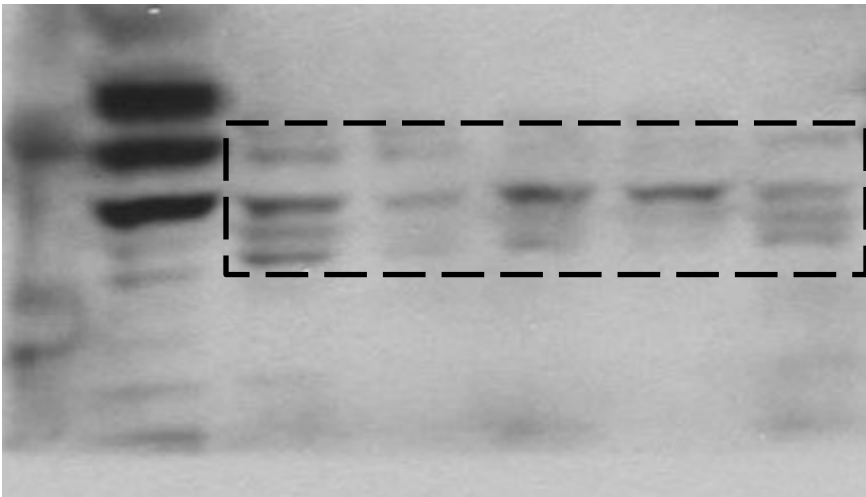

Middle panel

IB: Hsp47

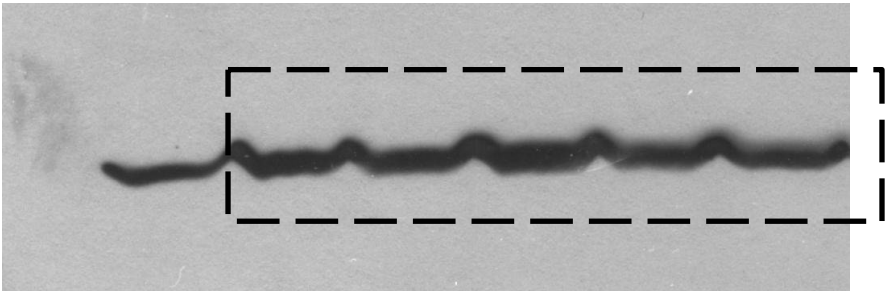

Bottom panel

IB:  $\beta$ -actin

Supplement: Figure 2—figure supplement 1—source data 2. [file elife-84798-fig2-figsupp1-data2.zip › Figure 2-figure supplement 1-source data 4/Figure 2-figure supplement 1-source data 4.pdf]

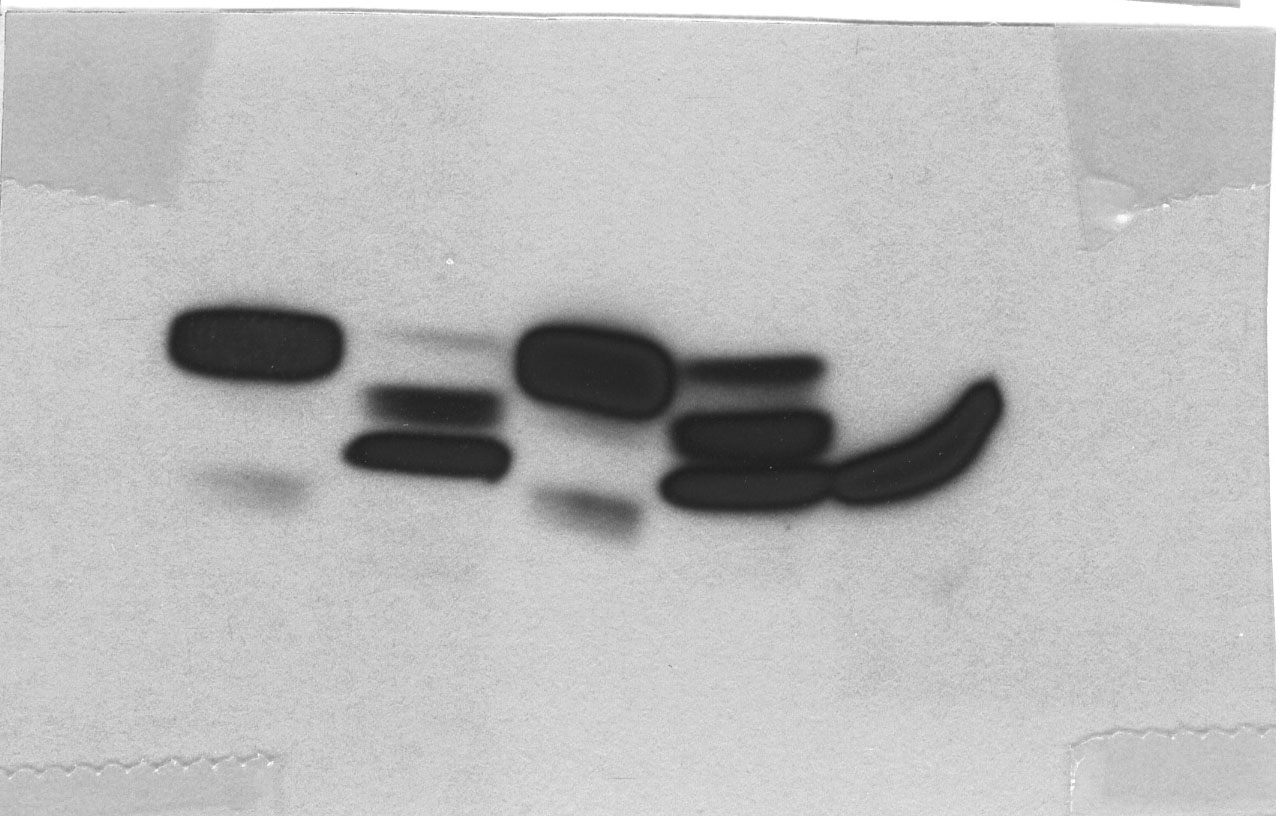

Supplement: Figure 3—source data 1. [file elife-84798-fig3-data1.zip › Figure 3-source data 1 /Figure 3-source data 1.jpg]

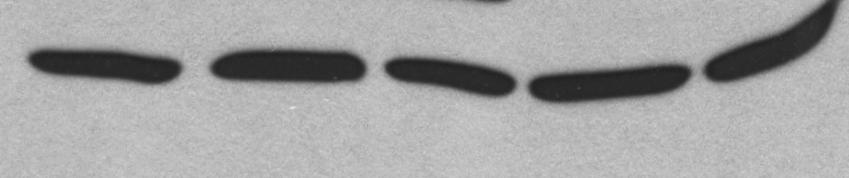

Supplement: Figure 3—source data 1. [file elife-84798-fig3-data1.zip › Figure 3-source data 2/Figure 3-source data 2.jpg]

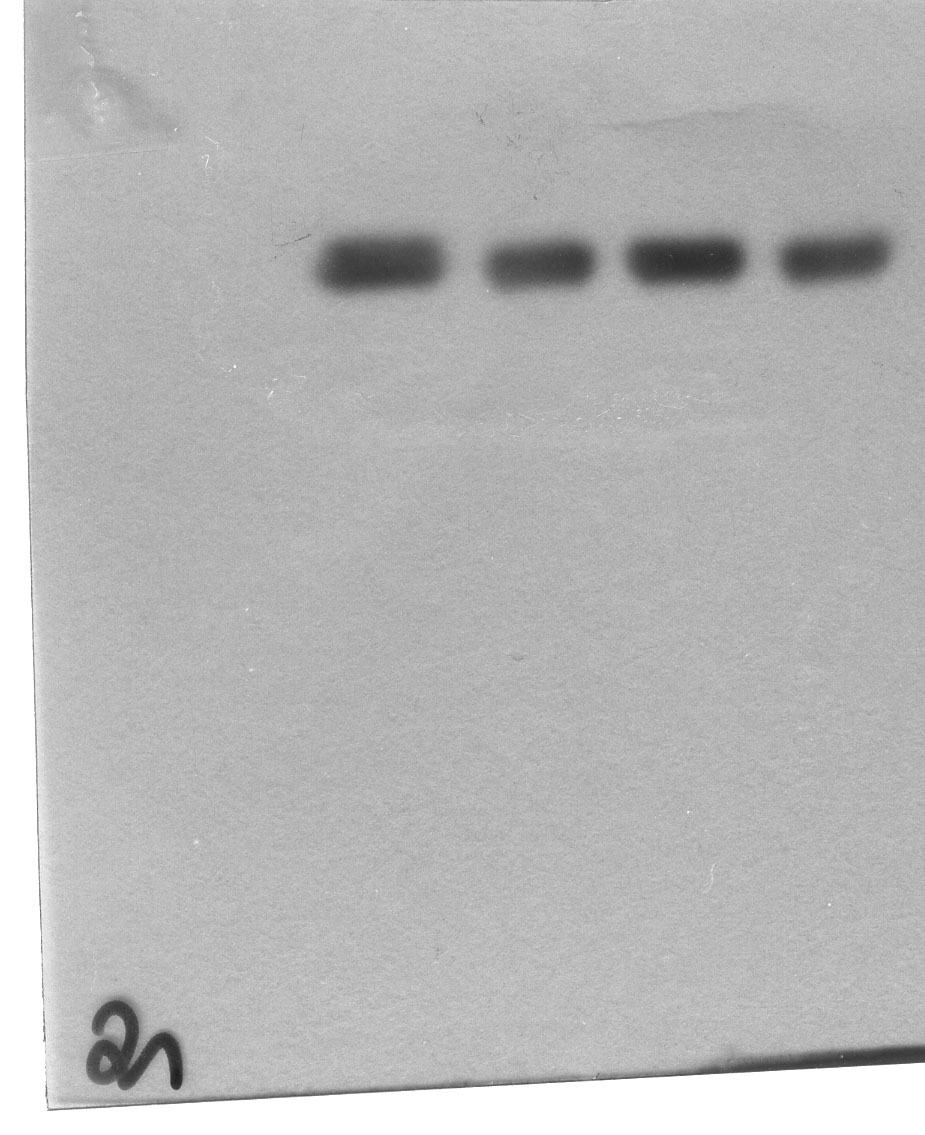

Supplement: Figure 3—source data 1. [file elife-84798-fig3-data1.zip › Figure 3-source data 3/Figure 3-source data 3.jpg]

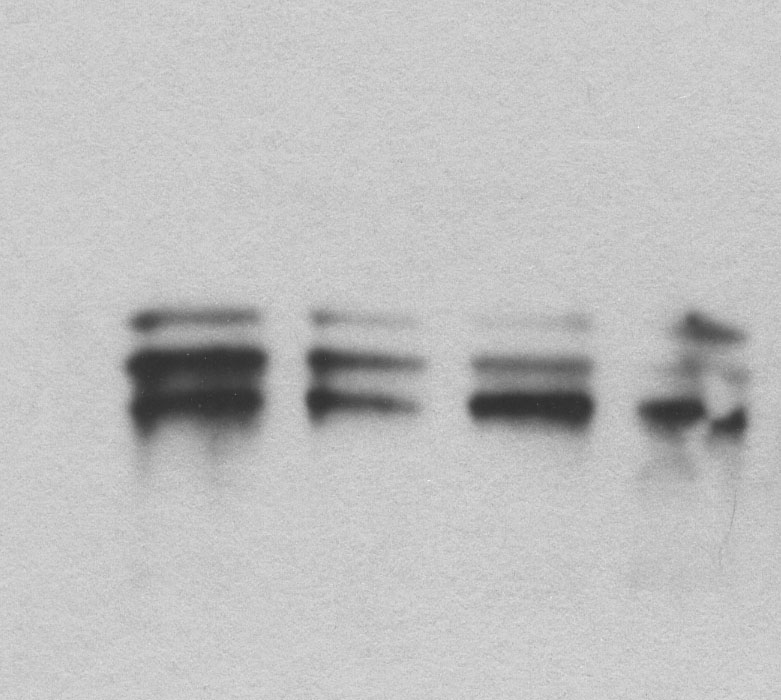

Supplement: Figure 3—source data 1. [file elife-84798-fig3-data1.zip › Figure 3-source data 4/Figure 3-source data 4.jpg]

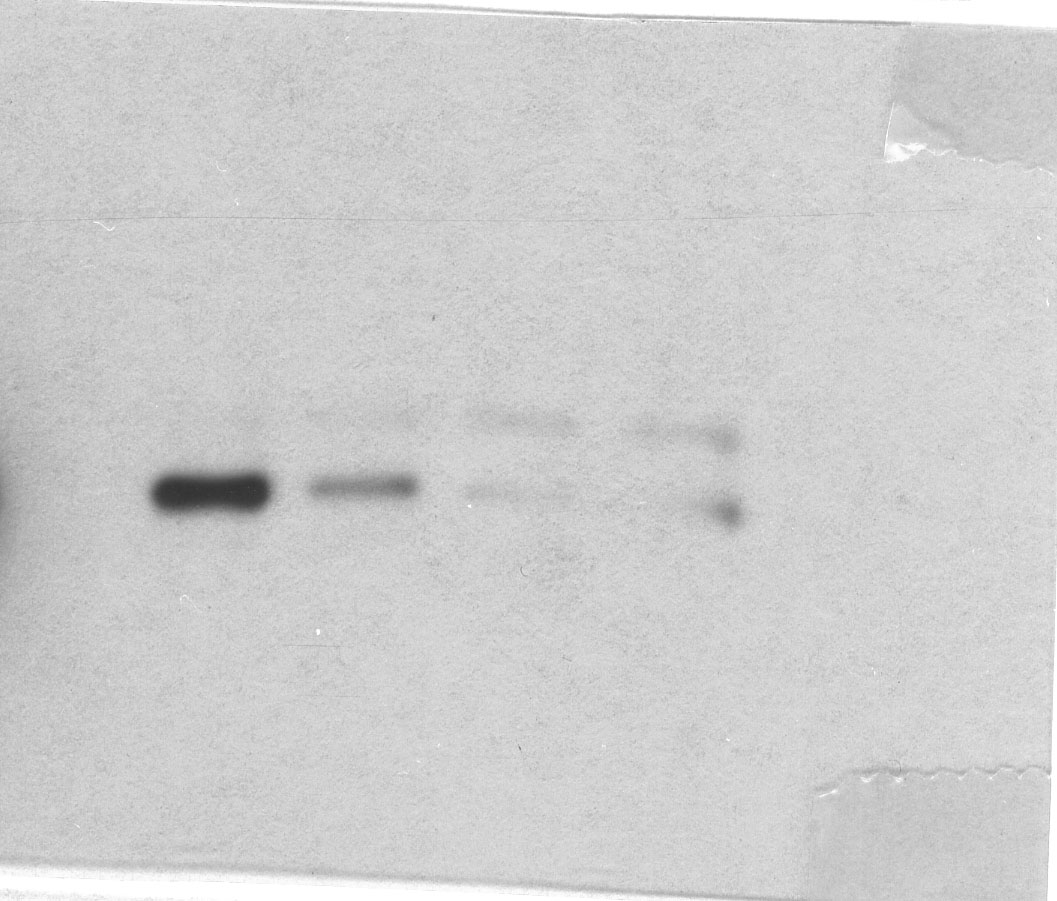

Supplement: Figure 3—source data 1. [file elife-84798-fig3-data1.zip › Figure 3-source data 5/Figure 3-source data 5.jpg]

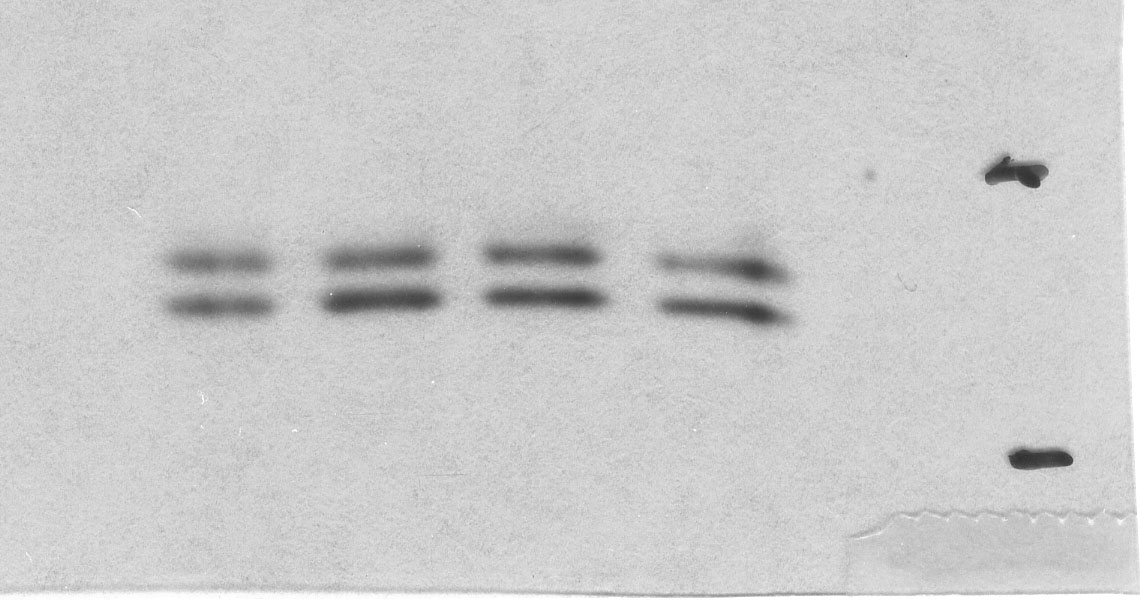

Supplement: Figure 3—source data 1. [file elife-84798-fig3-data1.zip › Figure 3-source data 6/Figure 3-source data 6.jpg]

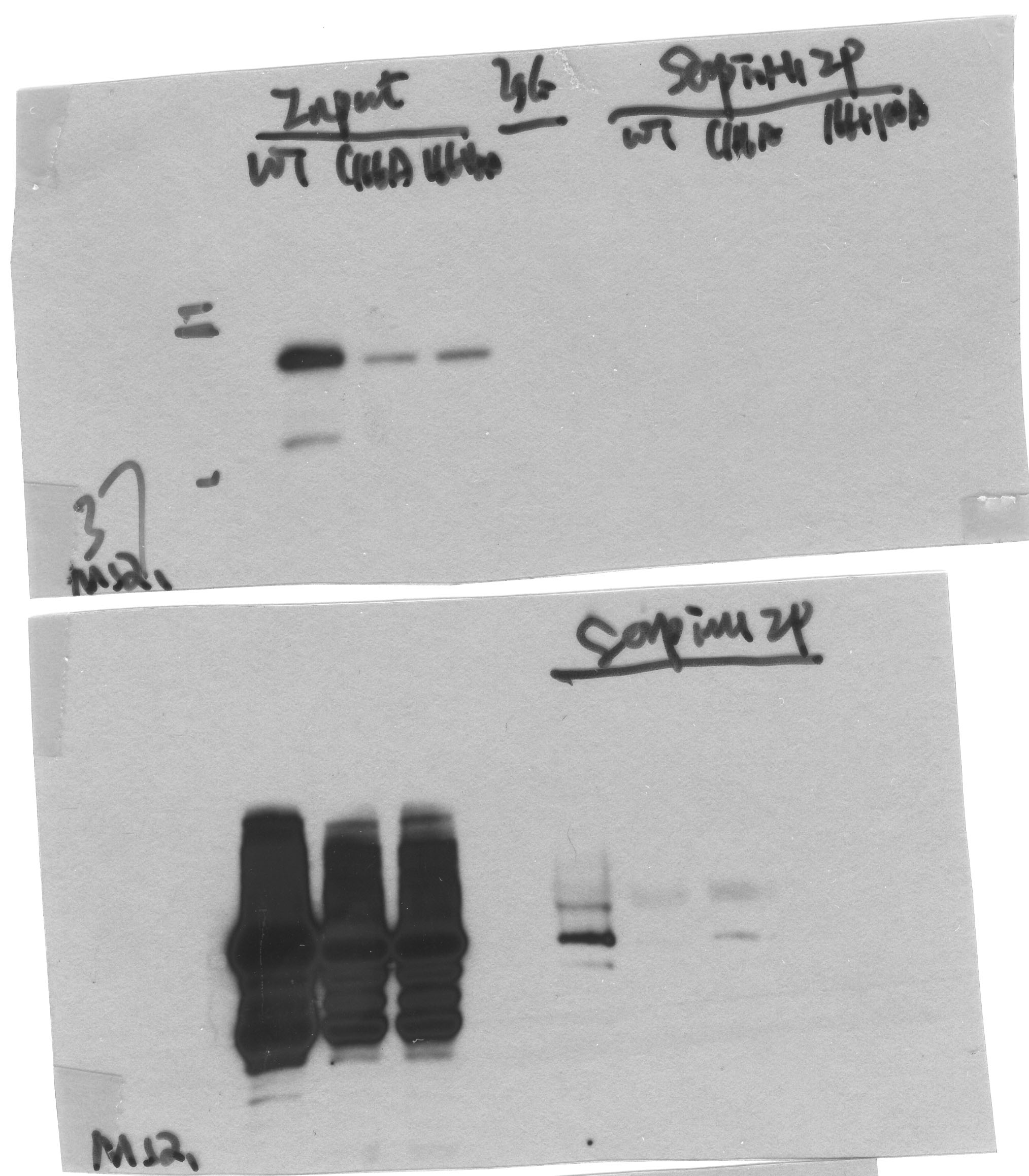

Supplement: Figure 3—source data 1. [file elife-84798-fig3-data1.zip › Figure 3-source data 7/Figure 3-source data 7.jpg]

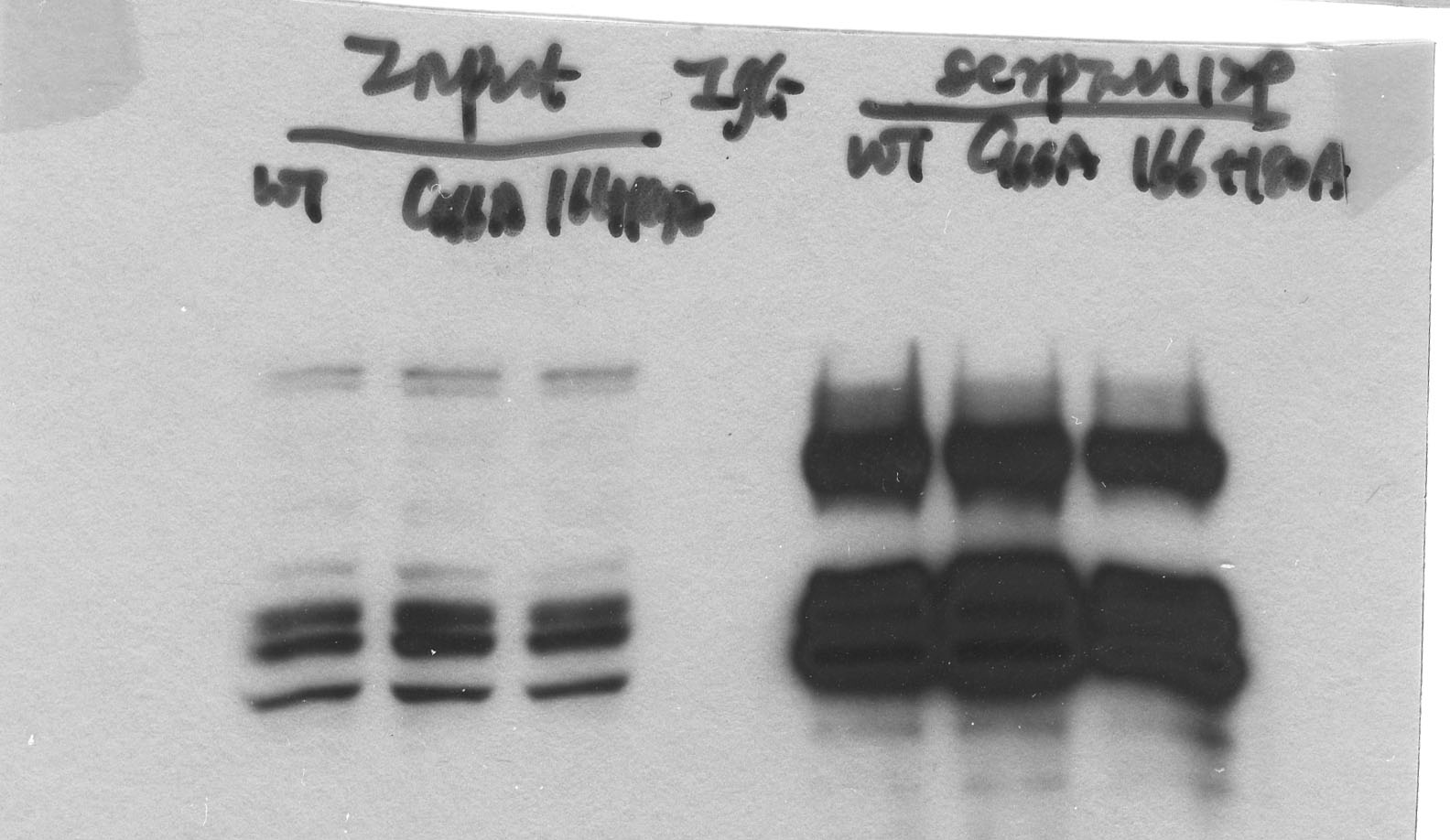

Supplement: Figure 3—source data 1. [file elife-84798-fig3-data1.zip › Figure 3-source data 8/Figure 3-source data 8.jpg]

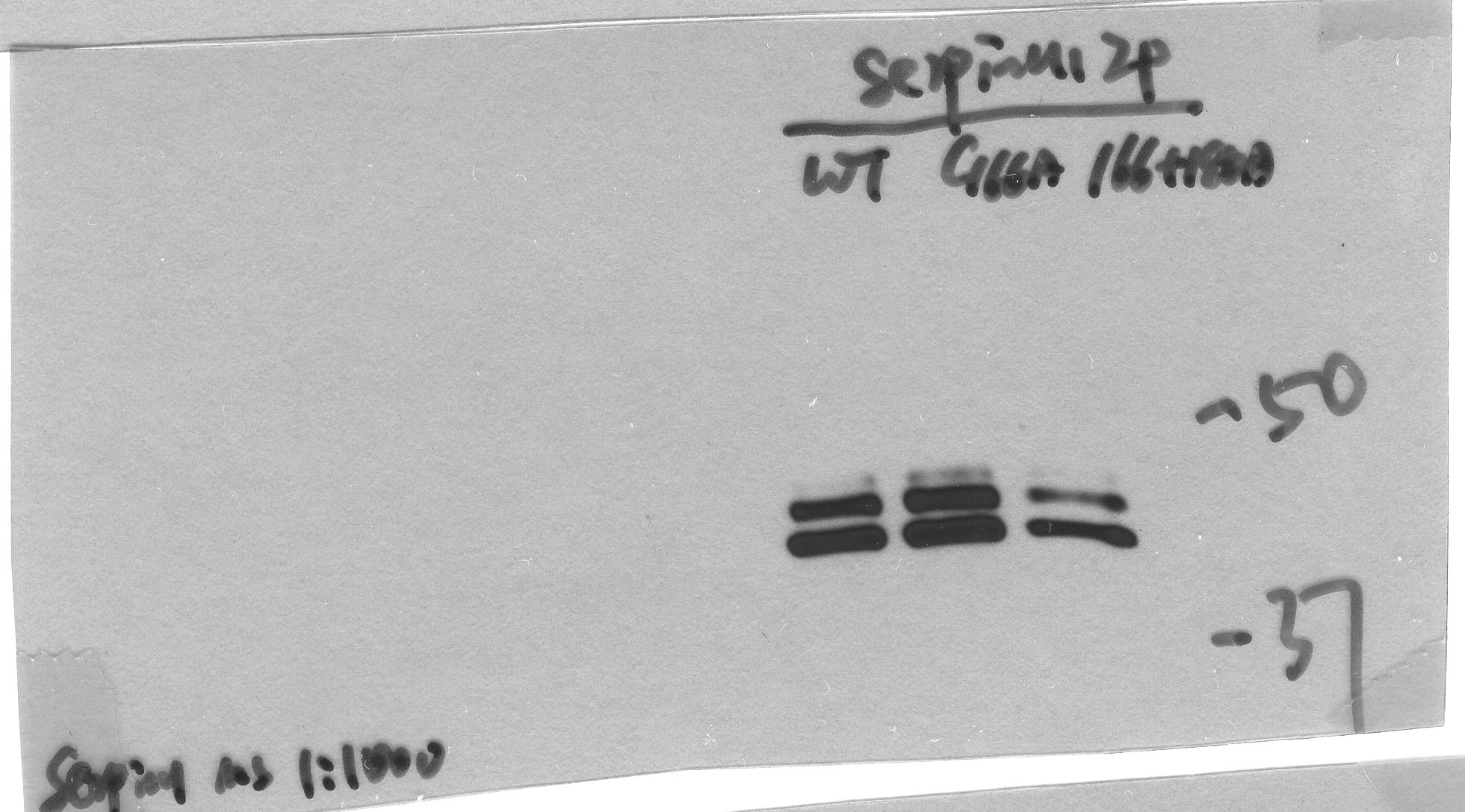

Supplement: Figure 3—source data 1. [file elife-84798-fig3-data1.zip › Figure 3-source data 9/Figure 3-source data 9.jpg]

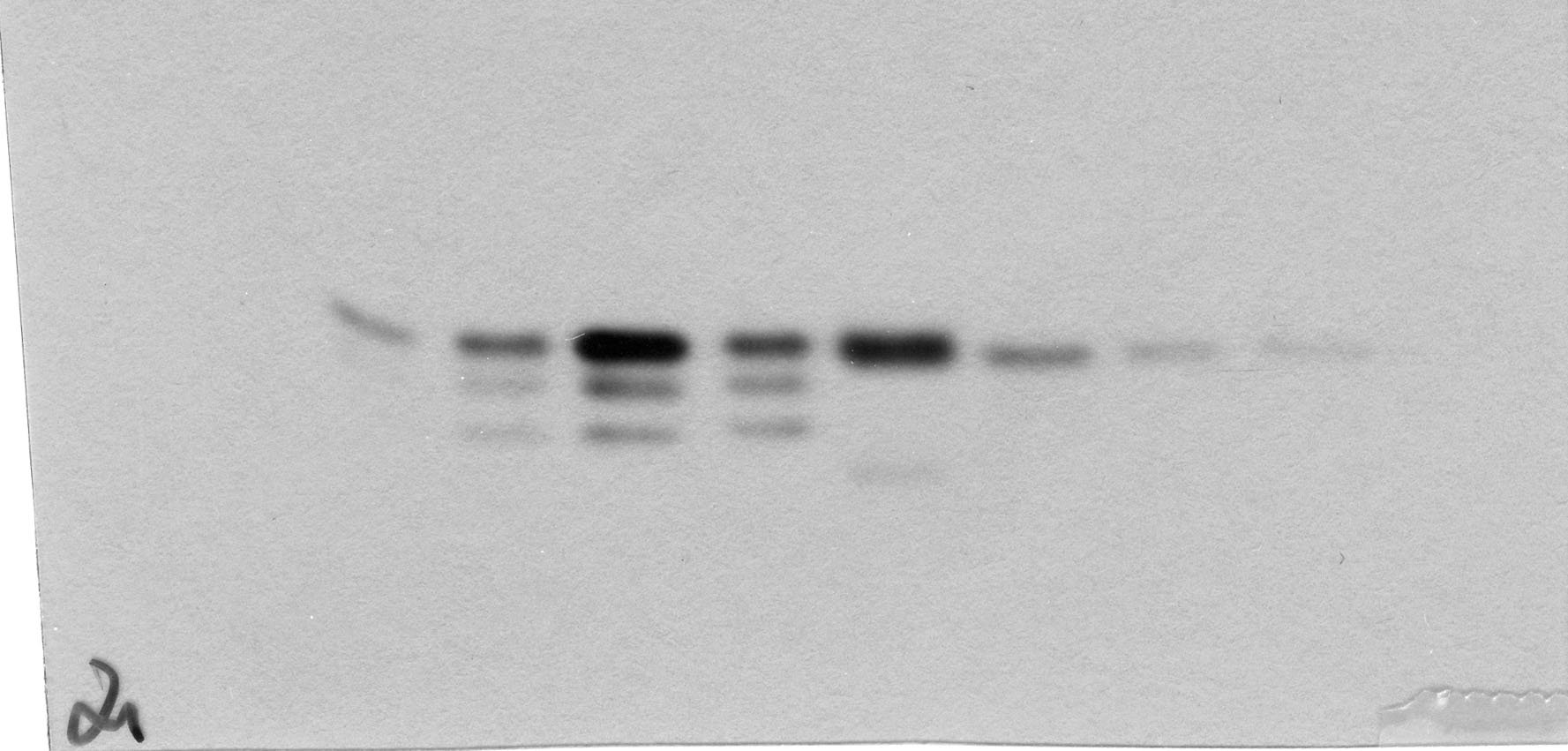

Supplement: Figure 3—source data 1. [file elife-84798-fig3-data1.zip › Figure 3-source data 10/Figure 3-source data 10.jpg]

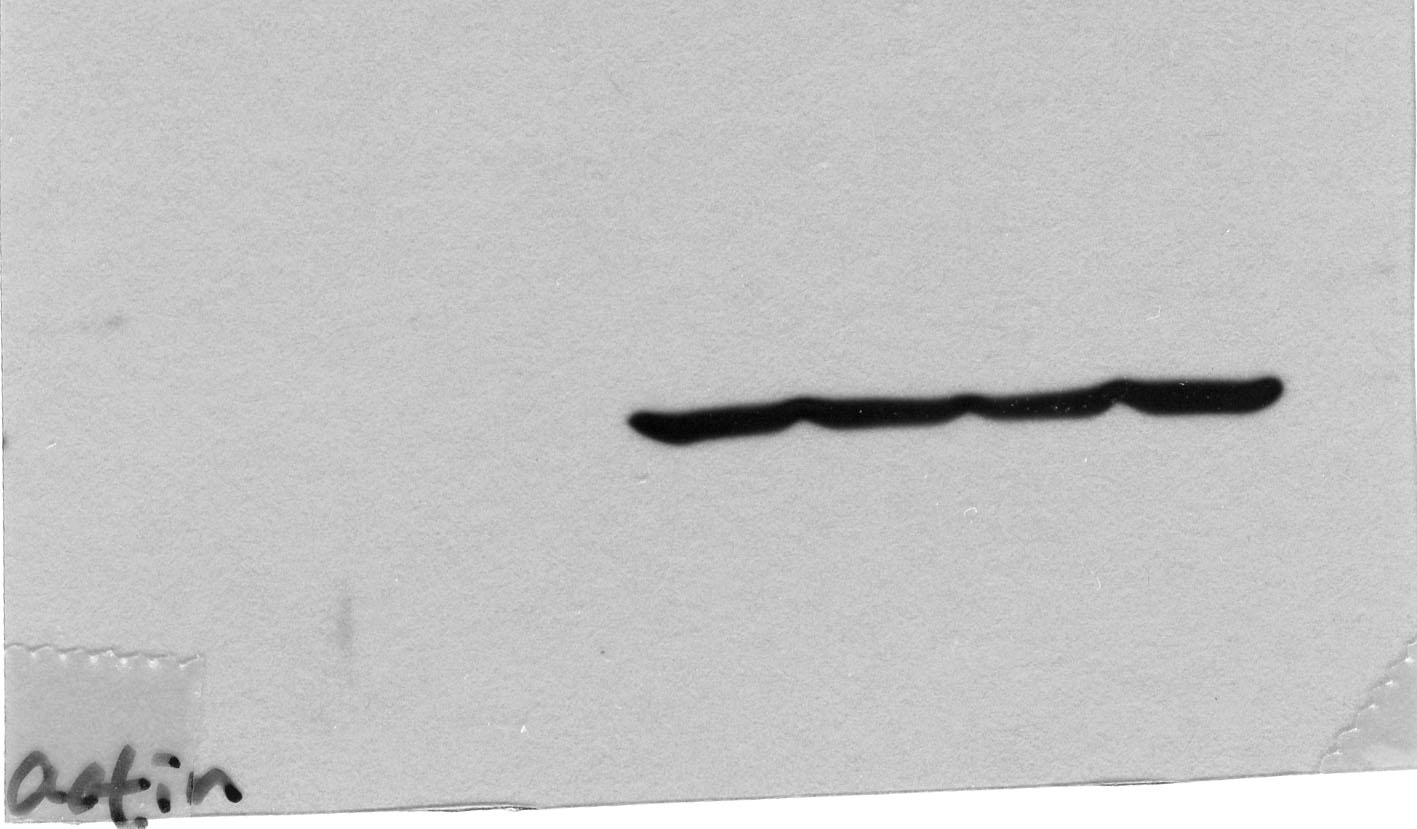

Supplement: Figure 3—source data 1. [file elife-84798-fig3-data1.zip › Figure 3-source data 11/Figure 3-source data 11.jpg]

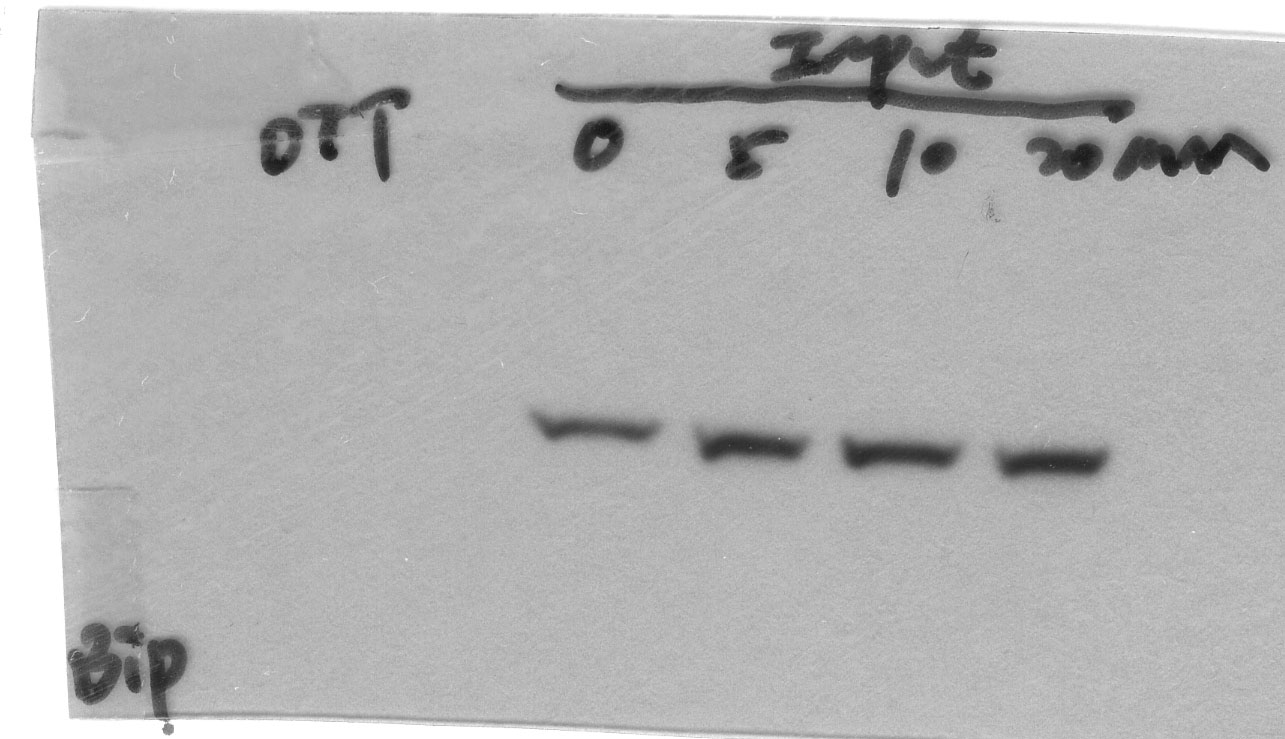

Supplement: Figure 3—source data 1. [file elife-84798-fig3-data1.zip › Figure 3-source data 12/Figure 3-source data 12.jpg]

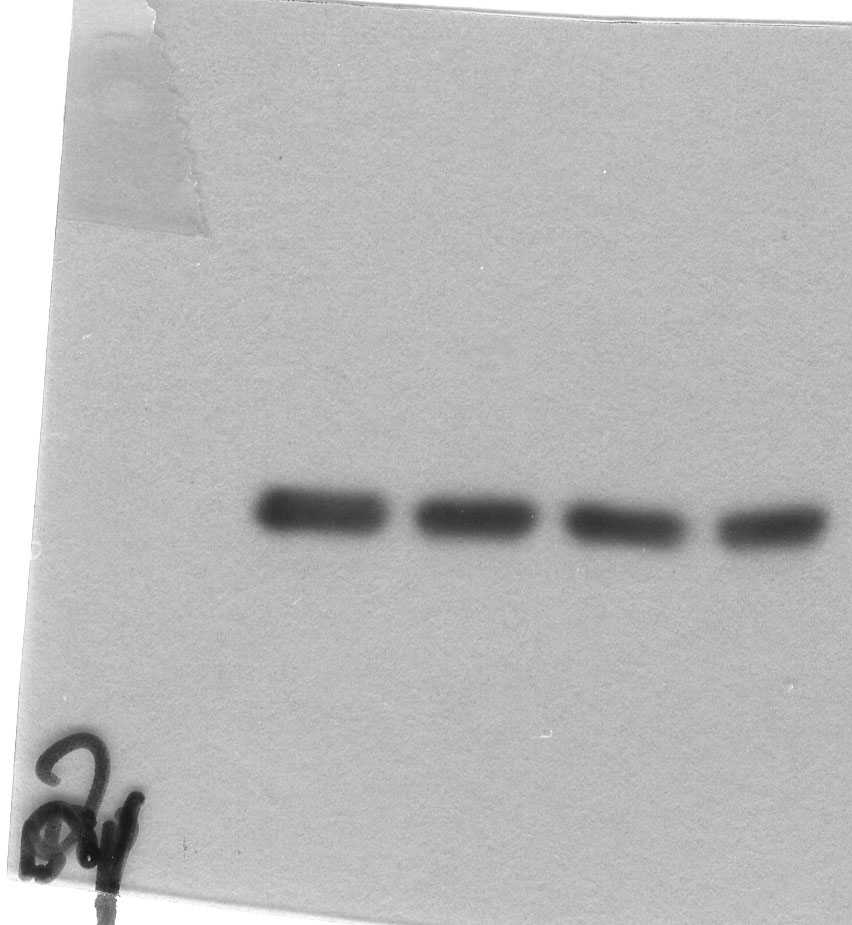

Supplement: Figure 3—source data 1. [file elife-84798-fig3-data1.zip › Figure 3-source data 13/Figure 3-source data 13.jpg]

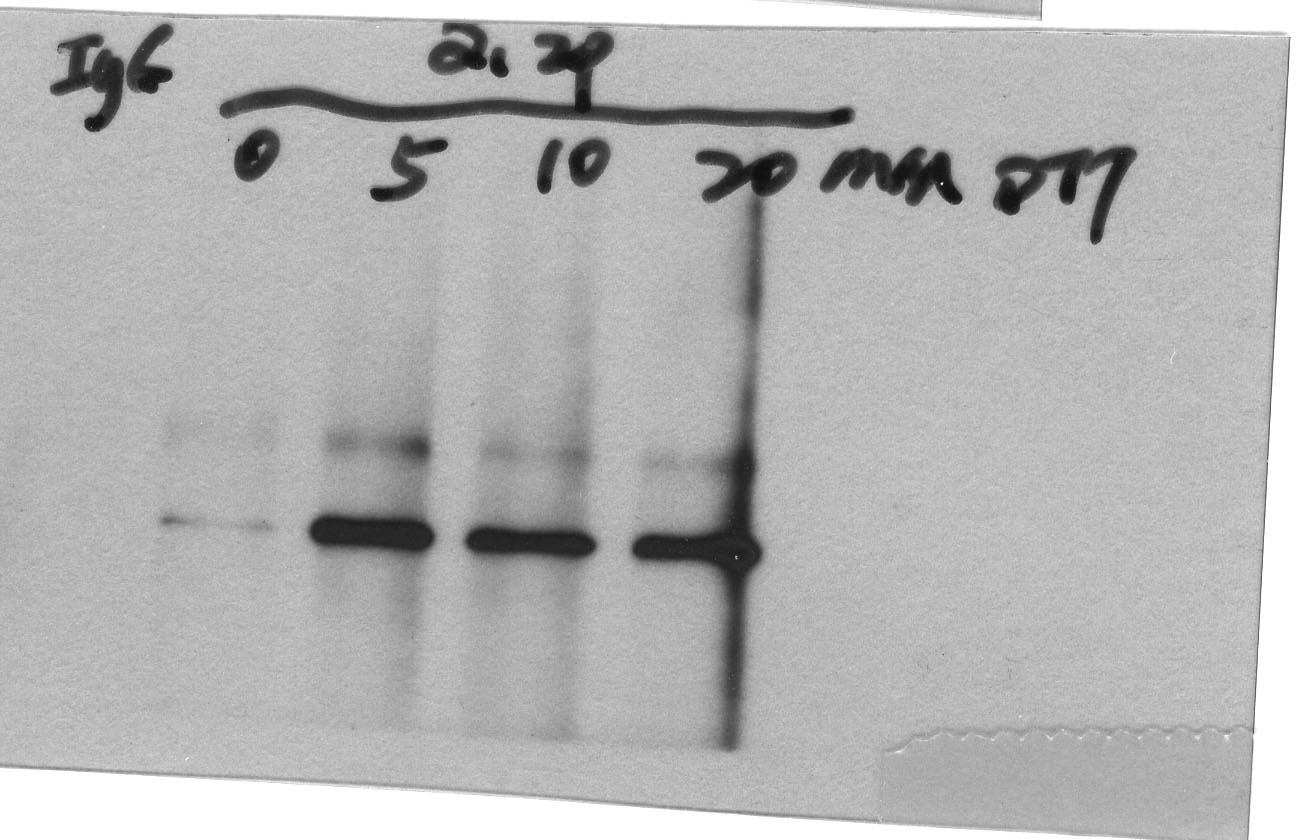

Supplement: Figure 3—source data 1. [file elife-84798-fig3-data1.zip › Figure 3-source data 14/Figure 3-source data 14.jpg]

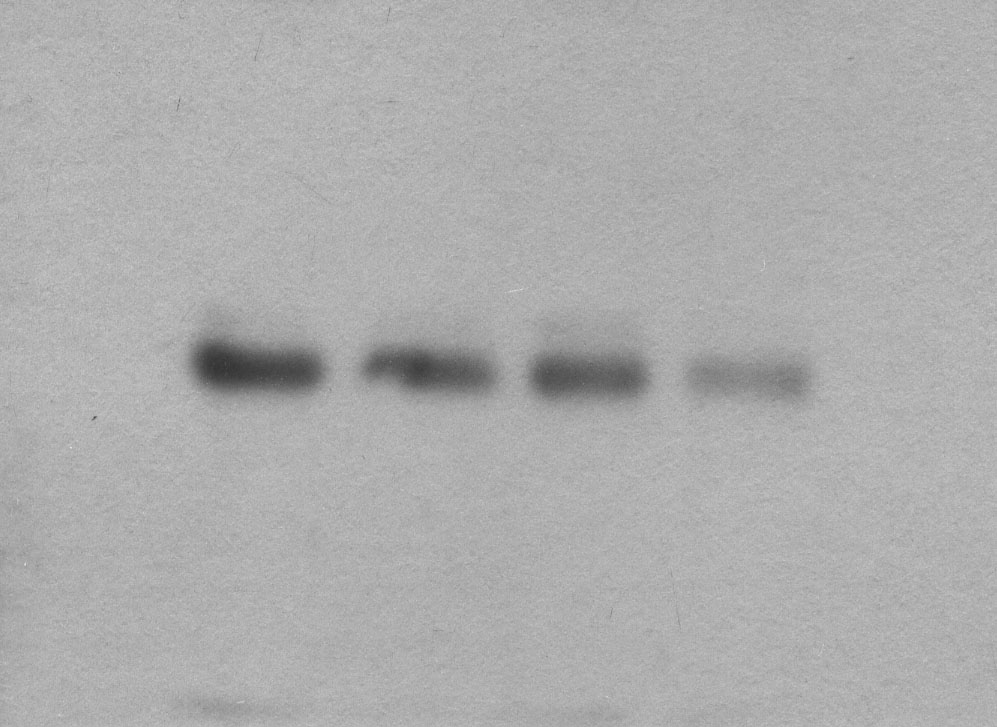

Supplement: Figure 3—source data 1. [file elife-84798-fig3-data1.zip › Figure 3-source data 15/Figure 3-source data 15.jpg]

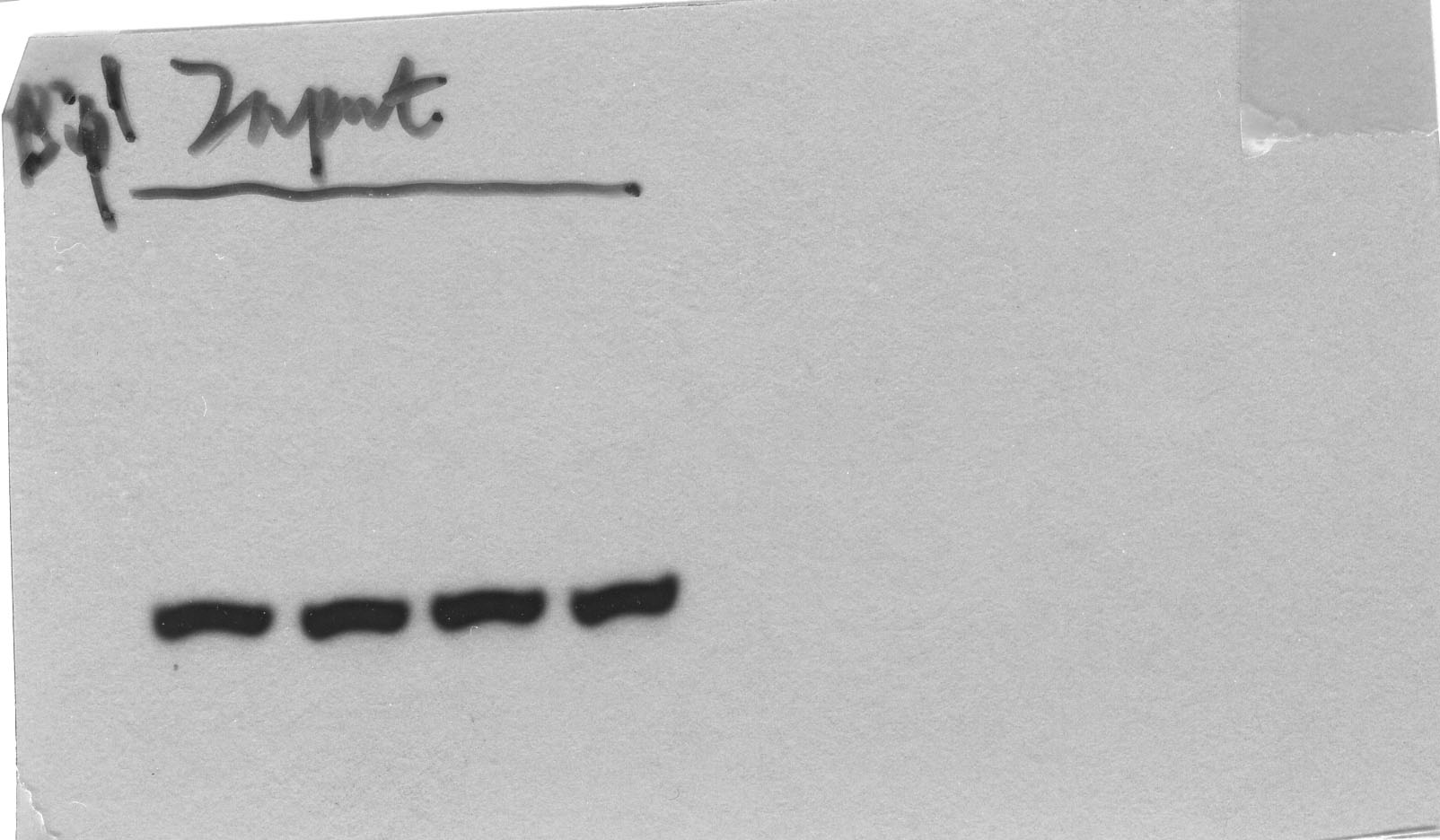

Supplement: Figure 3—source data 1. [file elife-84798-fig3-data1.zip › Figure 3-source data 16/Figure 3-source data 16.jpg]

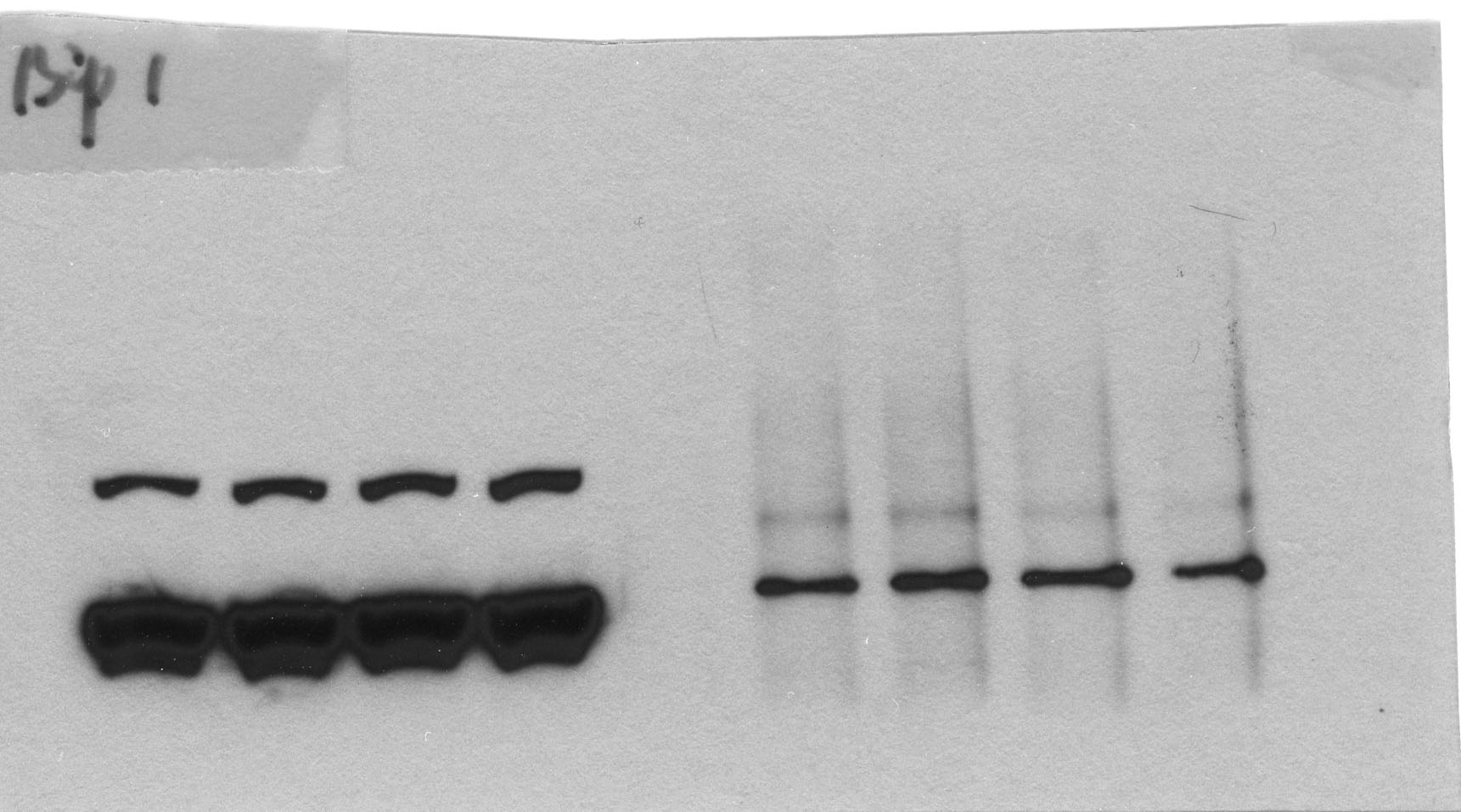

Supplement: Figure 3—source data 1. [file elife-84798-fig3-data1.zip › Figure 3-source data 17/Figure 3-source data 17.jpg]

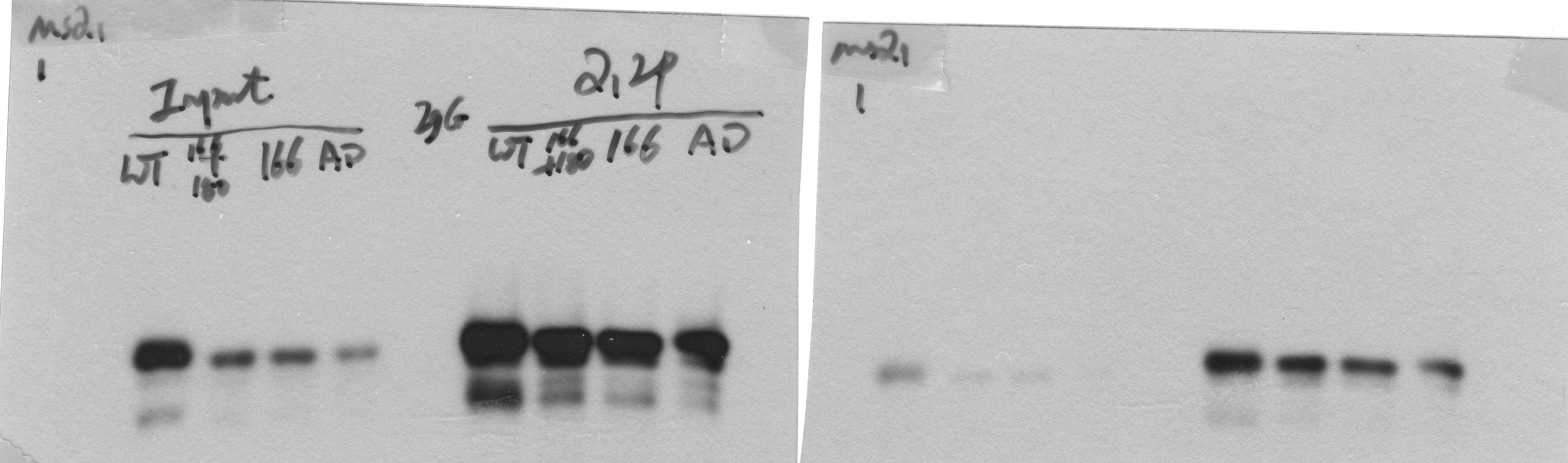

Supplement: Figure 3—source data 1. [file elife-84798-fig3-data1.zip › Figure 3-source data 18/Figure 3-source data 18.jpg]

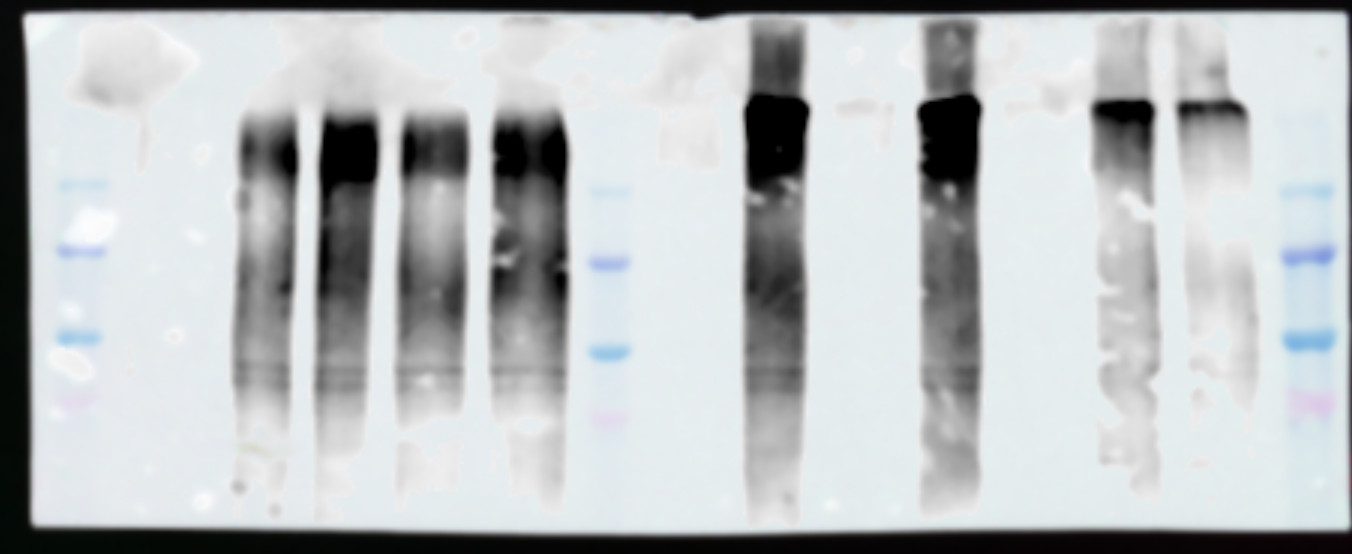

Supplement: Figure 3—figure supplement 1—source data 1. [file elife-84798-fig3-figsupp1-data1.zip › Figure 3-figure supplement 1-source data 1/Figure 3-figure supplement 1-source data 1.tif]

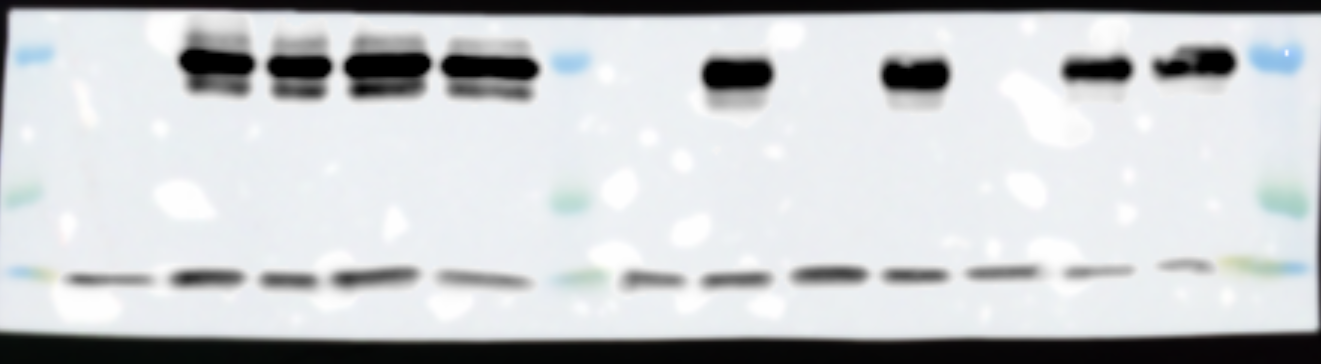

Supplement: Figure 3—figure supplement 1—source data 1. [file elife-84798-fig3-figsupp1-data1.zip › Figure 3-figure supplement 1-source data 2/Figure 3-figure supplement 1-source data 2.tif]

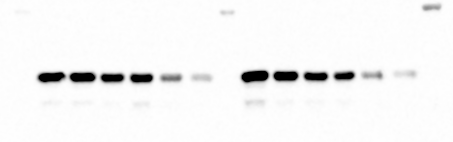

Supplement: Figure 3—figure supplement 1—source data 1. [file elife-84798-fig3-figsupp1-data1.zip › Figure 3-figure supplement 1-source data 3/Figure 3-figure supplement 1-source data 3.tif]

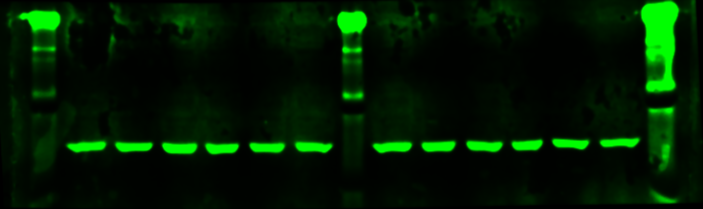

Supplement: Figure 3—figure supplement 1—source data 1. [file elife-84798-fig3-figsupp1-data1.zip › Figure 3-figure supplement 1-source data 4/Figure 3-figure supplement 1-source data 4.tif]

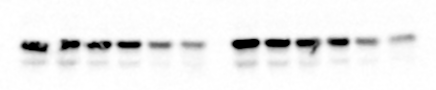

Supplement: Figure 3—figure supplement 1—source data 1. [file elife-84798-fig3-figsupp1-data1.zip › Figure 3-figure supplement 1-source data 5/Figure 3-figure supplement 1-source data 5.tif]

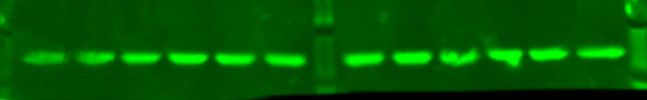

Supplement: Figure 3—figure supplement 1—source data 1. [file elife-84798-fig3-figsupp1-data1.zip › Figure 3-figure supplement 1-source data 6/Figure 3-figure supplement 1-source data 6.tif]

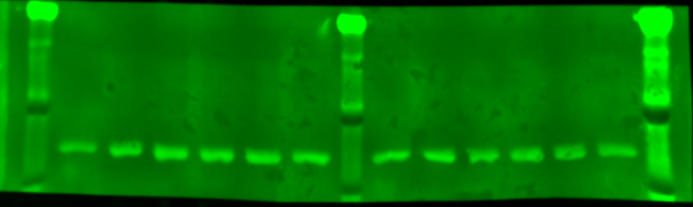

Supplement: Figure 3—figure supplement 1—source data 1. [file elife-84798-fig3-figsupp1-data1.zip › Figure 3-figure supplement 1-source data 7/Figure 3-figure supplement 1-source data 7.tif]

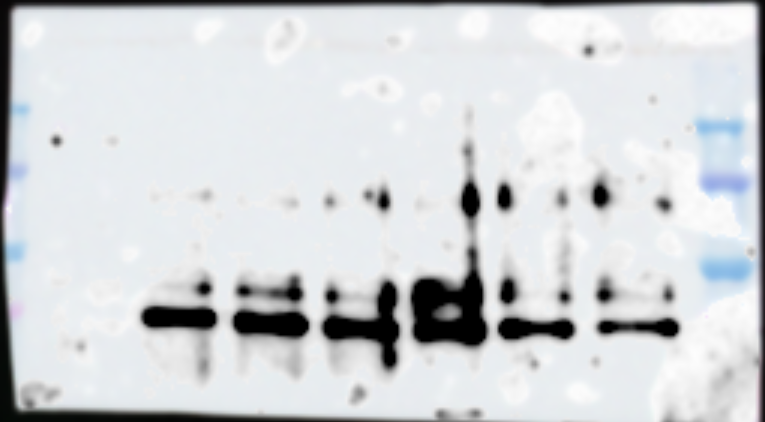

Supplement: Figure 3—figure supplement 1—source data 1. [file elife-84798-fig3-figsupp1-data1.zip › Figure 3-figure supplement 1-source data 8/Figure 3-figure supplement 1-source data 8.tif]

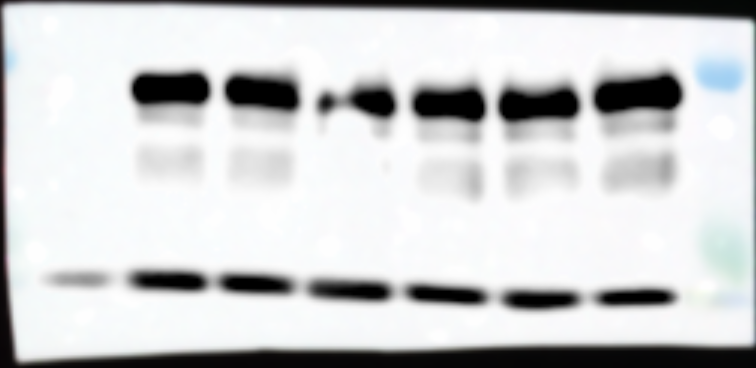

Supplement: Figure 3—figure supplement 1—source data 1. [file elife-84798-fig3-figsupp1-data1.zip › Figure 3-figure supplement 1-source data 9/Figure 3-figure supplement 1-source data 9.tif]

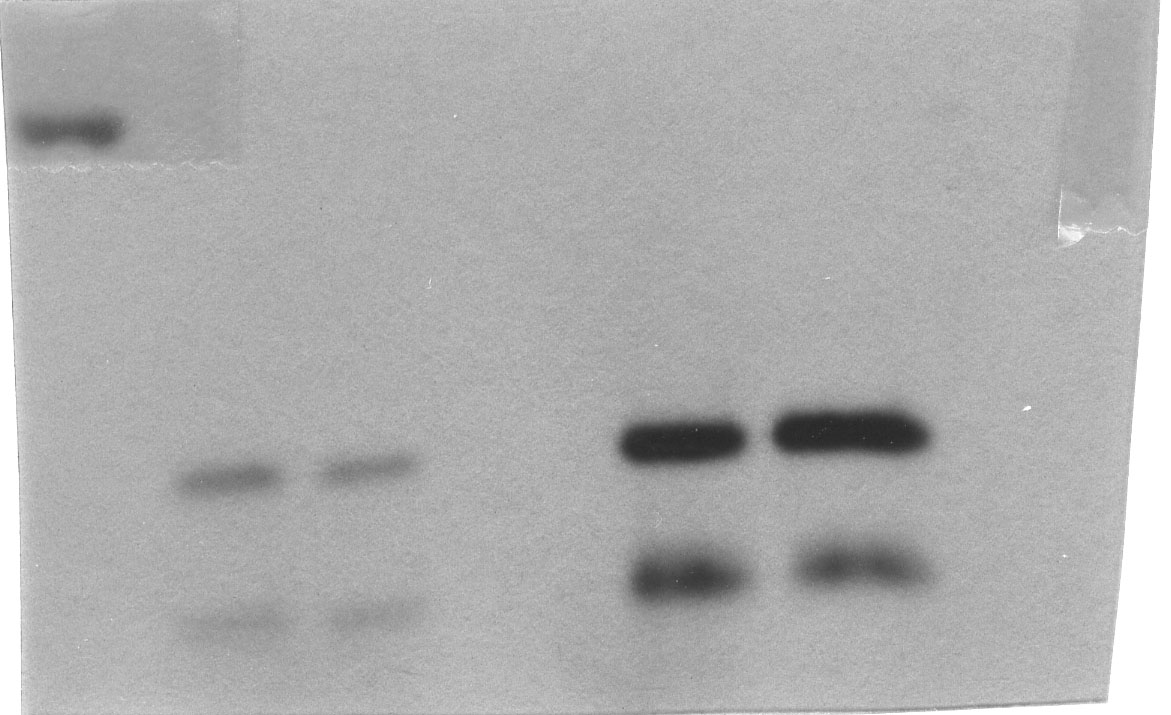

Supplement: Figure 4—source data 1. [file elife-84798-fig4-data1.zip › Figure 4-source data 1/Figure 4-source data 1.jpg]

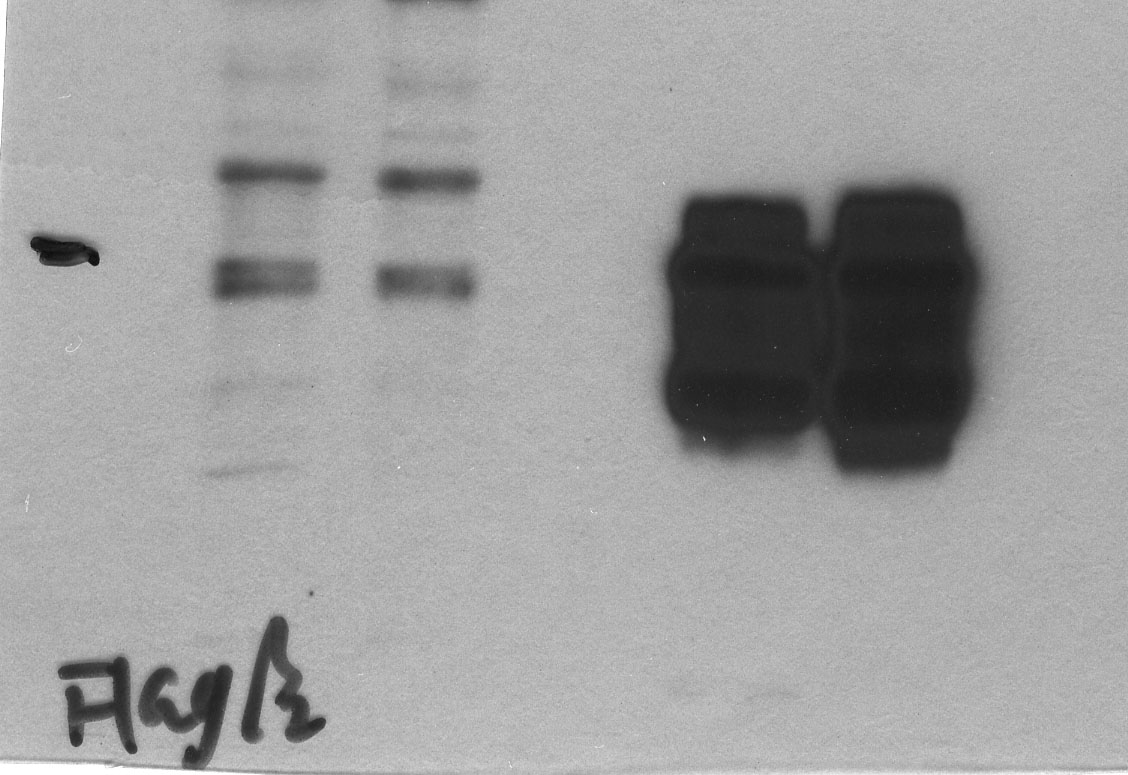

Supplement: Figure 4—source data 1. [file elife-84798-fig4-data1.zip › Figure 4-source data 2 2/Figure 4-source data 2.jpg]

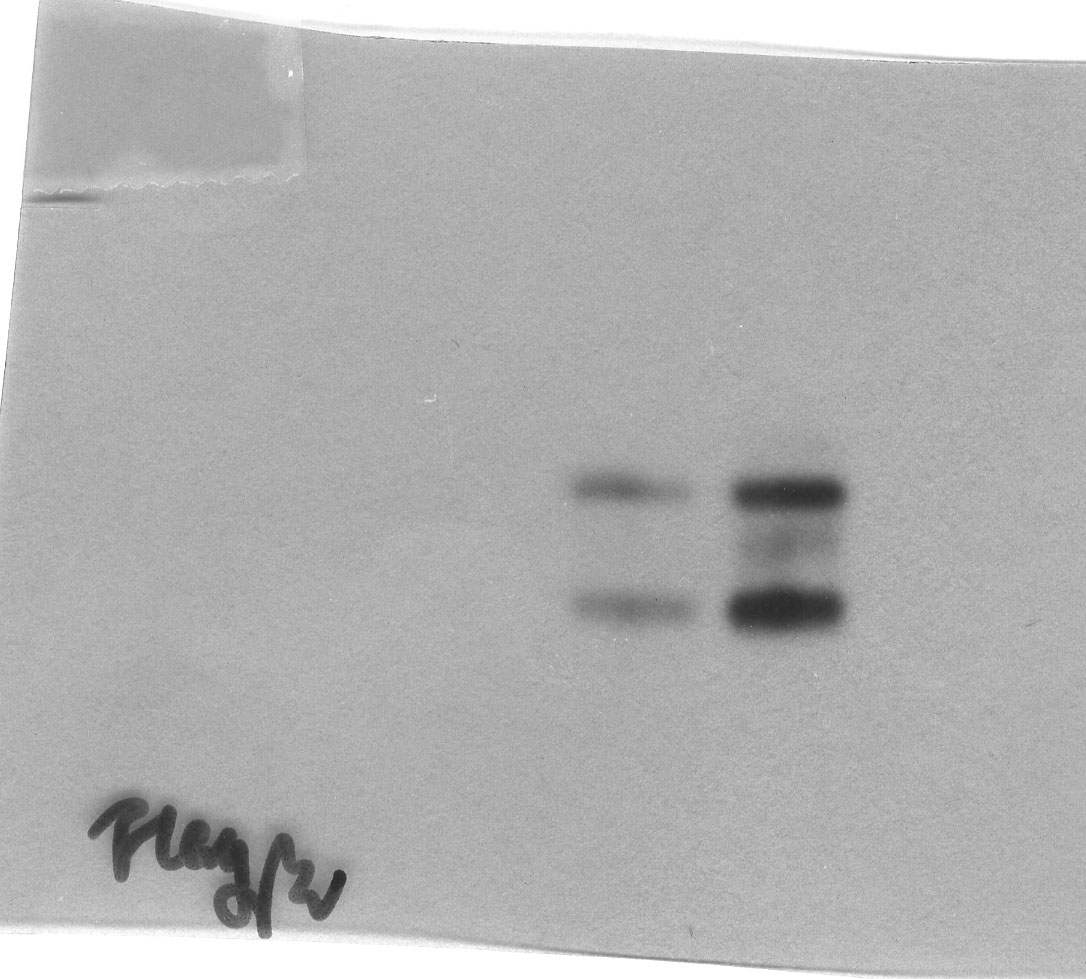

Supplement: Figure 4—source data 1. [file elife-84798-fig4-data1.zip › Figure 4-source data 3/Figure 4-source data 3.jpg]

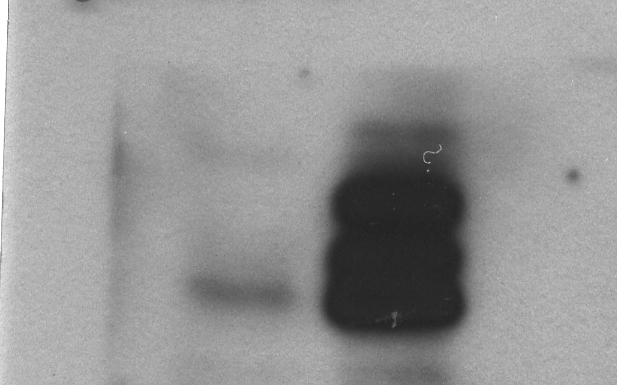

Supplement: Figure 4—source data 1. [file elife-84798-fig4-data1.zip › Figure 4-source data 4/Figure 4-source data 4.tif]

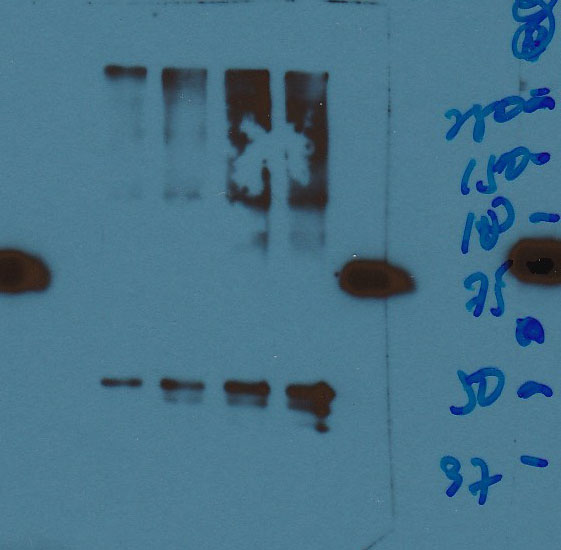

Supplement: Figure 4—source data 1. [file elife-84798-fig4-data1.zip › Figure 4-source data 5/Figure 4-source data 5.jpg]

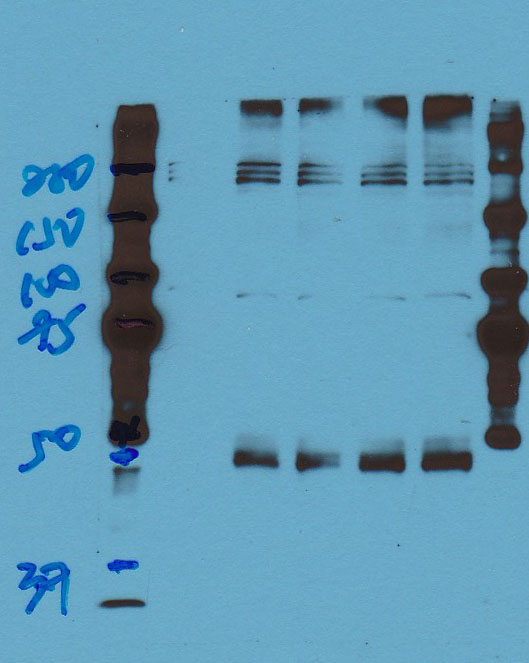

Supplement: Figure 4—source data 1. [file elife-84798-fig4-data1.zip › Figure 4-source data 6/Figure 4-source data 6.jpg]

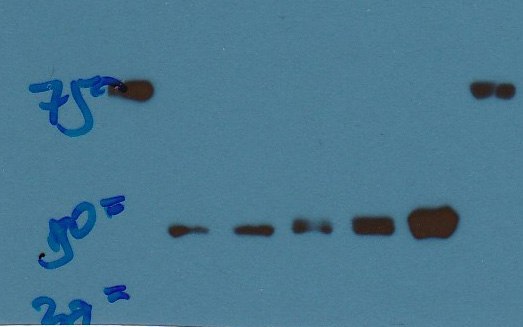

Supplement: Figure 4—source data 1. [file elife-84798-fig4-data1.zip › Figure 4-source data 7/Figure 4-source data 7.jpg]

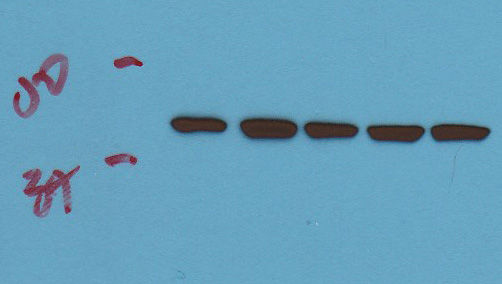

Supplement: Figure 4—source data 1. [file elife-84798-fig4-data1.zip › Figure 4-source data 8/Figure 4-source data 8.jpg]

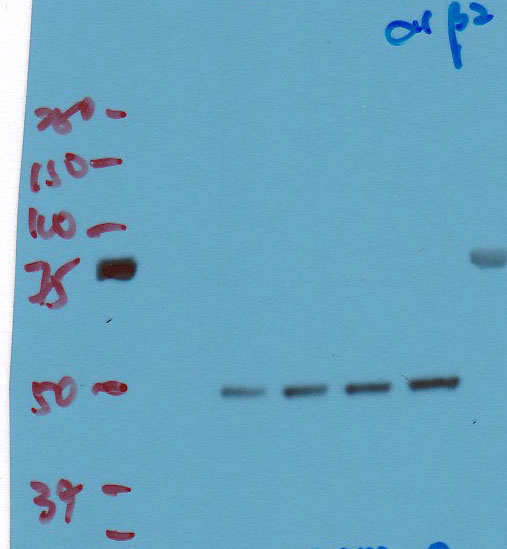

Supplement: Figure 4—source data 1. [file elife-84798-fig4-data1.zip › Figure 4-source data 9/Figure 4-source data 9.jpg]

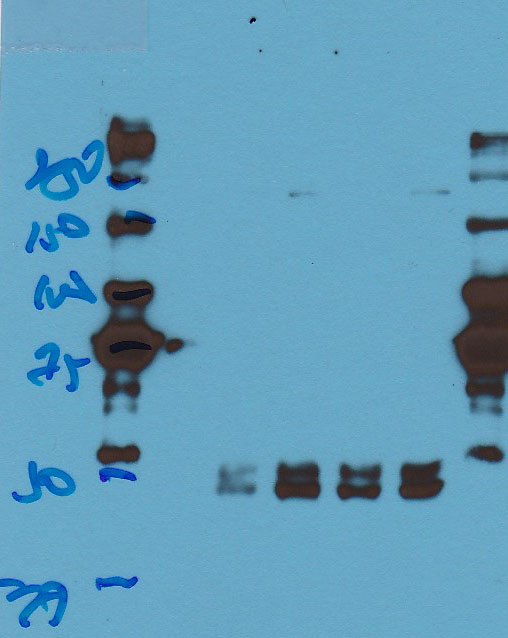

Supplement: Figure 4—source data 1. [file elife-84798-fig4-data1.zip › Figure 4-source data 10/Figure 4-source data 10.jpg]

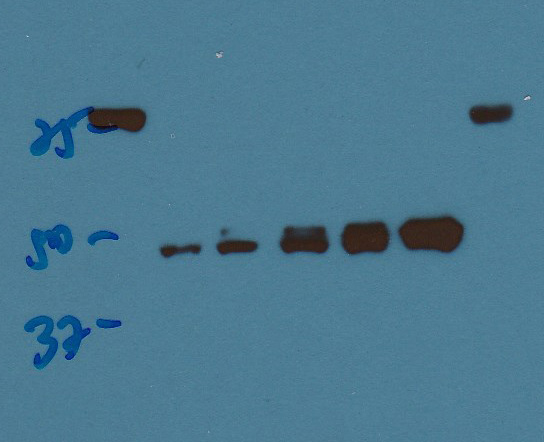

Supplement: Figure 4—source data 1. [file elife-84798-fig4-data1.zip › Figure 4-source data 11/Figure 4-source data 11.jpg]

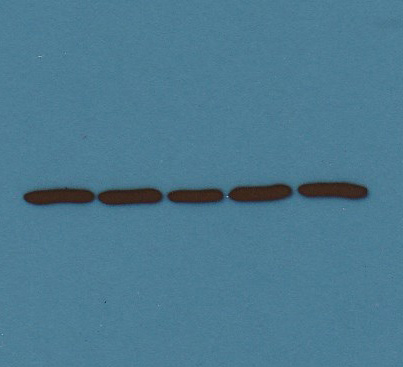

Supplement: Figure 4—source data 1. [file elife-84798-fig4-data1.zip › Figure 4-source data 12/Figure 4-source data 12.jpg]

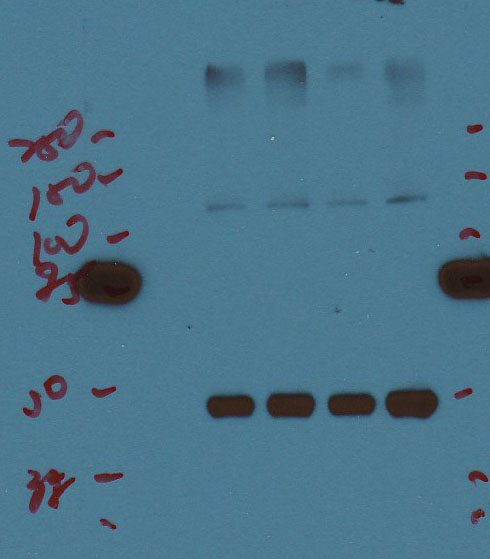

Supplement: Figure 4—source data 1. [file elife-84798-fig4-data1.zip › Figure 4-source data 13/Figure 4-source data 13.jpg]

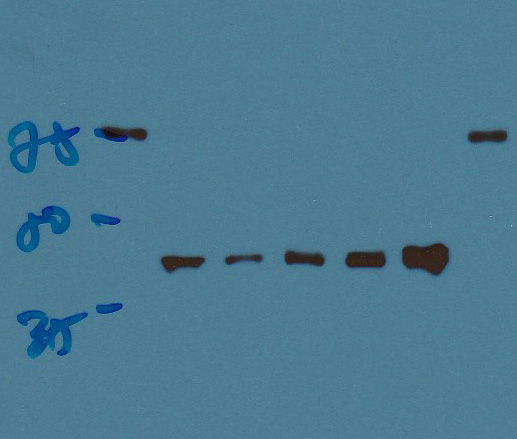

Supplement: Figure 4—source data 1. [file elife-84798-fig4-data1.zip › Figure 4-source data 14/Figure 4-source data 14.jpg]

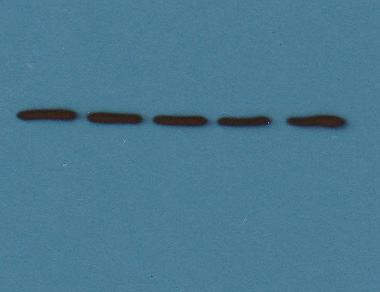

Supplement: Figure 4—source data 1. [file elife-84798-fig4-data1.zip › Figure 4-source data 15/Figure 4-source data 15.jpg]

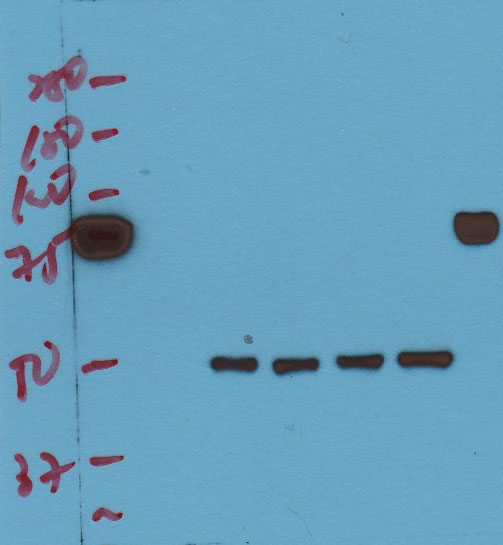

Supplement: Figure 4—source data 1. [file elife-84798-fig4-data1.zip › Figure 4-source data 16/Figure 4-source data 16.jpg]

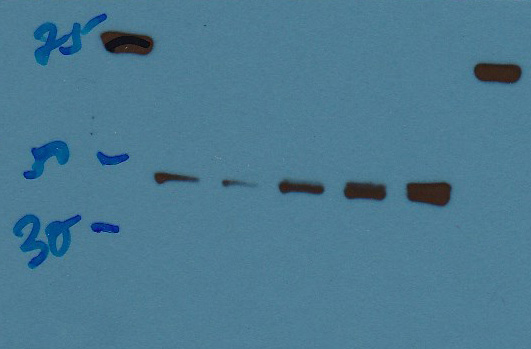

Supplement: Figure 4—source data 1. [file elife-84798-fig4-data1.zip › Figure 4-source data 17/Figure 4-source data 17.jpg]

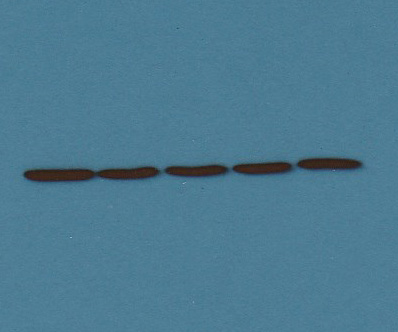

Supplement: Figure 4—source data 1. [file elife-84798-fig4-data1.zip › Figure 4-source data 18/Figure 4-source data 18.jpg]

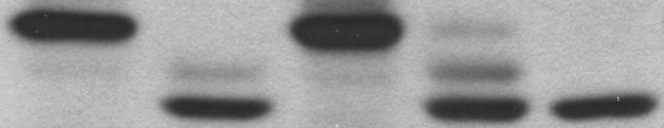

Supplement: Figure 5—source data 1. [file elife-84798-fig5-data1.zip › Figure 5-source data 1/Figure 5-source data 1.jpg]

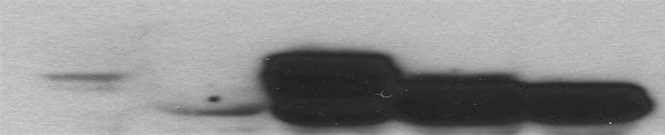

Supplement: Figure 5—source data 1. [file elife-84798-fig5-data1.zip › Figure 5-source data 2/Figure 5-source data 2.tif]

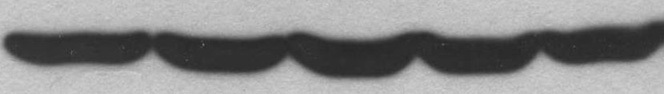

Supplement: Figure 5—source data 1. [file elife-84798-fig5-data1.zip › Figure 5-source data 3/Figure 5-source data 3.jpg]

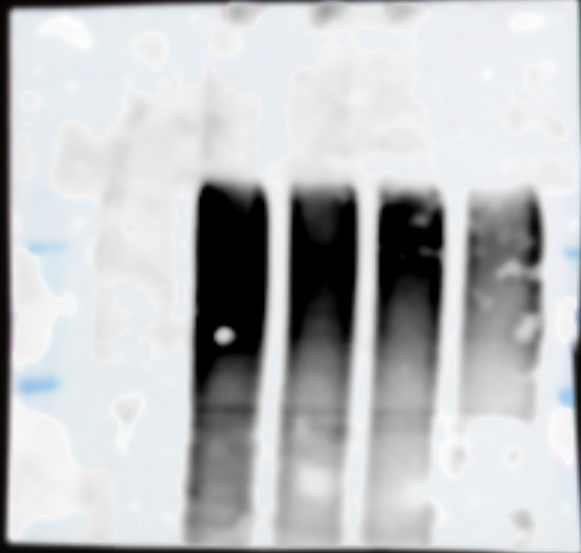

Supplement: Figure 5—source data 1. [file elife-84798-fig5-data1.zip › Figure 5-source data 4/Figure 5-source data 4.tif]

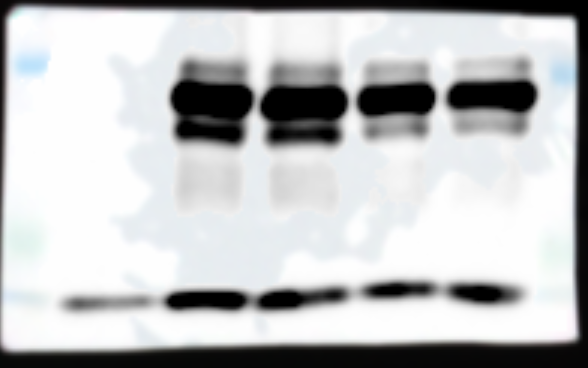

Supplement: Figure 5—source data 1. [file elife-84798-fig5-data1.zip › Figure 5-source data 5/Figure 5-source data 5.tif]

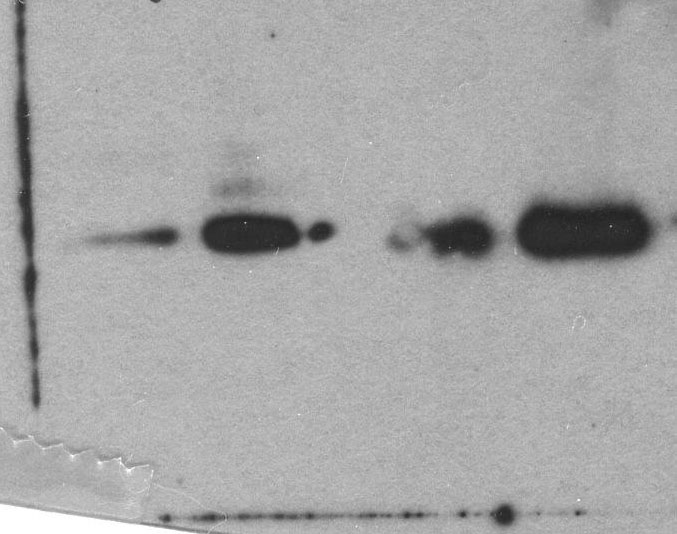

Supplement: Figure 5—source data 1. [file elife-84798-fig5-data1.zip › Figure 5-source data 6/Figure 5-source data 6.jpg]

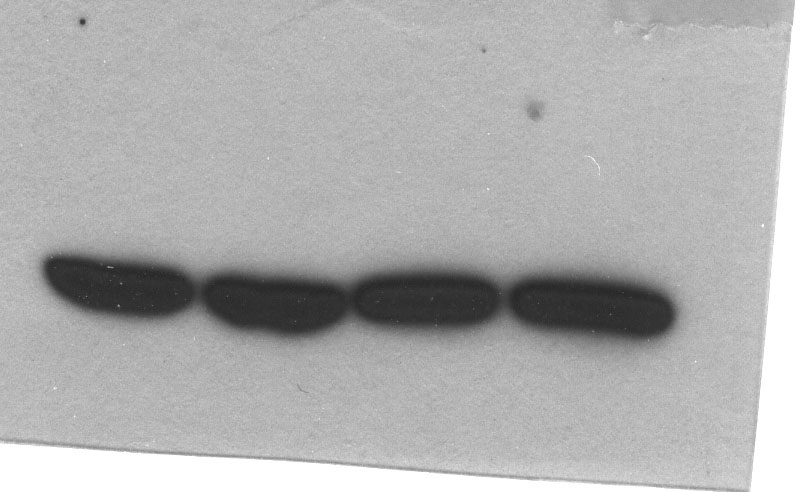

Supplement: Figure 5—source data 1. [file elife-84798-fig5-data1.zip › Figure 5-source data 7/Figure 5-source data 7.jpg]

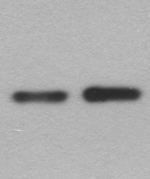

Supplement: Figure 5—source data 1. [file elife-84798-fig5-data1.zip › Figure 5-source data 8/Figure 5-source data 8.tif]

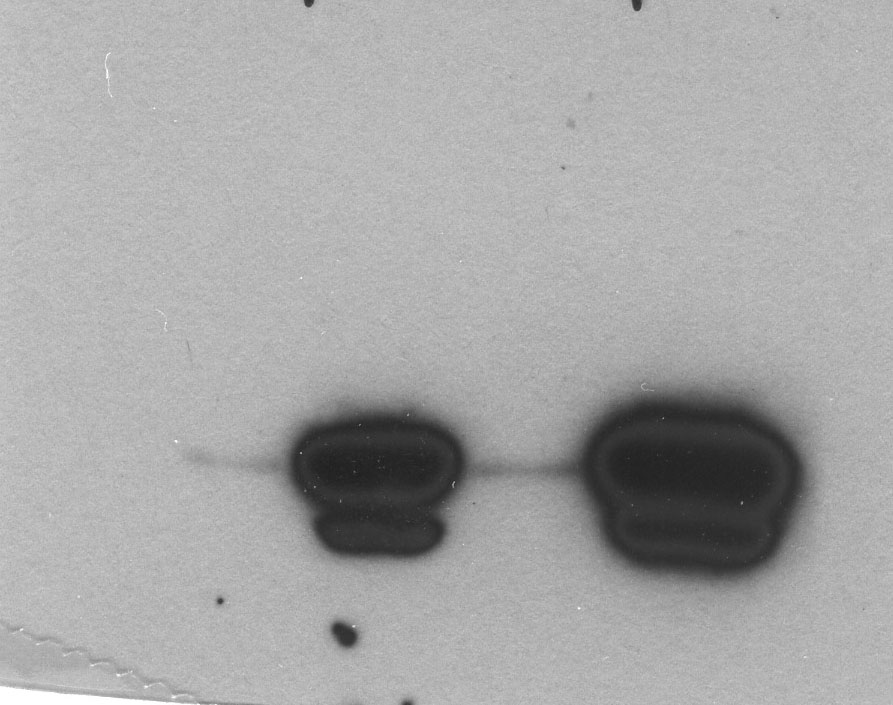

Supplement: Figure 5—source data 1. [file elife-84798-fig5-data1.zip › Figure 5-source data 9/Figure 5-source data 9.jpg]

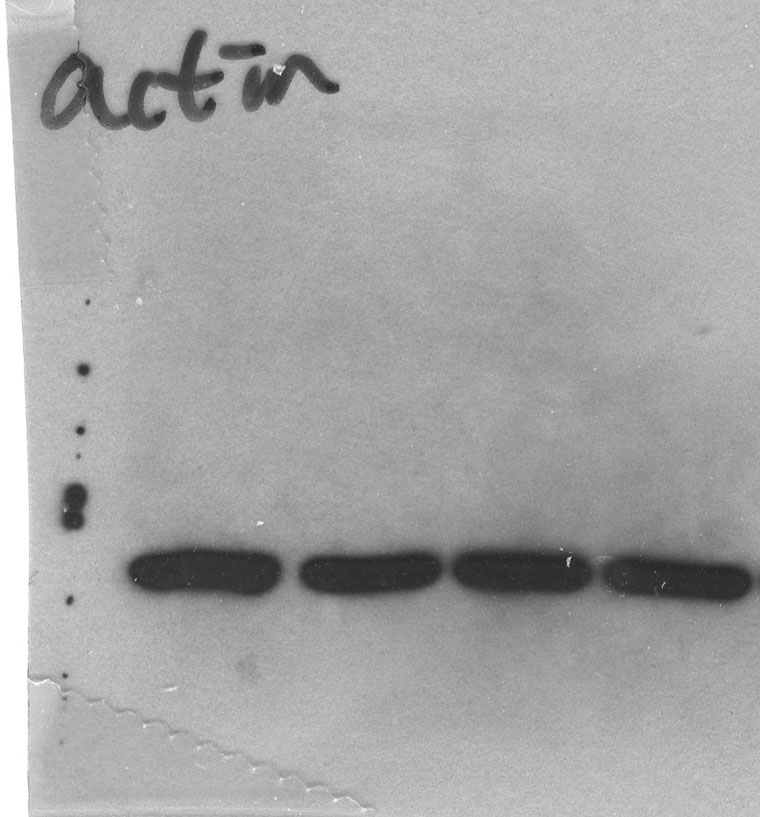

Supplement: Figure 5—source data 1. [file elife-84798-fig5-data1.zip › Figure 5-source data 10/Figure 5-source data 10.jpg]

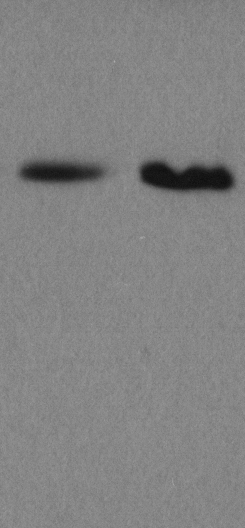

Supplement: Figure 5—source data 1. [file elife-84798-fig5-data1.zip › Figure 5-source data 11/Figure 5-source data 11.tif]

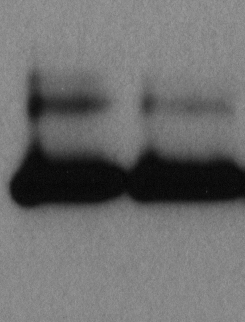

Supplement: Figure 5—source data 1. [file elife-84798-fig5-data1.zip › Figure 5-source data 12/Figure 5-source data 12.tif]

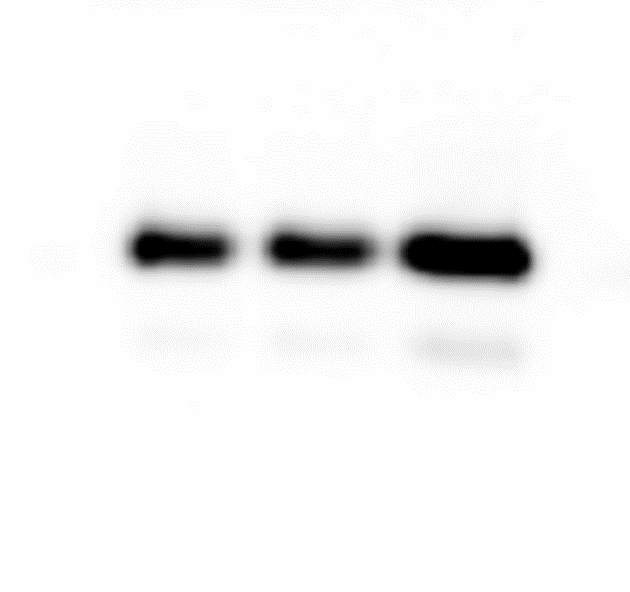

Supplement: Figure 5—source data 1. [file elife-84798-fig5-data1.zip › Figure 5-source data 13/Figure 5-source data 13.tif]

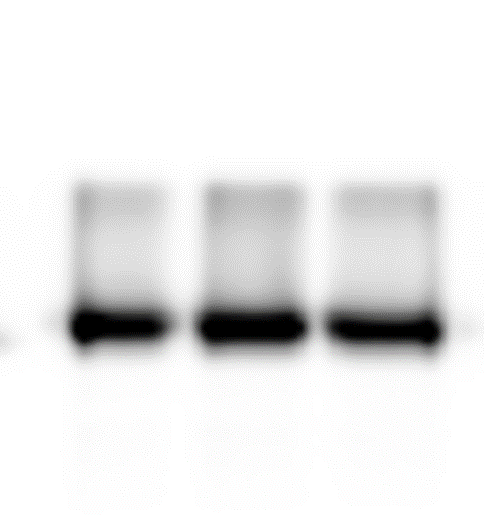

Supplement: Figure 5—source data 1. [file elife-84798-fig5-data1.zip › Figure 5-source data 14/Figure 5-source data 14.tif]

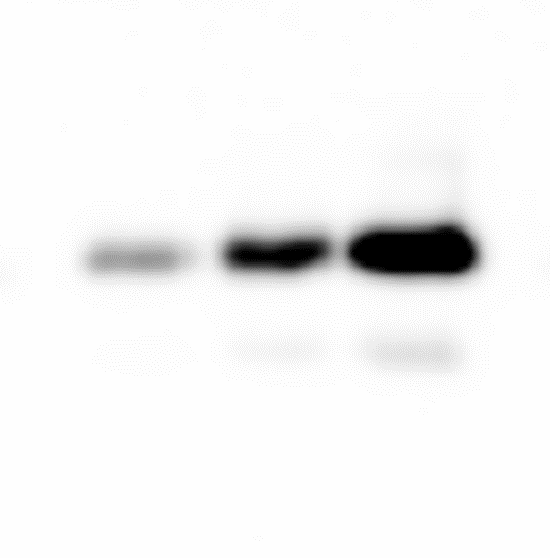

Supplement: Figure 5—source data 1. [file elife-84798-fig5-data1.zip › Figure 5-source data 15/Figure 5-source data 15.tif]

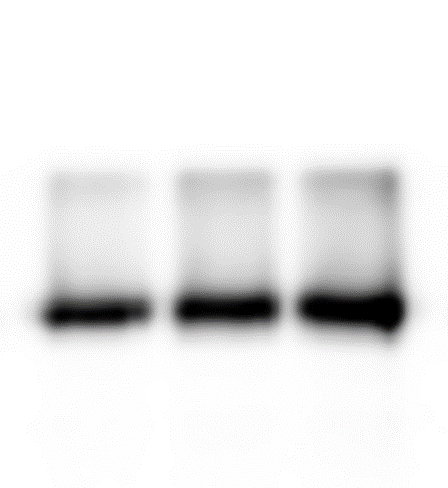

Supplement: Figure 5—source data 1. [file elife-84798-fig5-data1.zip › Figure 5-source data 16/Figure 5-source data 16.tif]

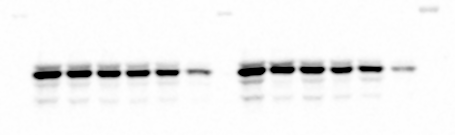

Supplement: Figure 5—figure supplement 1—source data 1. [file elife-84798-fig5-figsupp1-data1.zip › Figure 5-figure supplement 1-source data 1/Figure 5-figure supplement 1-source data 1.tif]

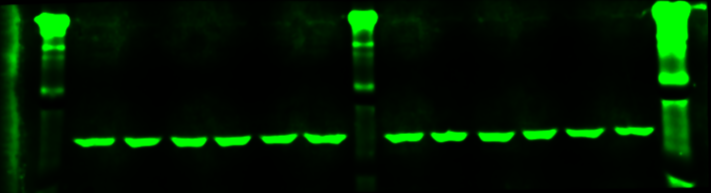

Supplement: Figure 5—figure supplement 1—source data 1. [file elife-84798-fig5-figsupp1-data1.zip › Figure 5-figure supplement 1-source data 2/Figure 5-figure supplement 1-source data 2.tif]

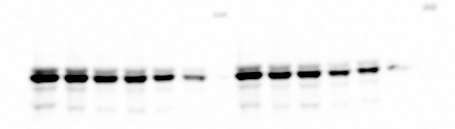

Supplement: Figure 5—figure supplement 1—source data 1. [file elife-84798-fig5-figsupp1-data1.zip › Figure 5-figure supplement 1-source data 3/Figure 5-figure supplement 1-source data 3.tif]

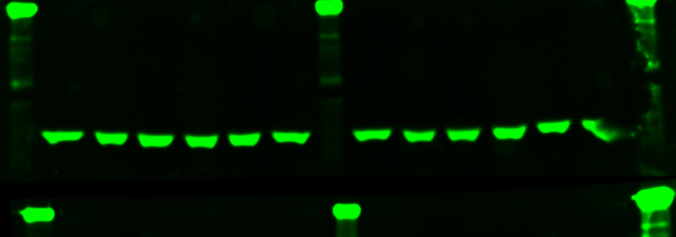

Supplement: Figure 5—figure supplement 1—source data 1. [file elife-84798-fig5-figsupp1-data1.zip › Figure 5-figure supplement 1-source data 4/Figure 5-figure supplement 1-source data 4.tif]

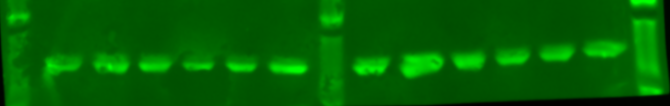

Supplement: Figure 5—figure supplement 1—source data 1. [file elife-84798-fig5-figsupp1-data1.zip › Figure 5-figure supplement 1-source data 5/Figure 5-figure supplement 1-source data 5.tif]

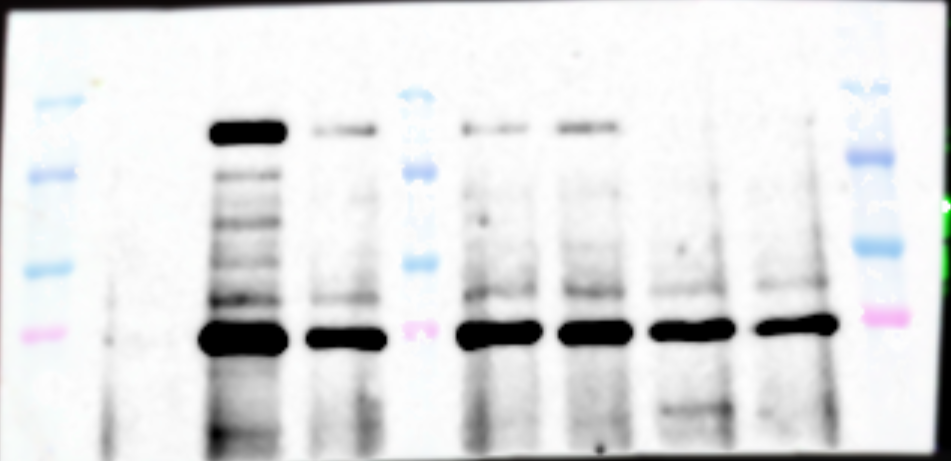

Supplement: Figure 5—figure supplement 1—source data 1. [file elife-84798-fig5-figsupp1-data1.zip › Figure 5-figure supplement 1-source data 6/Figure 5-figure supplement 1-source data 6.tif]

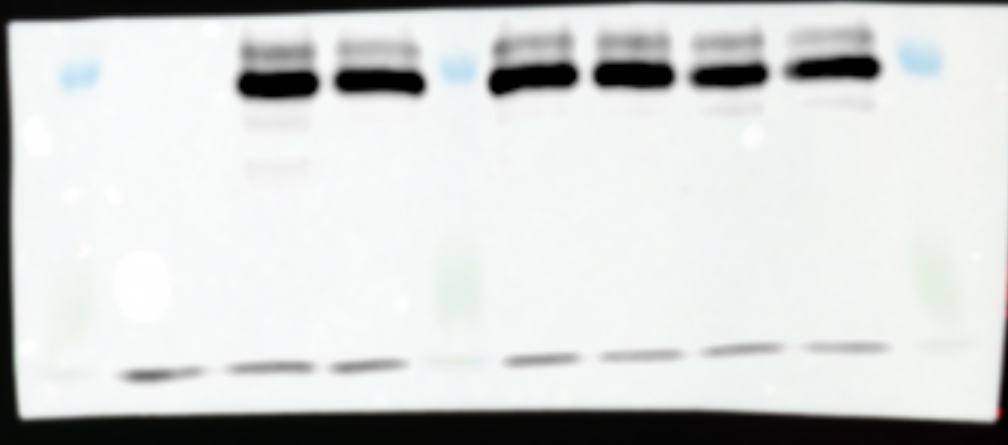

Supplement: Figure 5—figure supplement 1—source data 1. [file elife-84798-fig5-figsupp1-data1.zip › Figure 5-figure supplement 1-source data 7/Figure 5-figure supplement 1-source data 7.tif]

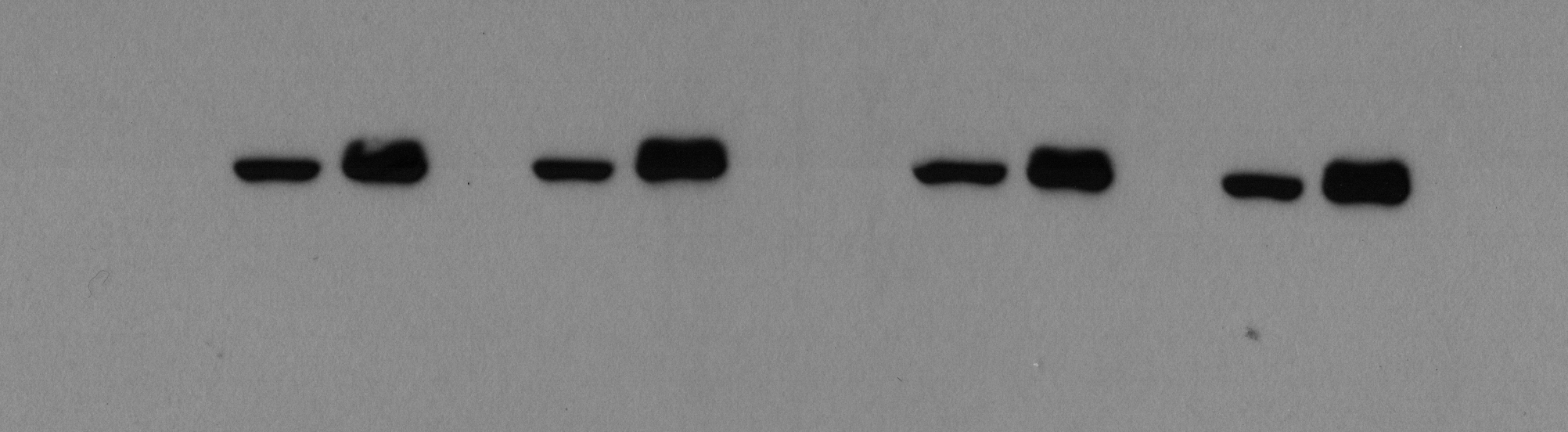

Supplement: Figure 5—figure supplement 2—source data 1. [file elife-84798-fig5-figsupp2-data1.zip › Figure 5-figure supplement 2-source data 1/Figure 5-figure supplement 2-source data 1.tif]

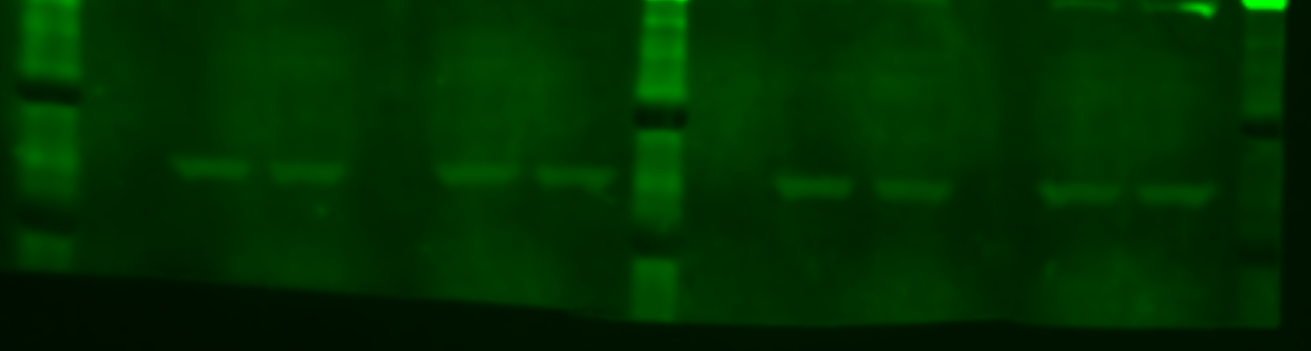

Supplement: Figure 5—figure supplement 2—source data 1. [file elife-84798-fig5-figsupp2-data1.zip › Figure 5-figure supplement 2-source data 2/Figure 5-figure supplement 2-source data 2.tif]

Figure 5—figure supplement 2

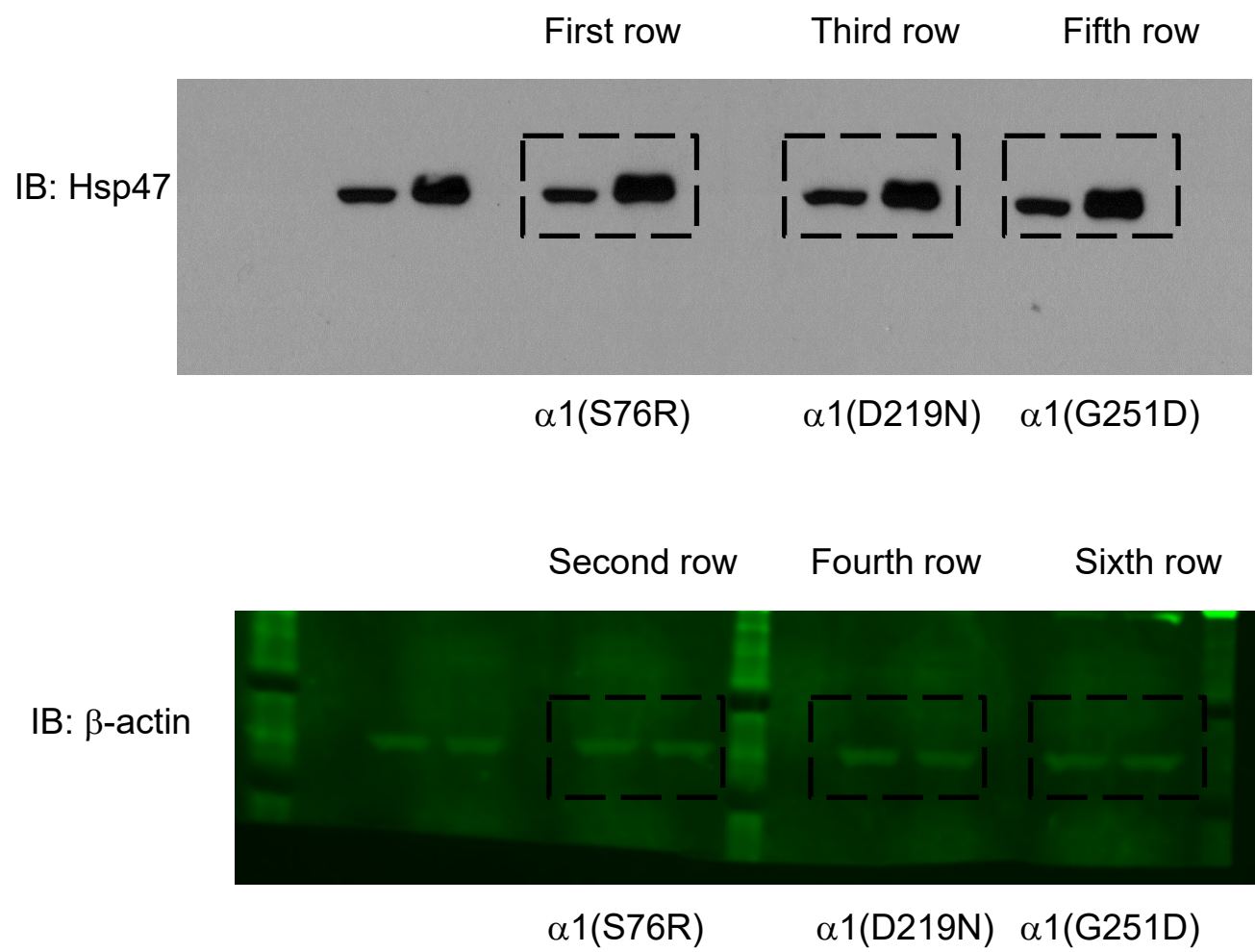

Supplement: Figure 5—figure supplement 2—source data 2. [file elife-84798-fig5-figsupp2-data2.zip › Figure 5-figure supplement 2-source data 3/Figure 5-figure supplement 2-source data 3.pdf]

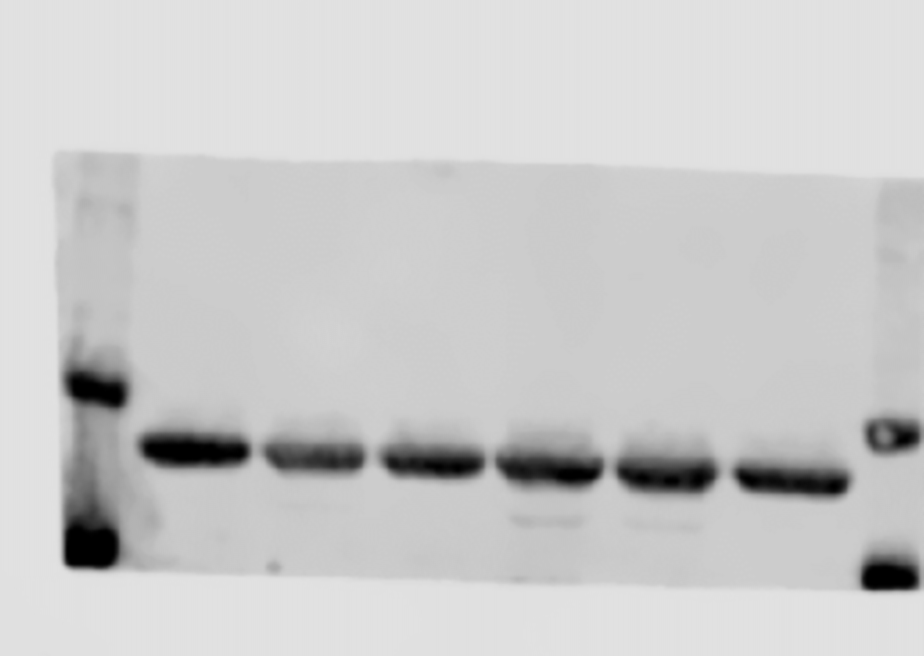

Supplement: Figure 6—source data 1. [file elife-84798-fig6-data1.zip › Figure 6-source data 1/Figure 6-source data 1.tif]

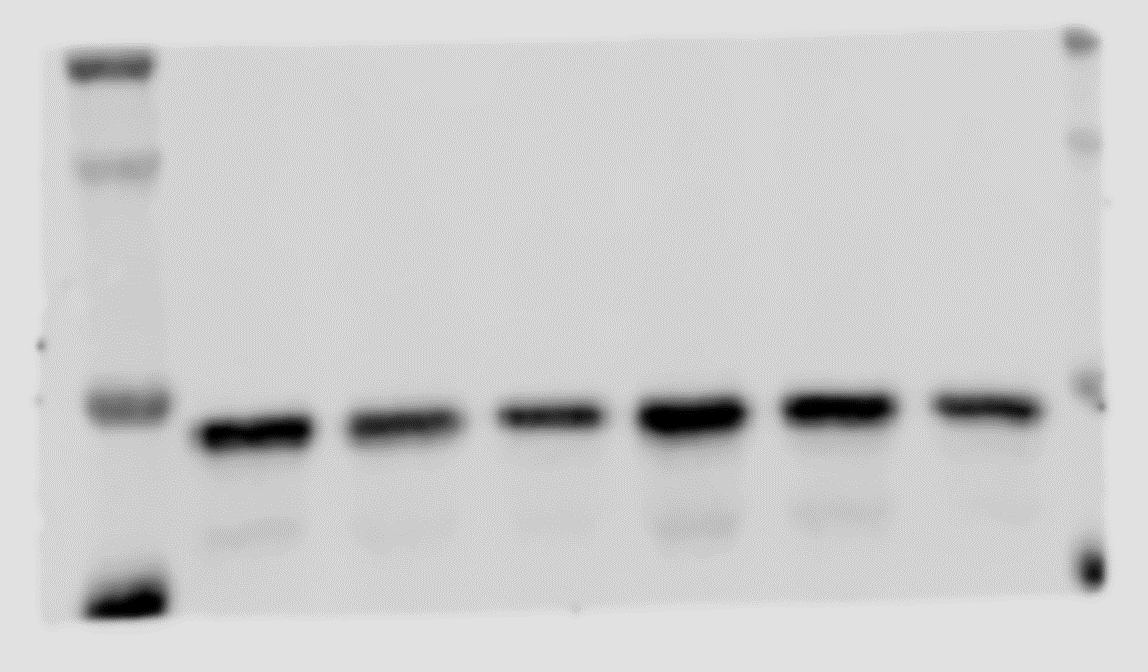

Supplement: Figure 6—source data 1. [file elife-84798-fig6-data1.zip › Figure 6-source data 2/Figure 6-source data 2.tif]

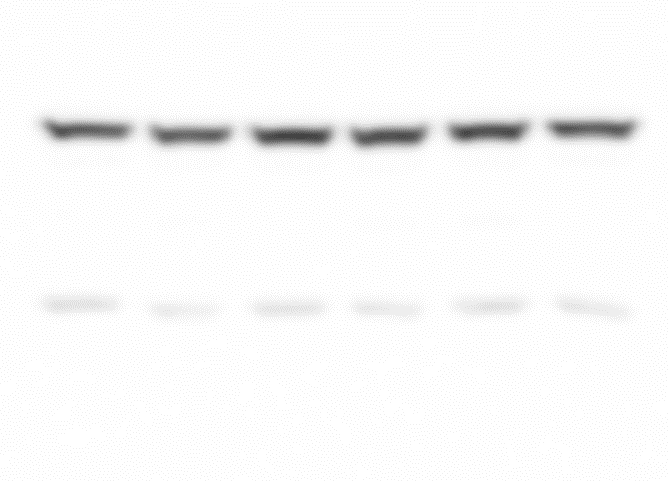

Supplement: Figure 6—source data 1. [file elife-84798-fig6-data1.zip › Figure 6-source data 3/Figure 6-source data 3.tif]

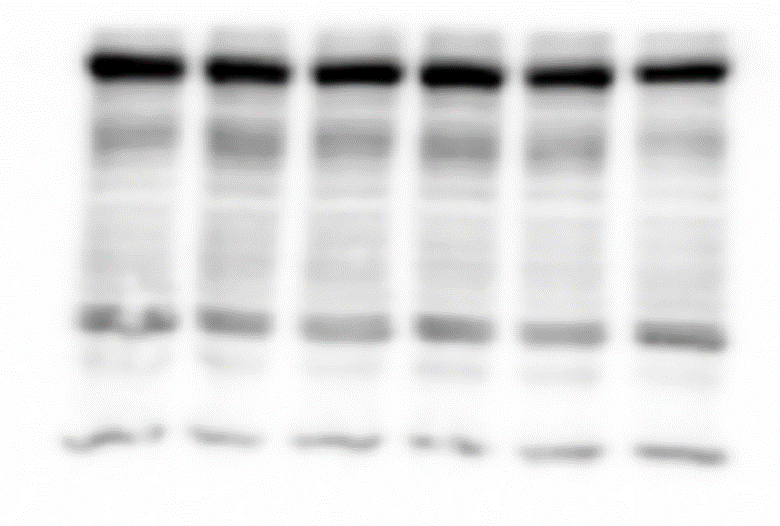

Supplement: Figure 6—source data 1. [file elife-84798-fig6-data1.zip › Figure 6-source data 4/Figure 6-source data 4.tif]

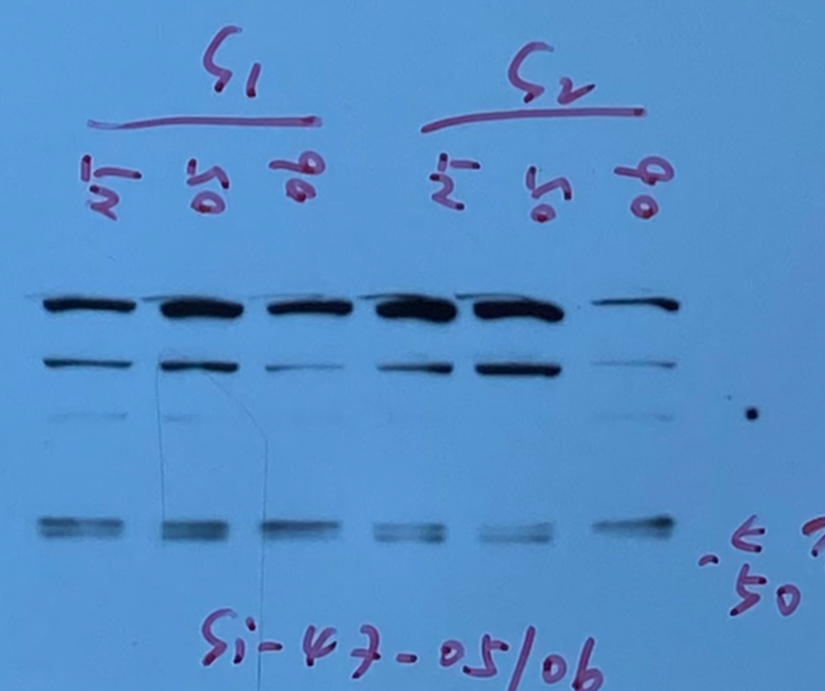

Supplement: Figure 6—source data 1. [file elife-84798-fig6-data1.zip › Figure 6-source data 5/Figure 6-source data 5.tif]

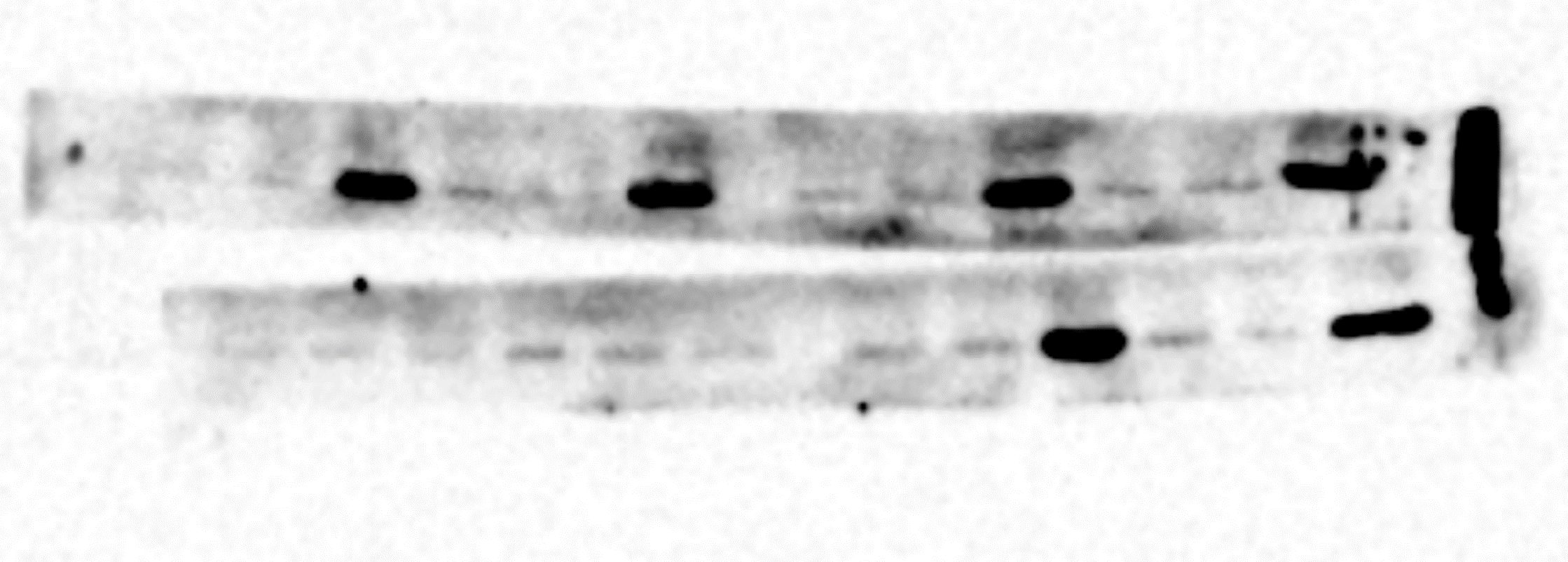

Supplement: Figure 6—source data 1. [file elife-84798-fig6-data1.zip › Figure 6-source data 6/Figure 6-source data 6.tif]

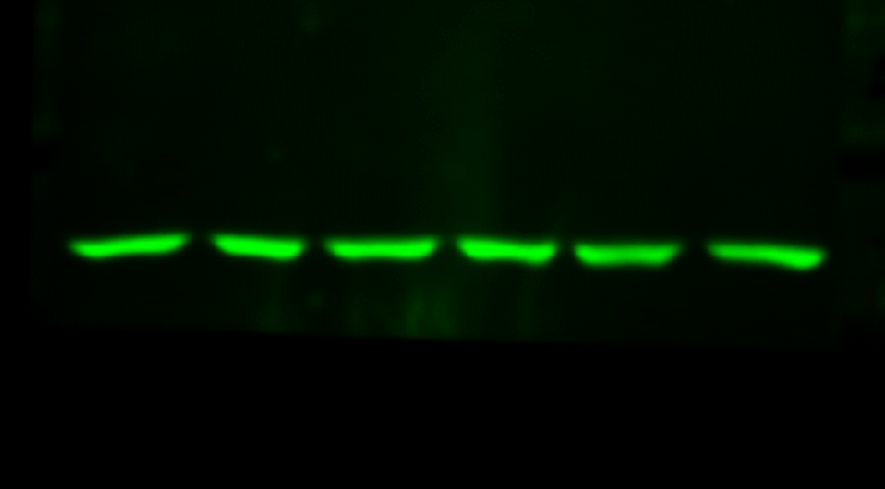

Supplement: Figure 6—source data 1. [file elife-84798-fig6-data1.zip › Figure 6-source data 7/Figure 6-source data 7.tif]

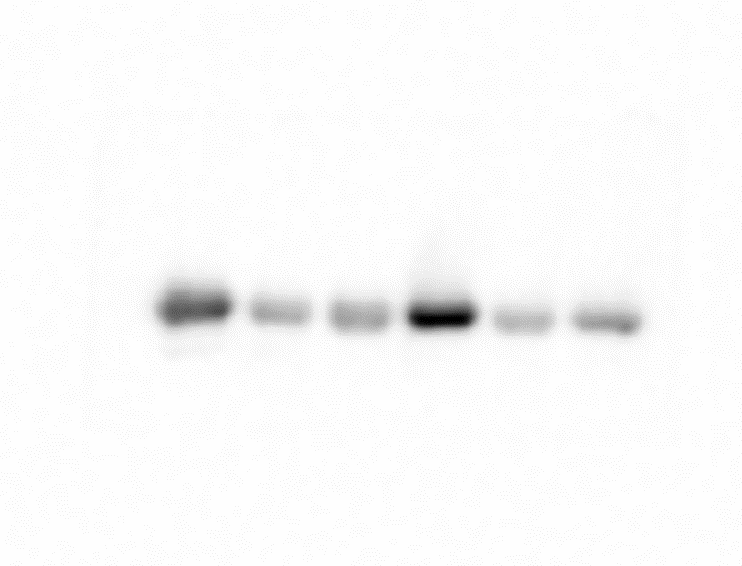

Supplement: Figure 6—source data 1. [file elife-84798-fig6-data1.zip › Figure 6-source data 8/Figure 6-source data 8.tif]

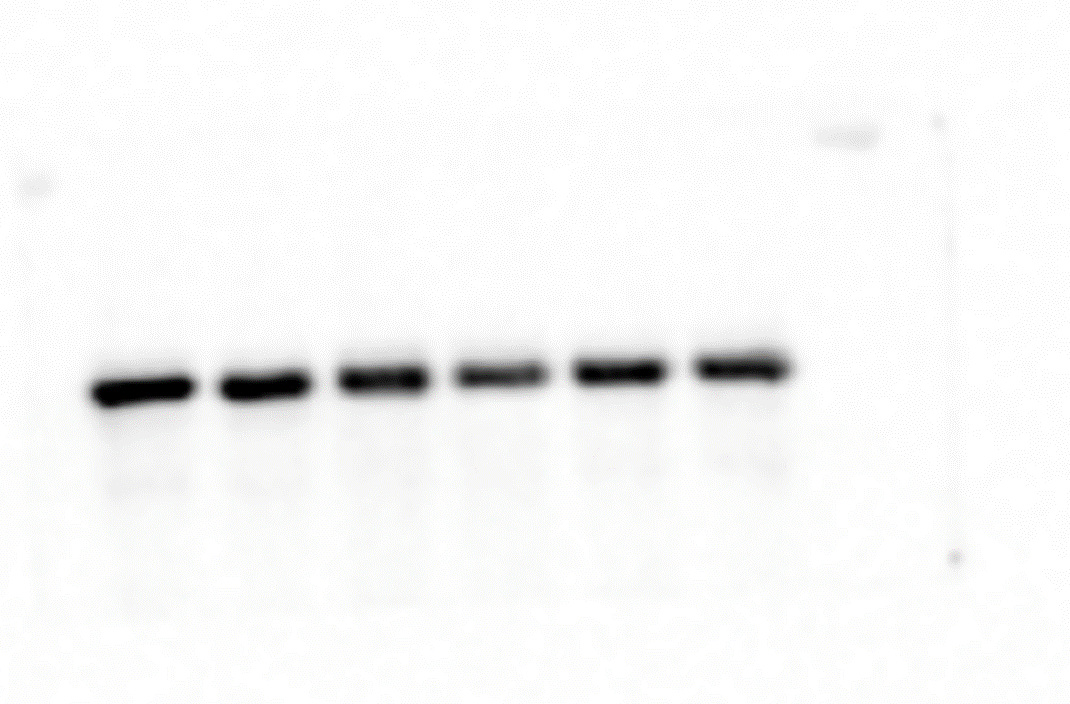

Supplement: Figure 6—source data 1. [file elife-84798-fig6-data1.zip › Figure 6-source data 9/Figure 6-source data 9.tif]

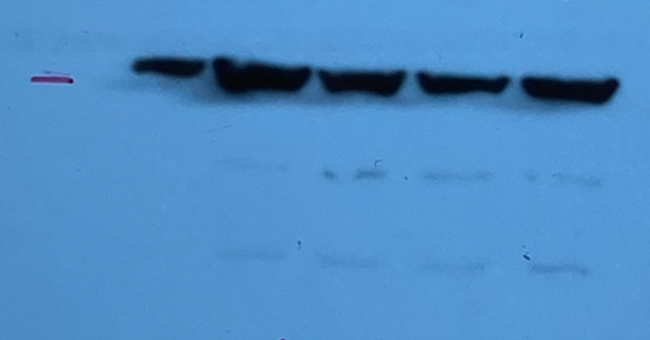

Supplement: Figure 6—source data 1. [file elife-84798-fig6-data1.zip › Figure 6-source data 10/Figure 6-source data 10.tif]

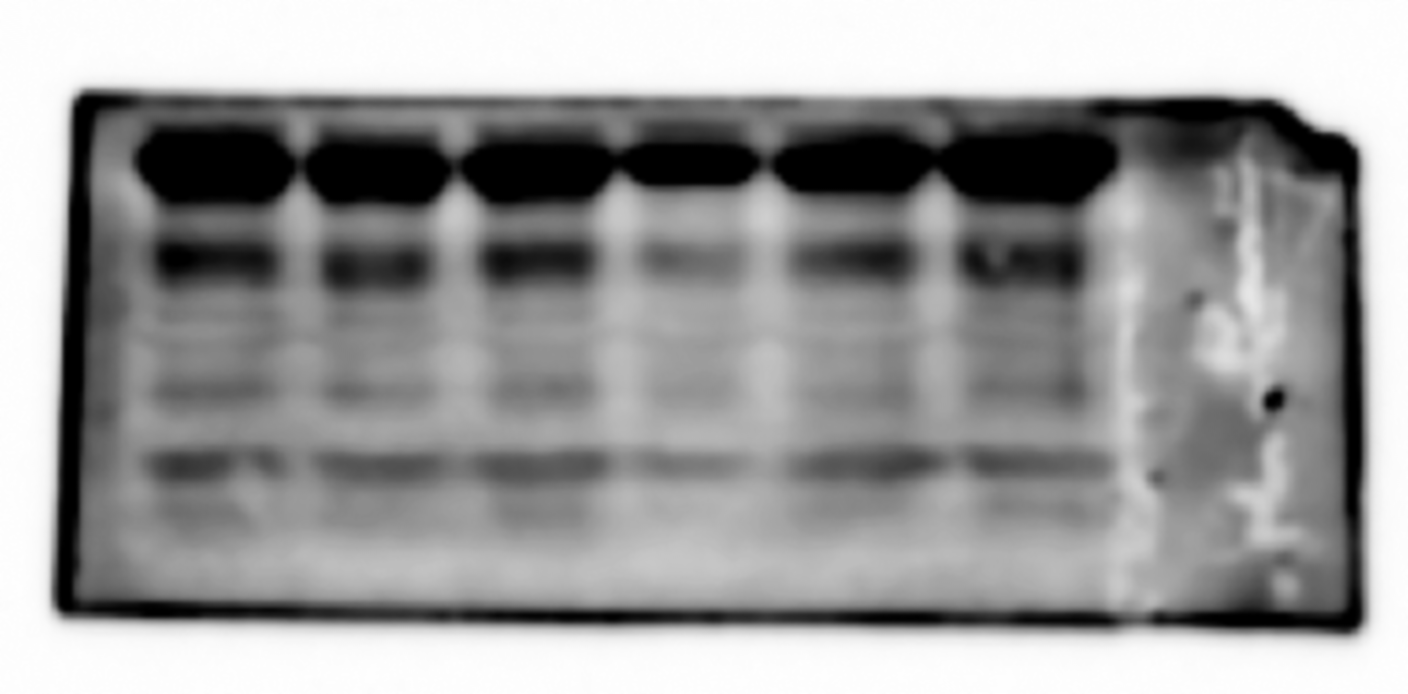

Supplement: Figure 6—source data 1. [file elife-84798-fig6-data1.zip › Figure 6-source data 11/Figure 6-source data 11.tif]

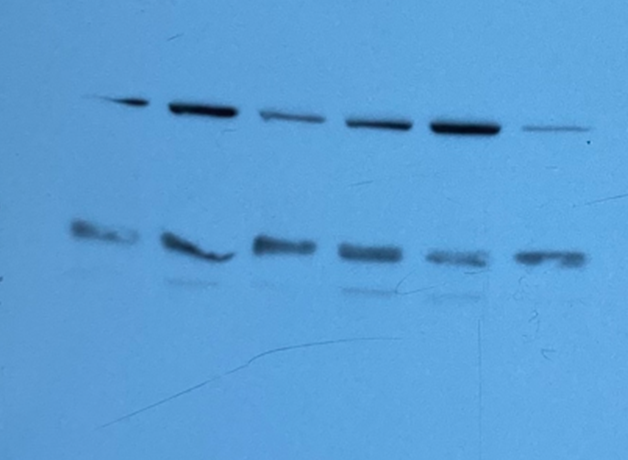

Supplement: Figure 6—source data 1. [file elife-84798-fig6-data1.zip › Figure 6-source data 12/Figure 6-source data 12.tif]

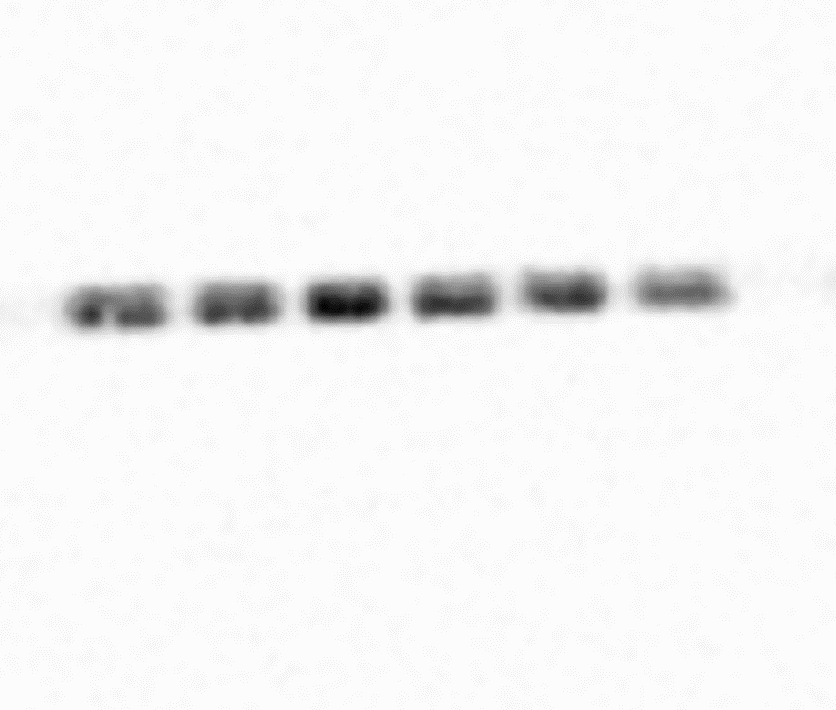

Supplement: Figure 6—source data 1. [file elife-84798-fig6-data1.zip › Figure 6-source data 13/Figure 6-source data 13.tif]

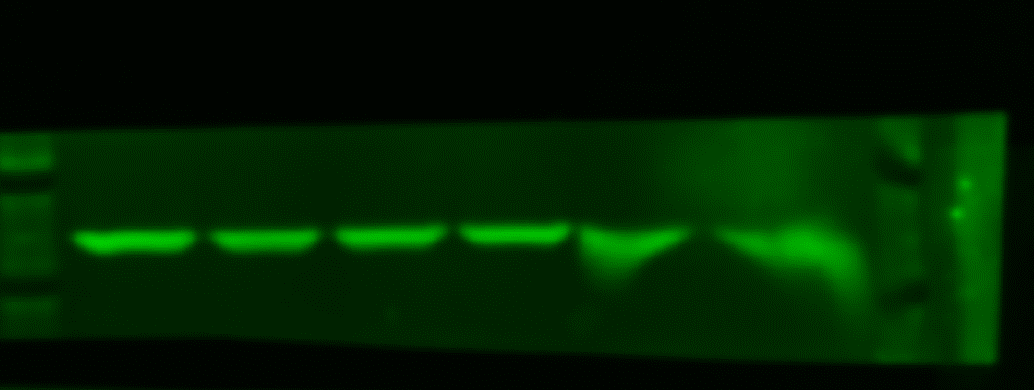

Supplement: Figure 6—source data 1. [file elife-84798-fig6-data1.zip › Figure 6-source data 14/Figure 6-source data 14.tif]

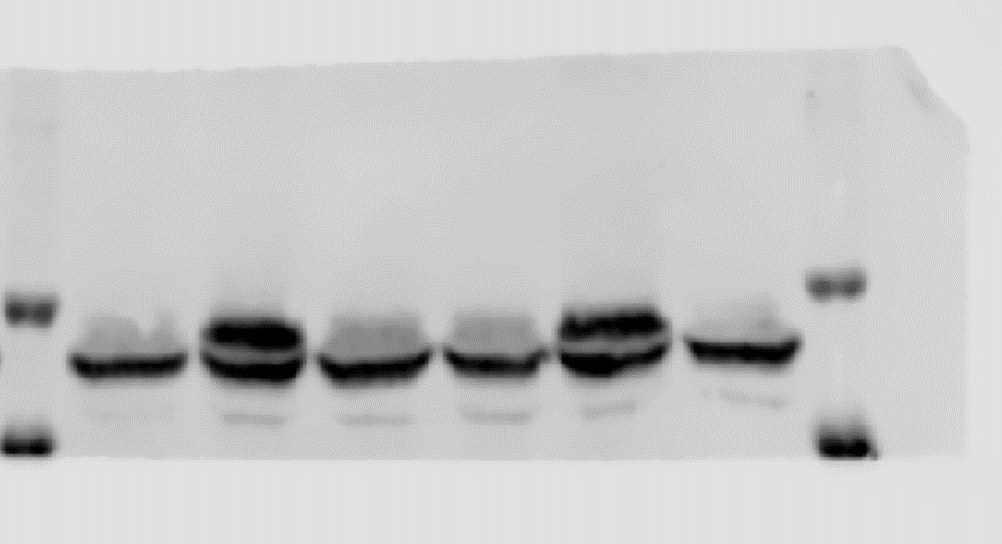

Supplement: Figure 6—source data 1. [file elife-84798-fig6-data1.zip › Figure 6-source data 15/Figure 6-source data 15.tif]
