# Supplementary material for: Hsp47 promotes biogenesis of multi-subunit neuroreceptors in the endoplasmic reticulum
Source: eLife. 2024 Jul 4;13:e84798. doi: 10.7554/eLife.84798 (PMC11257679; doi:10.7554/eLife.84798)
Supplement: Figure 1—source data 2. [file elife-84798-fig1-data2.zip › Figure 1-source data 7/Figure 1-source data 7.pdf]

Figure 1

IB: Hsp47

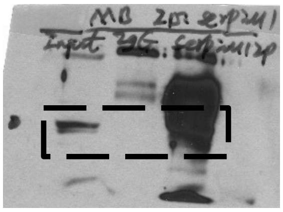

Figure 1A

Top panel

IB: α1

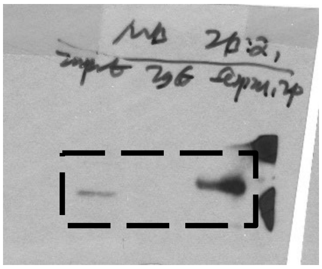

Figure 1A

bottom panel

Figure 1B
Top left panel

IB: α1

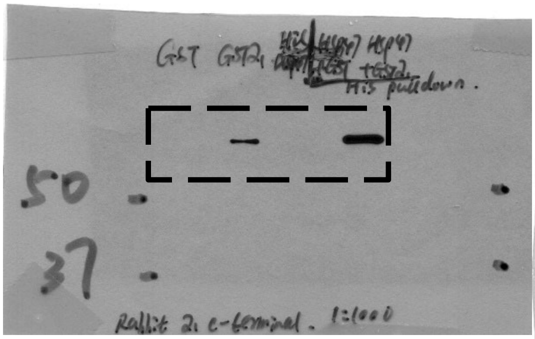

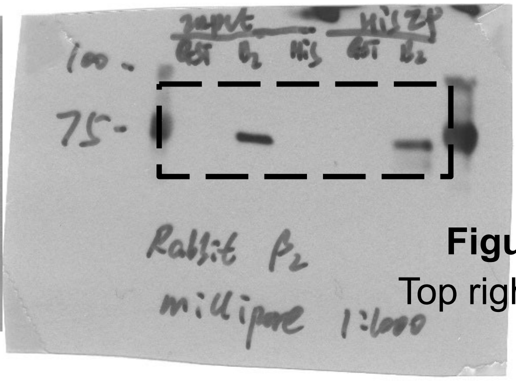

IB: β2

Figure 1B
Top right panel

Figure 1B
Bottom left panel

IB: His

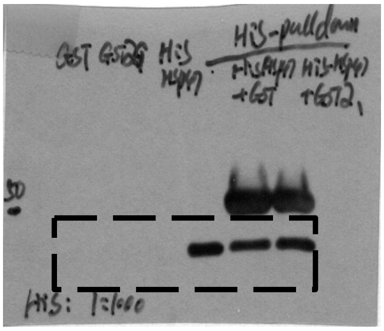

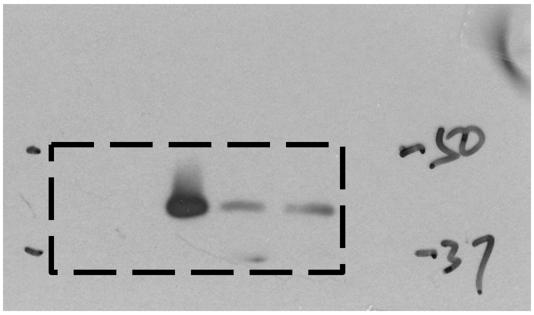

IB: His

Figure 1B
Bottom right panel

Figure 1-source data 1-6
