# Supplementary material for: Hsp47 promotes biogenesis of multi-subunit neuroreceptors in the endoplasmic reticulum
Source: eLife. 2024 Jul 4;13:e84798. doi: 10.7554/eLife.84798 (PMC11257679; doi:10.7554/eLife.84798)
Supplement: Figure 3—source data 2. [file elife-84798-fig3-data2.zip › Figure 3-source data 19/Figure 3-source data 19.pdf]

Figure 3

Figure 3A Top panel

IB: α1

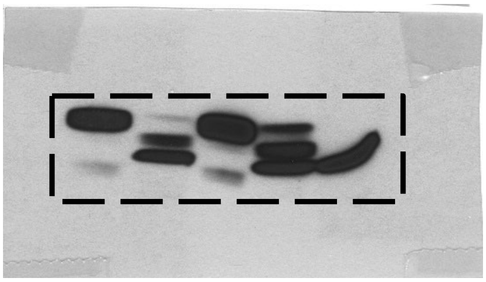

Figure 3A Bottom panel
IB: β-actin

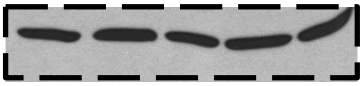

Figure 3B

Top left panel

IB: α1

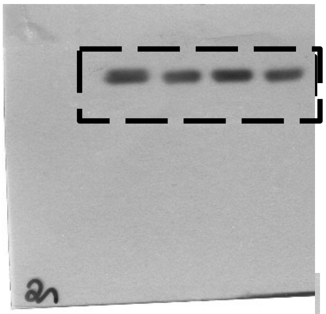

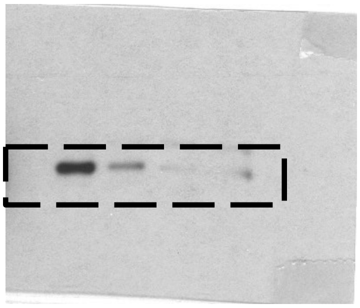

Figure 3B

Top right panel

IB: α1

Figure 3B
Bottom left panel
IB: Hsp47

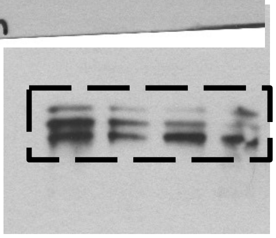

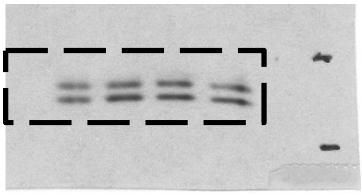

Figure 3B
Bottom right panel
IB: Hsp47

Figure 3C

Top left panel

IB: α1

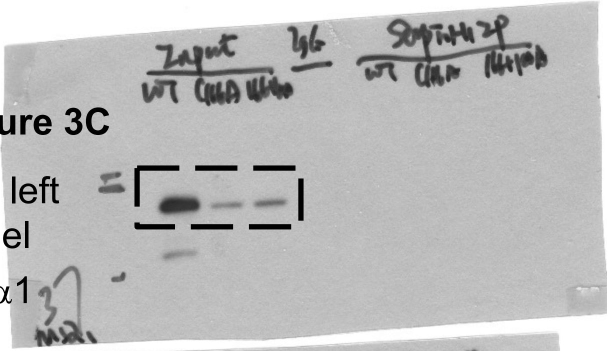

Figure 3C
Bottom left panel

IB: Hsp47

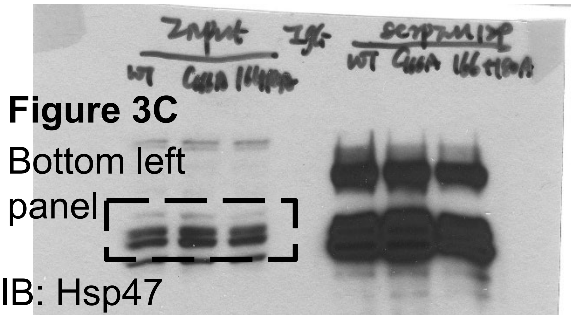

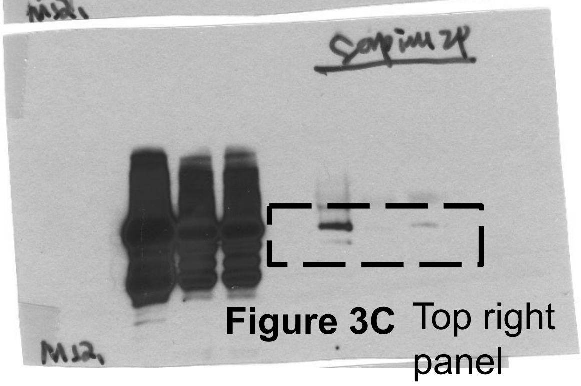

Figure 3C Top right panel

IB: α1

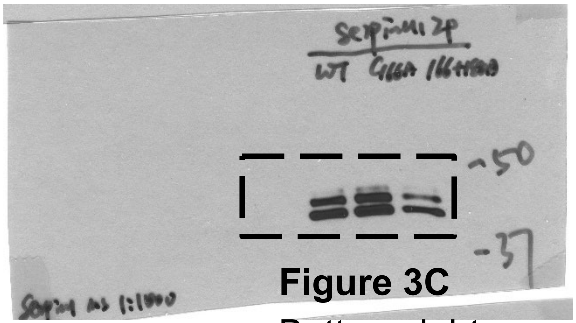

Figure 3C
Bottom right panel IB: Hsp47

Figure 3-source data 1-9

Figure 3

Figure 3D Top left panel IB: α1

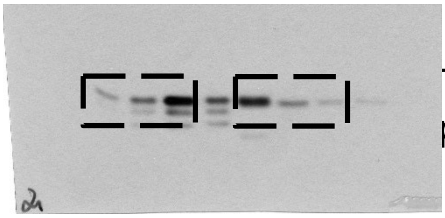

Top right panel IB: α1 Figure 3D

Figure 3D Bottom left panel IB: β-actin

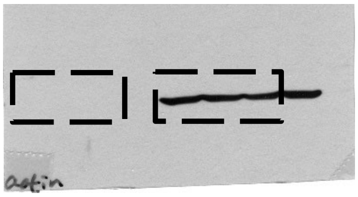

Bottom right panel IB: β-actin Figure 3D

Figure 3E Top left panel IB: BiP

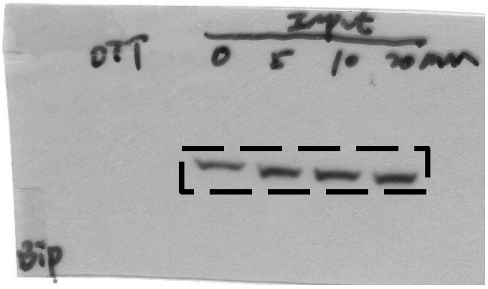

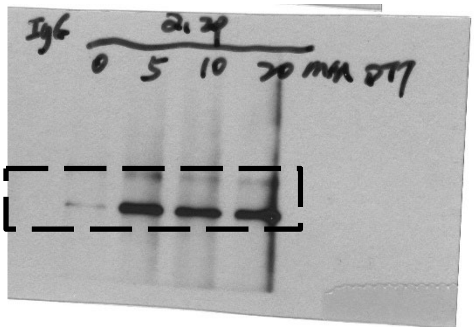

Figure 3E Top right panel IB: BiP

Figure 3E Bottom left panel IB: α1

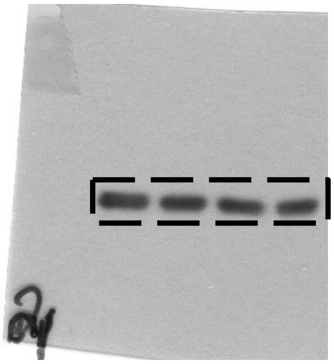

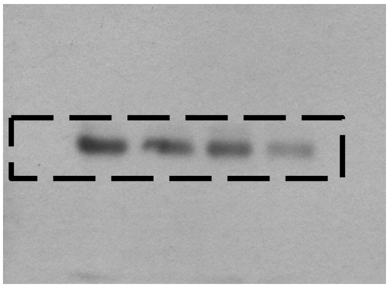

Figure 3E Bottom right panel IB: α1

Figure 3F Top left panel IB: BiP

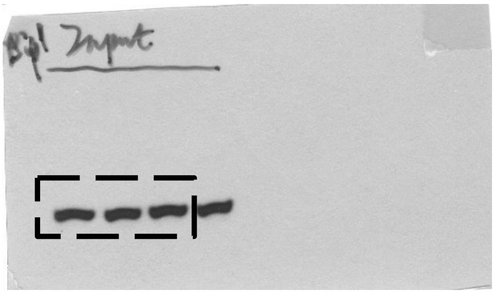

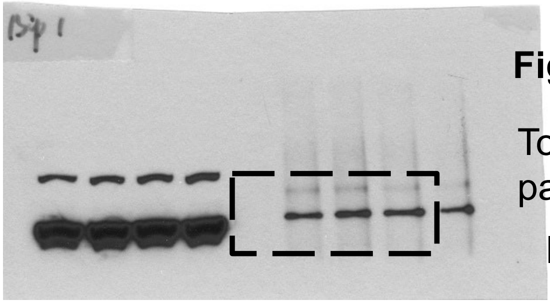

Figure 3F Top right panel IB: BiP

Figure 3F Bottom left panel IB: α1

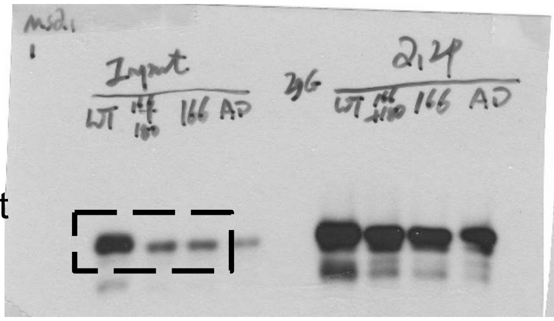

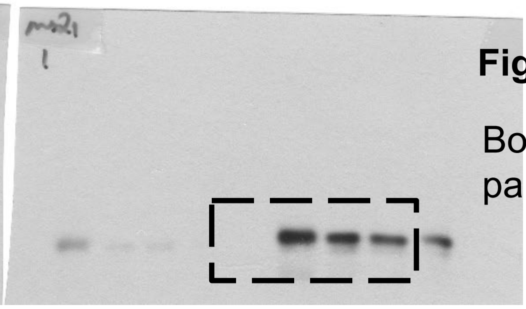

Figure 3F Bottom right panel IB: α1

Figure 3-source data 10-18
