# Supplementary material for: Hsp47 promotes biogenesis of multi-subunit neuroreceptors in the endoplasmic reticulum
Source: eLife. 2024 Jul 4;13:e84798. doi: 10.7554/eLife.84798 (PMC11257679; doi:10.7554/eLife.84798)
Supplement: Figure 3—figure supplement 1—source data 2. [file elife-84798-fig3-figsupp1-data2.zip › Figure 3-figure supplement 1-source data 10/Figure 3-figure supplement 1-source data 10.pdf]

Figure 3—figure supplement 1

Figure 3—figure supplement 1B, top panel

IB: ubiquitin

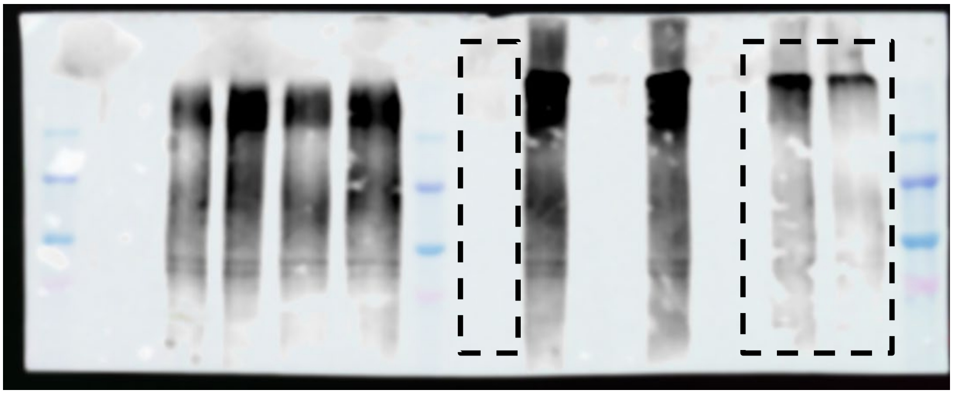

Figure 3—figure supplement 1B, bottom panel

IB: α1

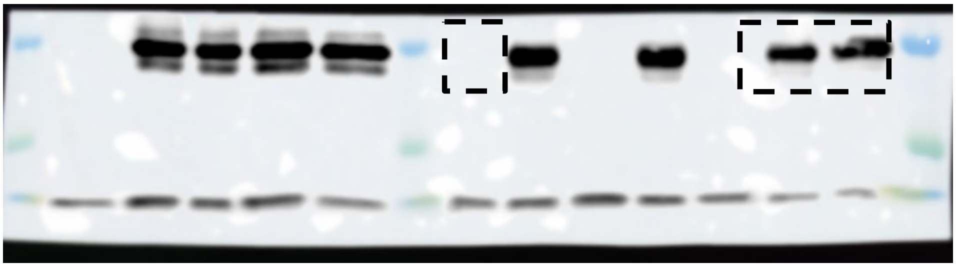

Figure 3—figure supplement 1C

IB: α1

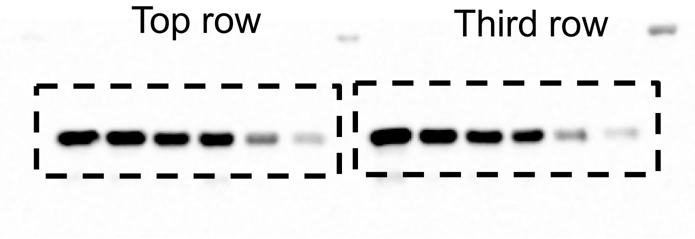

Second row

Bottom row

Figure 3—figure supplement 1C

IB: β-actin

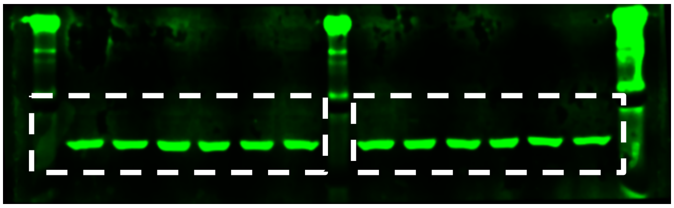

Figure 3—figure supplement 1-source data 1-4

Figure 3—figure supplement 1

Figure 3—figure supplement 1D
IB: α1

Top row   Third row

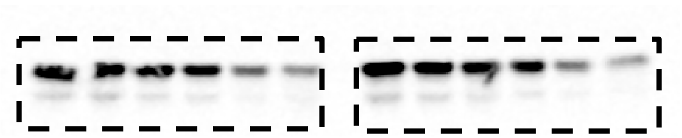

Figure 3—figure supplement 1D
IB: β-actin

Bottom row

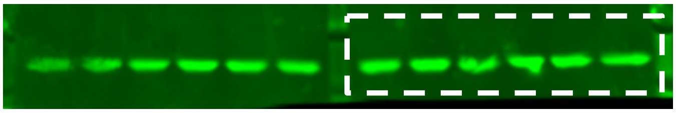

Figure 3—figure supplement 1D
IB: β-actin

Second row

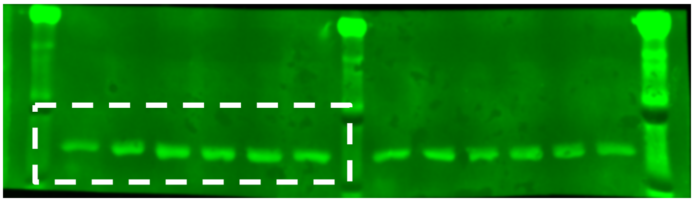

Figure 3—figure supplement 1E, top panel
IB: BiP

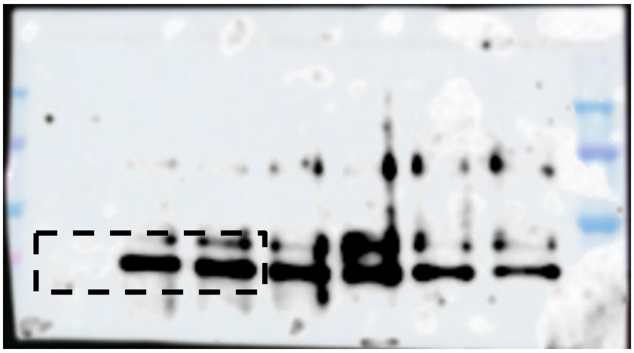

Figure 3—figure supplement 1E, bottom panel
IB: α1

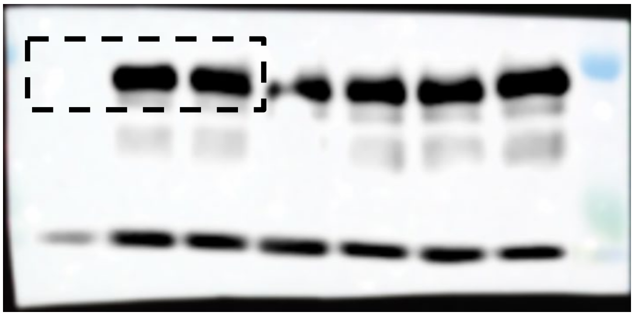

Figure 3—figure supplement 1-source data 5-9
