# Supplementary material for: Hsp47 promotes biogenesis of multi-subunit neuroreceptors in the endoplasmic reticulum
Source: eLife. 2024 Jul 4;13:e84798. doi: 10.7554/eLife.84798 (PMC11257679; doi:10.7554/eLife.84798)
Supplement: Figure 4—source data 2. [file elife-84798-fig4-data2.zip › Figure 4-source data 19/Figure 4-source data 19.pdf]

Figure 4B

IB: α1

Figure 4B
Top row
Left panel

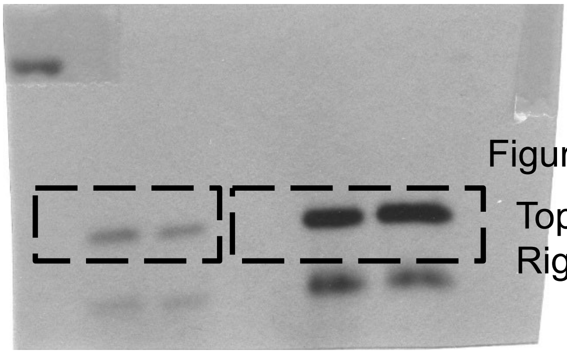

Figure 4B
Top row
Right panel

IB: FLAG

Figure 4B
Second row
Left panel

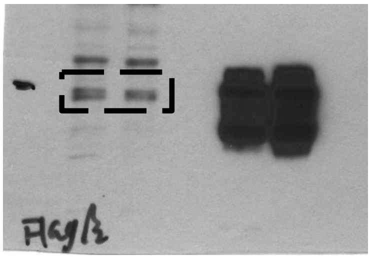

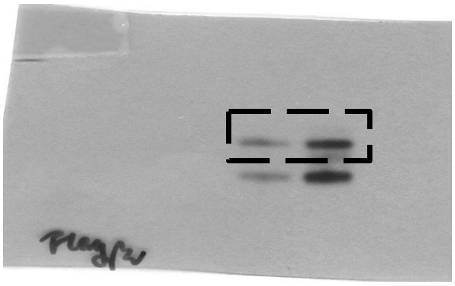

IB: FLAG

Figure 4B
Second row
Right panel

IB: Hsp47
Figure 4B
Bottom row

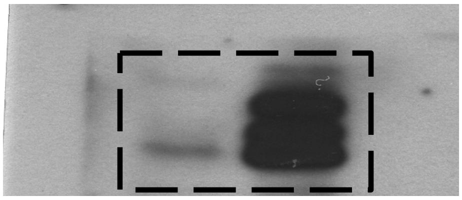

Figure 4-source data 1-4

Figure 4C

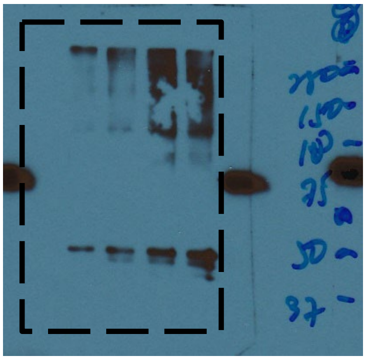

IB: α1 Figure 4C Row 1 Column 1

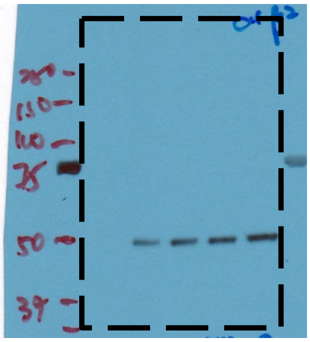

Figure 4C Row 1 Column 2

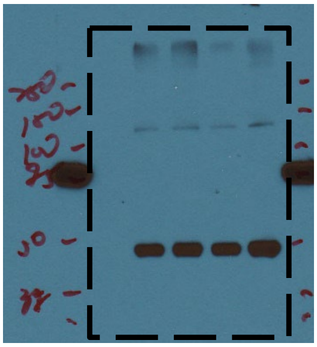

Figure 4C Row 1 Column 3

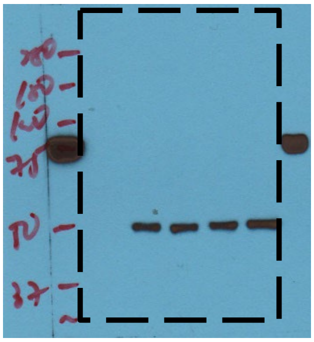

Figure 4C Row 1 Column 4

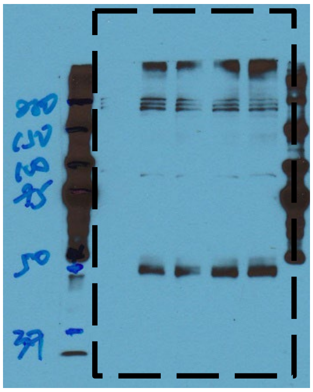

IB: β2 Figure 4C Row 2 Column 1

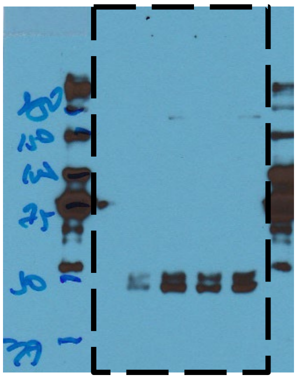

Figure 4C Row 2 Column 2

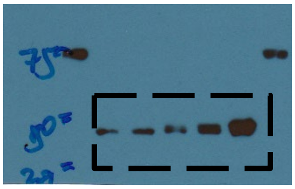

IB: Hsp47 Figure 4C Row 3 Column 1

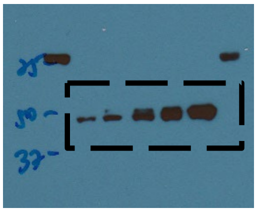

Figure 4C Row 3 Column 2

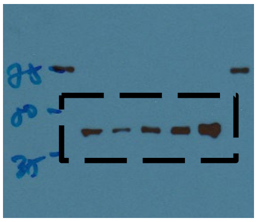

Figure 4C Row 3 Column 3

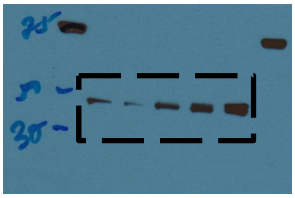

Figure 4C Row 3 Column 4

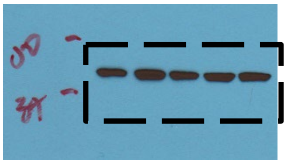

IB: β-actin Figure 4C Row 4 Column 1

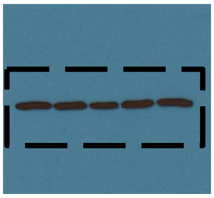

Figure 4C Row 4 Column 2

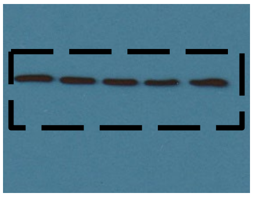

Figure 4C Row 4 Column 3

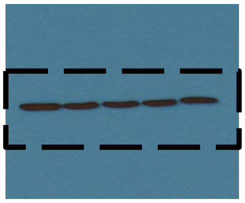

Figure 4C Row 4 Column 4

Figure 4-source data 5-18
