# Supplementary material for: Hsp47 promotes biogenesis of multi-subunit neuroreceptors in the endoplasmic reticulum
Source: eLife. 2024 Jul 4;13:e84798. doi: 10.7554/eLife.84798 (PMC11257679; doi:10.7554/eLife.84798)
Supplement: Figure 5—source data 2. [file elife-84798-fig5-data2.zip › Figure 5-source data 17/Figure 5-source data 17.pdf]

Figure 5

Figure 5A
Top panel

IB: $\alpha 1$

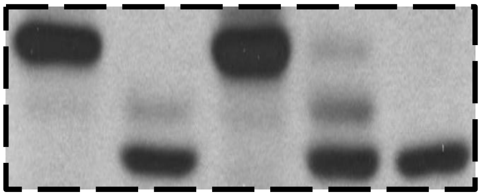

Figure 5A
Middle panel

IB: Hsp47

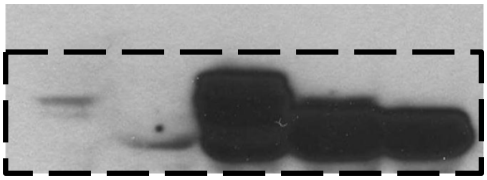

Figure 5A
Bottom panel

IB: β-actin

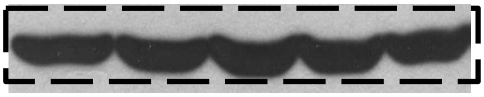

Figure 5B,
top panel

IB: ubiquitin

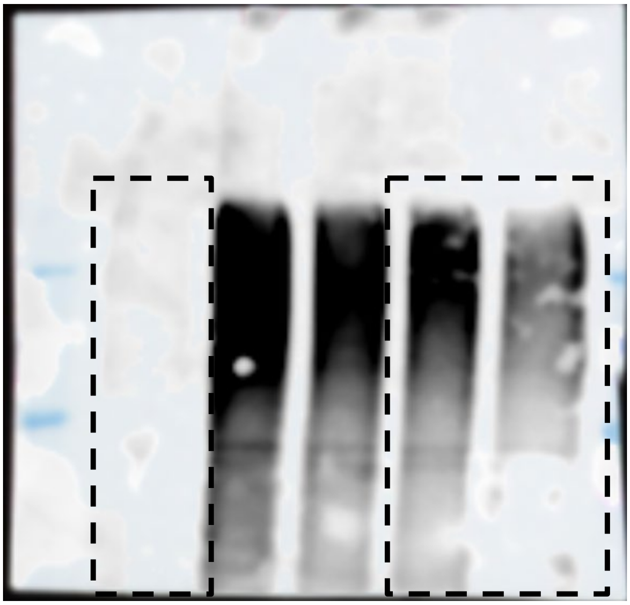

Figure 5B,
bottom panel

IB: $\alpha 1$

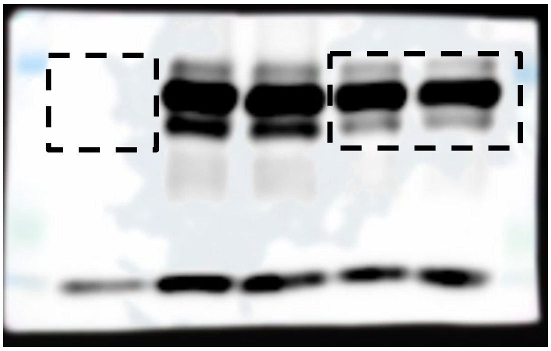

Figure 5-source data 1-5

Figure 5

Figure 5C, top row

IB: α1

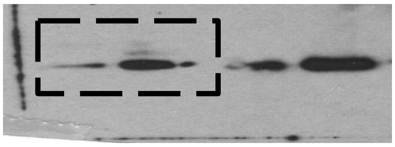

Figure 5C, second row

IB: ATPase

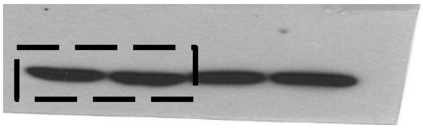

Figure 5C, third row

IB: α1

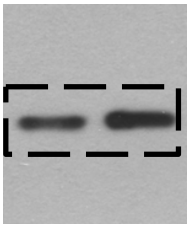

Figure 5C, fourth row

IB: Hsp47

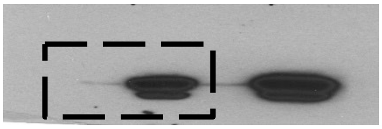

Figure 5C, bottom row

IB: β-actin

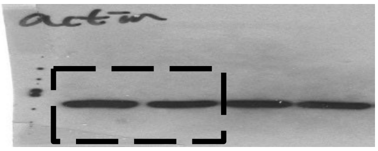

Figure 5F
first row

IB: α1

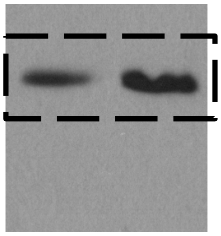

Figure 5F
second row

IB: ATPase

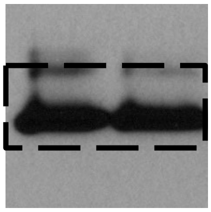

Figure 5F
third row

IB: α1

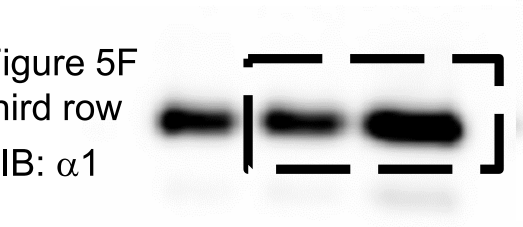

Figure 5F
fourth row

IB: ATPase

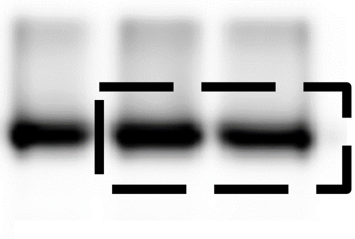

Figure 5F
fifth row

IB: α1

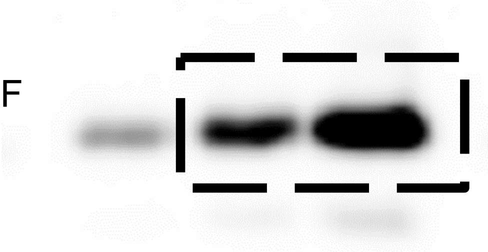

Figure 5F
sixth row

IB: ATPase

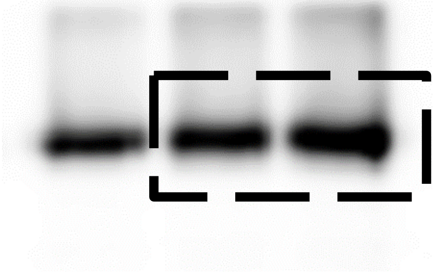

Figure 5-source data 6-16
