# Supplementary material for: Hsp47 promotes biogenesis of multi-subunit neuroreceptors in the endoplasmic reticulum
Source: eLife. 2024 Jul 4;13:e84798. doi: 10.7554/eLife.84798 (PMC11257679; doi:10.7554/eLife.84798)
Supplement: Figure 5—figure supplement 1—source data 2. [file elife-84798-fig5-figsupp1-data2.zip › Figure 5-figure supplement 1-source data 8/Figure 5-figure supplement 1-source data 8.pdf]

Figure 5—figure supplement 1

Figure 5—figure supplement 1A

IB: α1

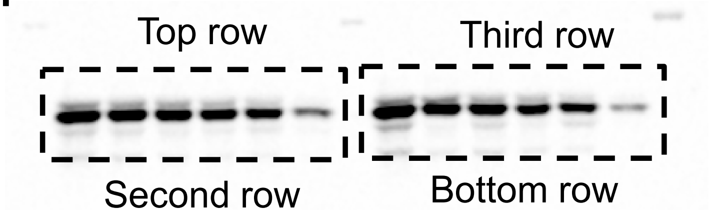

Figure 5—figure supplement 1A

IB: β-actin

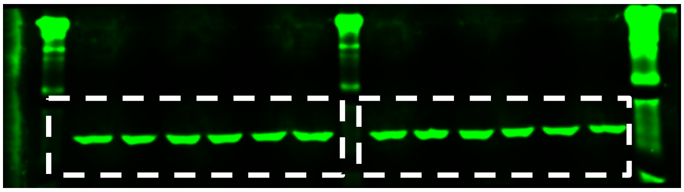

Figure 5—figure supplement 1B

IB: α1

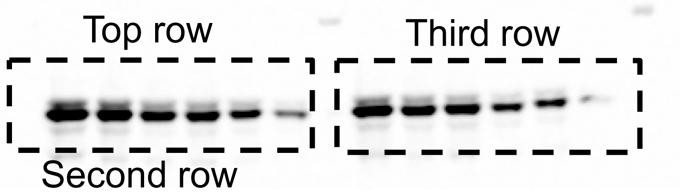

Figure 5—figure supplement 1B

IB: β-actin

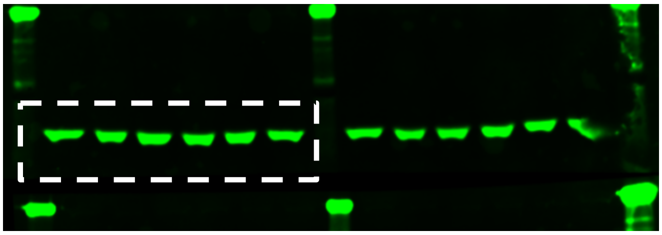

Figure 5—figure supplement 1B

IB: β-actin

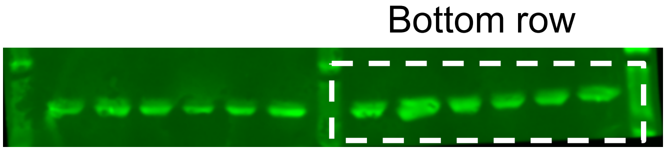

Figure 5—figure supplement 1C, top panel

IB: BiP

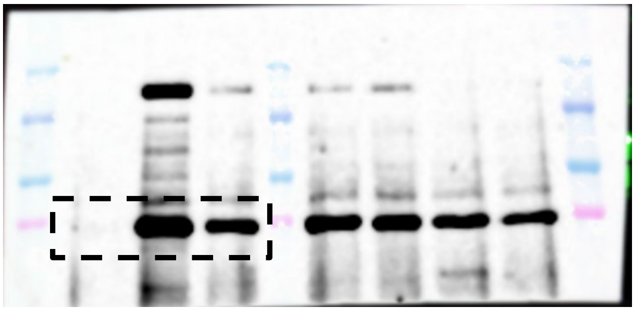

Figure 5—figure supplement 1C, bottom panel

IB: α1

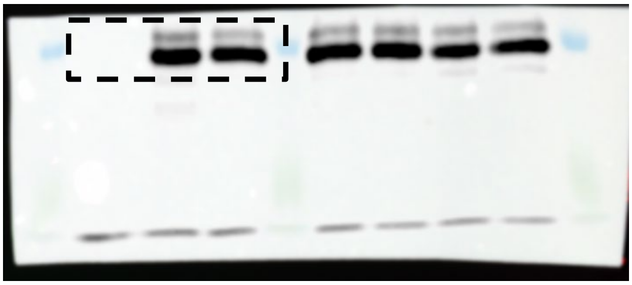

Figure 5—figure supplement 1-source data 1-7
