# Supplementary figures and images for: Hsp47 promotes biogenesis of multi-subunit neuroreceptors in the endoplasmic reticulum (part 2 of 2)
Source: eLife. 2024 Jul 4;13:e84798. doi: 10.7554/eLife.84798 (PMC11257679; doi:10.7554/eLife.84798)

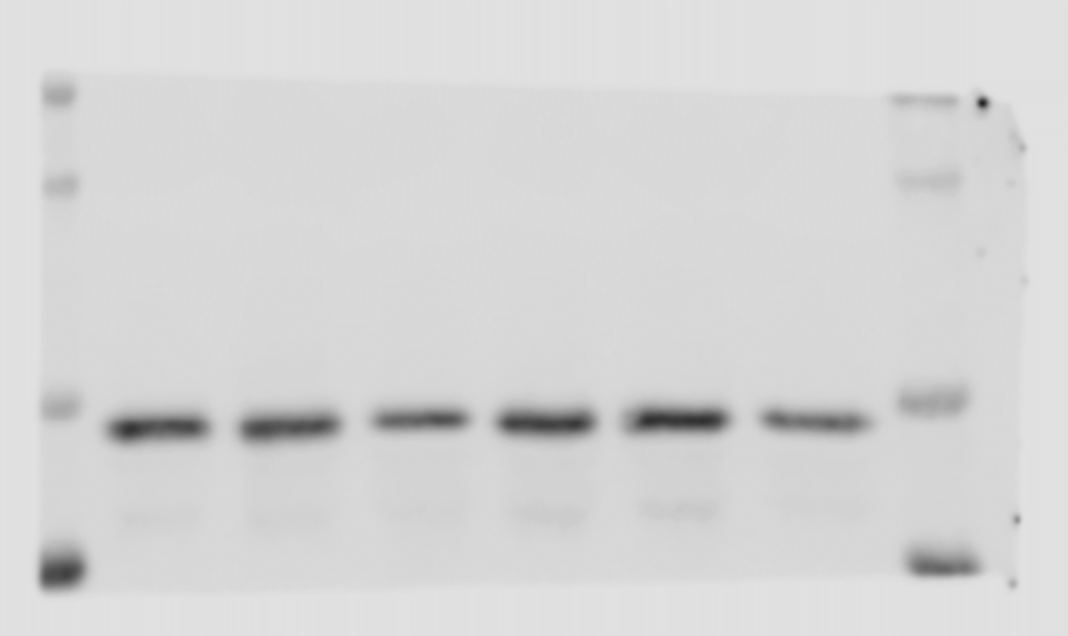

Supplement: Figure 6—source data 1. [file elife-84798-fig6-data1.zip › Figure 6-source data 16/Figure 6-source data 16.tif]

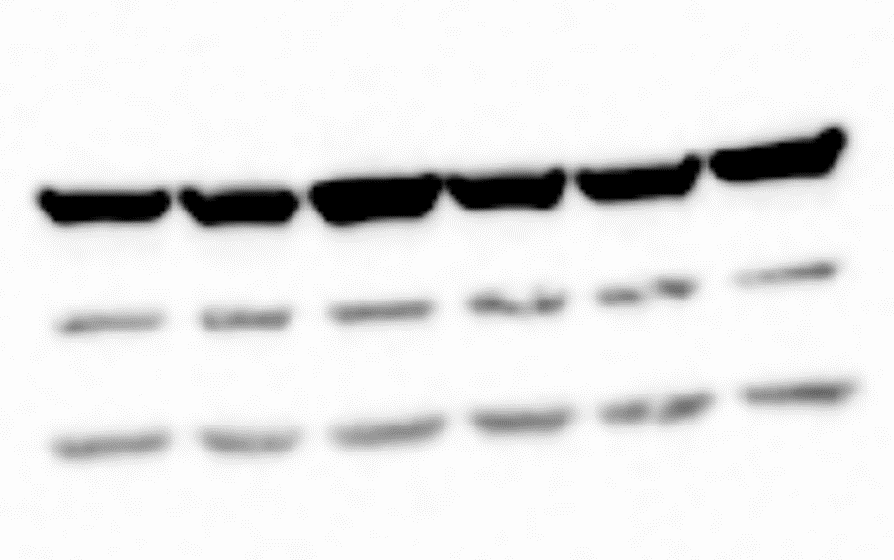

Supplement: Figure 6—source data 1. [file elife-84798-fig6-data1.zip › Figure 6-source data 17/Figure 6-source data 17.tif]

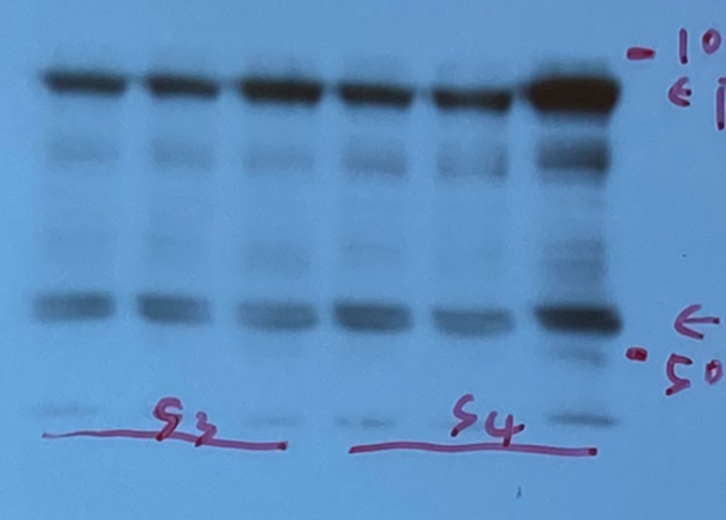

Supplement: Figure 6—source data 1. [file elife-84798-fig6-data1.zip › Figure 6-source data 18/Figure 6-source data 18.tif]

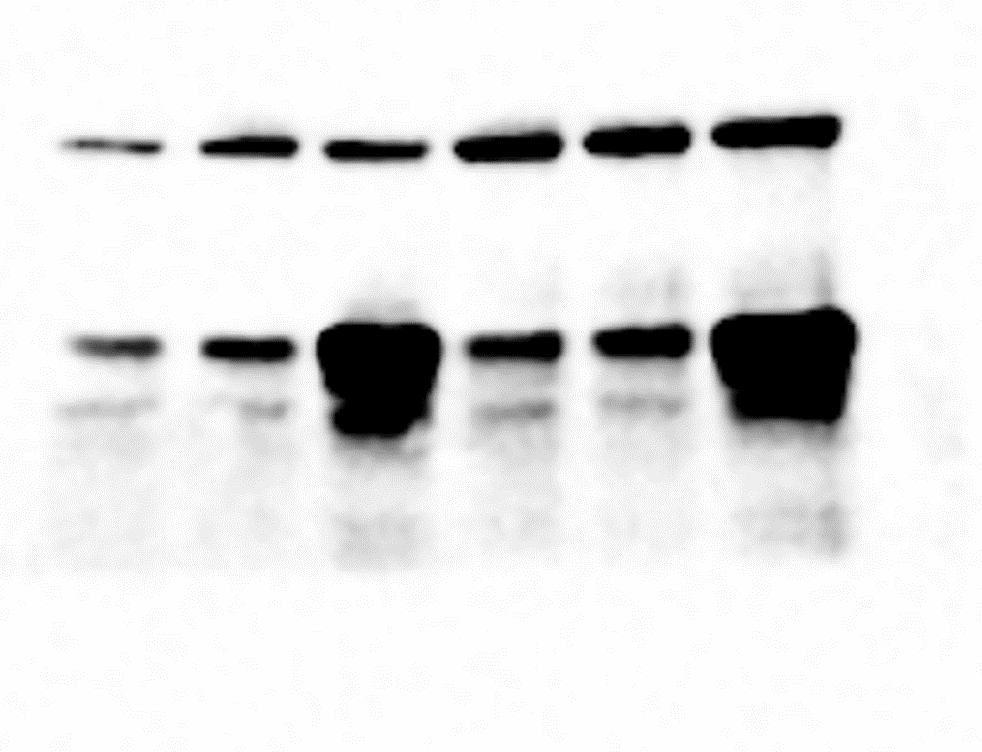

Supplement: Figure 6—source data 1. [file elife-84798-fig6-data1.zip › Figure 6-source data 19/Figure 6-source data 19.tif]

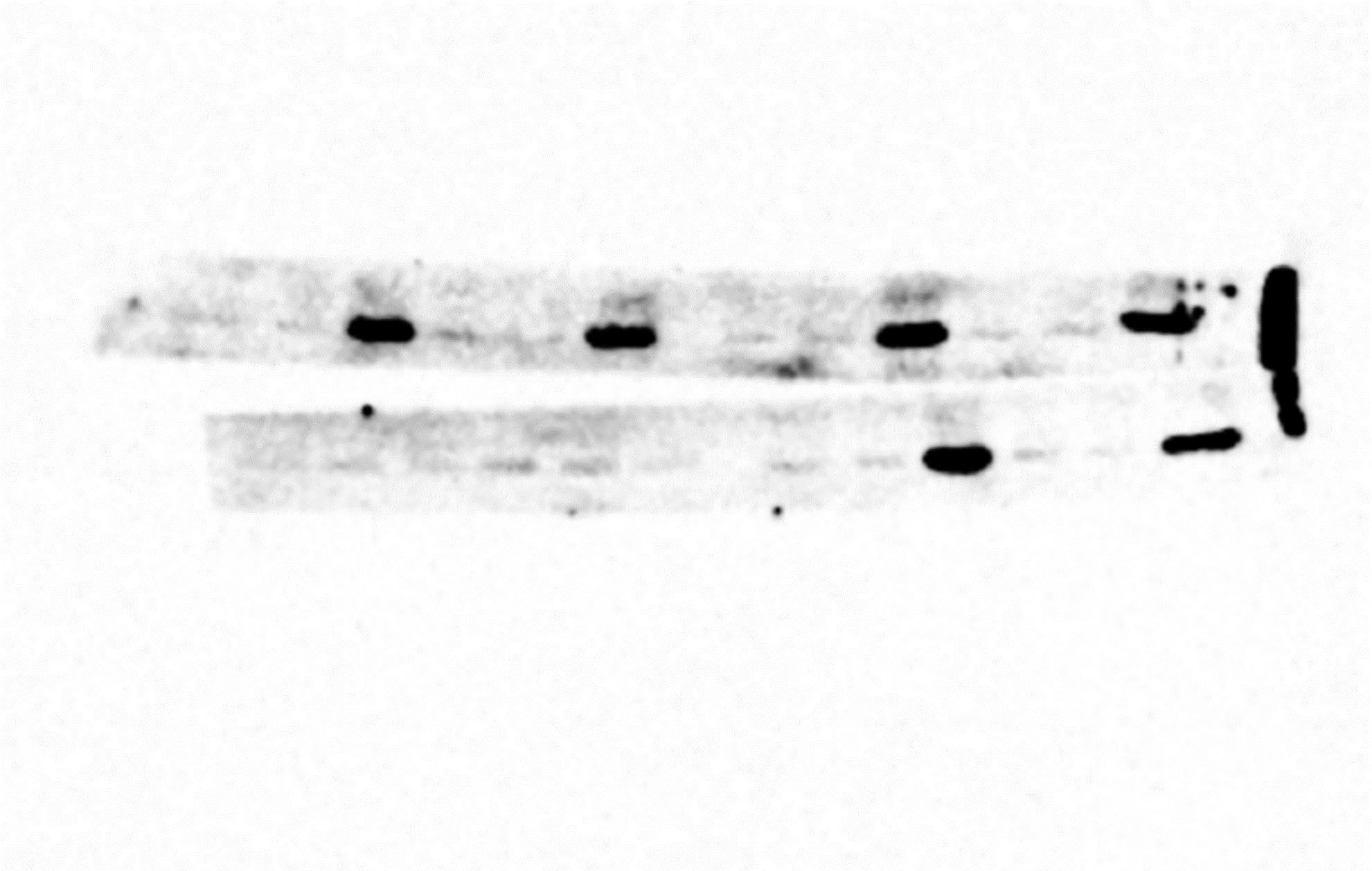

Supplement: Figure 6—source data 1. [file elife-84798-fig6-data1.zip › Figure 6-source data 20/Figure 6-source data 20.tif]

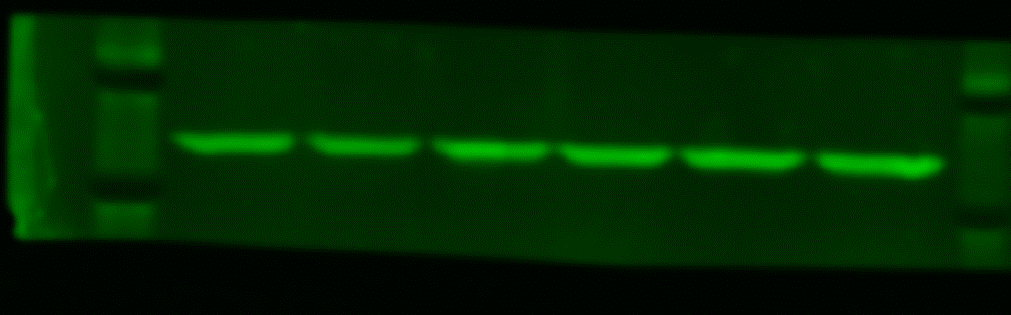

Supplement: Figure 6—source data 1. [file elife-84798-fig6-data1.zip › Figure 6-source data 21/Figure 6-source data 21.tif]

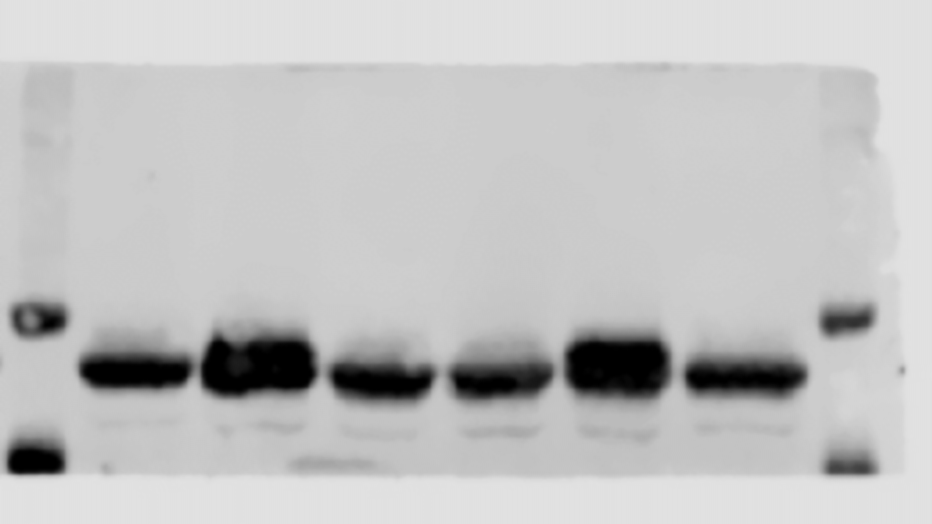

Supplement: Figure 6—source data 1. [file elife-84798-fig6-data1.zip › Figure 6-source data 22/Figure 6-source data 22.tif]

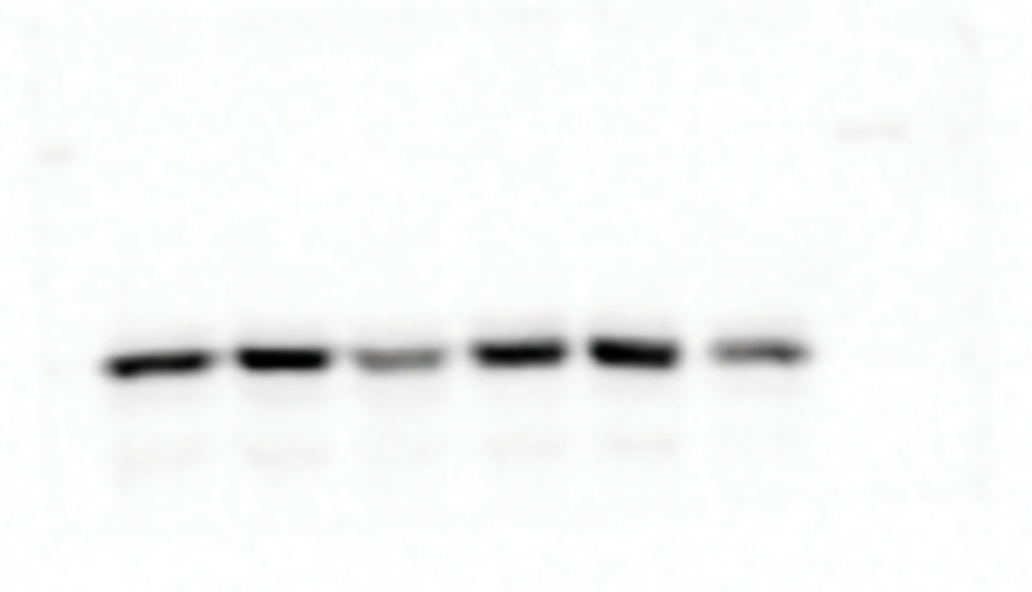

Supplement: Figure 6—source data 1. [file elife-84798-fig6-data1.zip › Figure 6-source data 23/Figure 6-source data 23.tif]

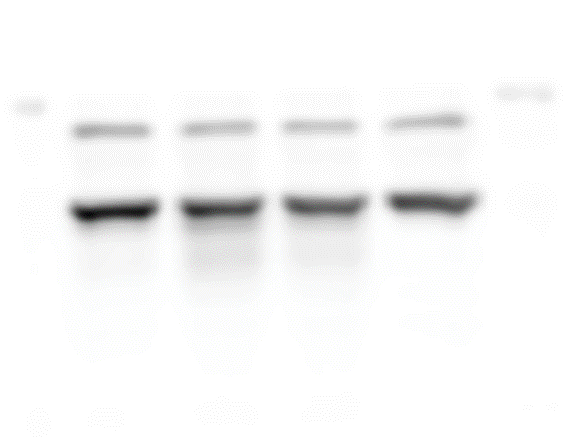

Supplement: Figure 6—source data 1. [file elife-84798-fig6-data1.zip › Figure 6-source data 24/Figure 6-source data 24.tif]

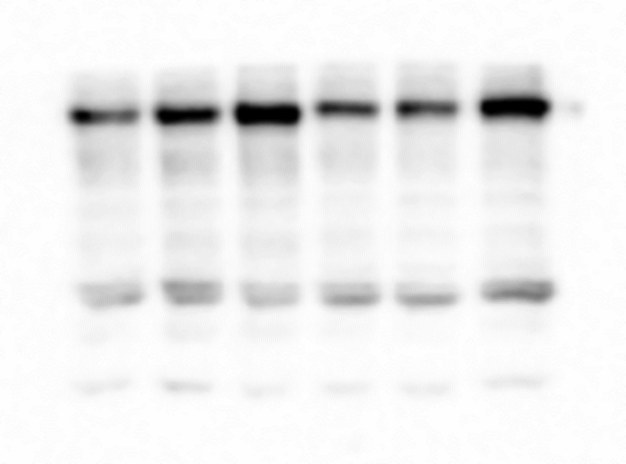

Supplement: Figure 6—source data 1. [file elife-84798-fig6-data1.zip › Figure 6-source data 25/Figure 6-source data 25.tif]

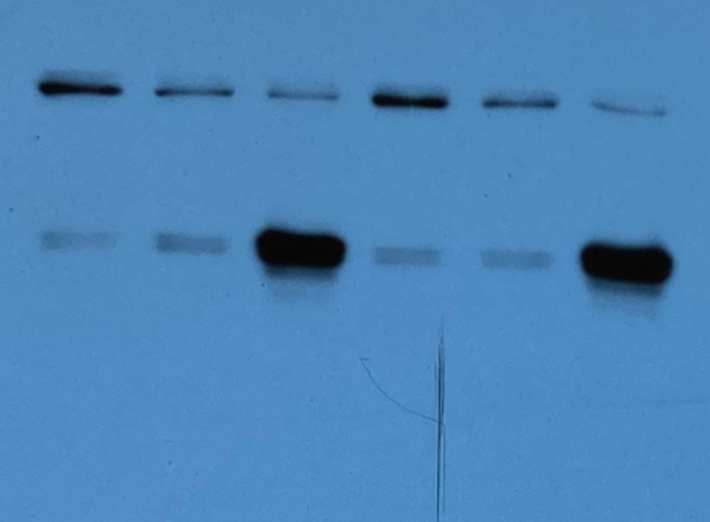

Supplement: Figure 6—source data 1. [file elife-84798-fig6-data1.zip › Figure 6-source data 26/Figure 6-source data 26.tif]

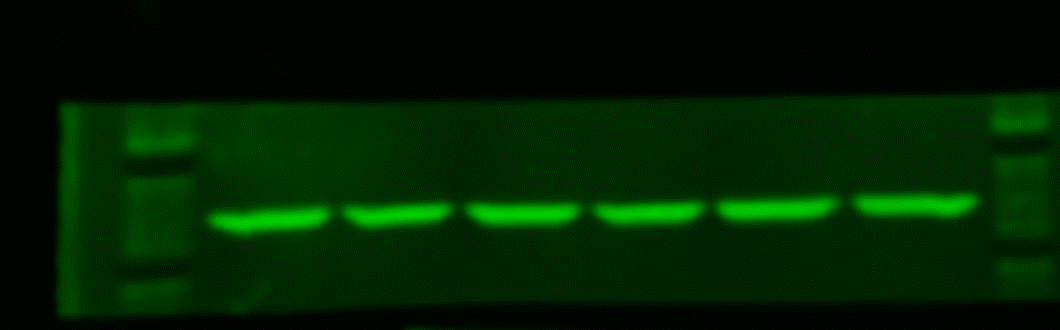

Supplement: Figure 6—source data 1. [file elife-84798-fig6-data1.zip › Figure 6-source data 28/Figure 6-source data 28.tif]

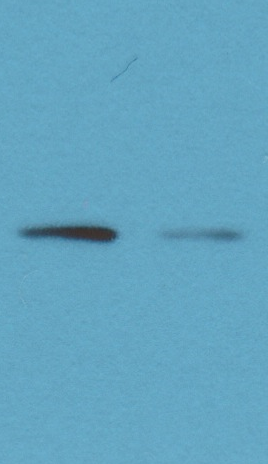

Supplement: Figure 6—source data 1. [file elife-84798-fig6-data1.zip › Figure 6-source data 29/Figure 6-source data 29.tif]

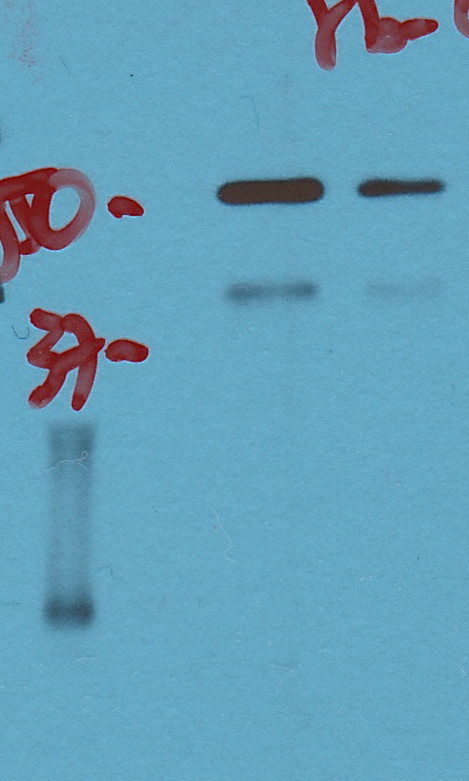

Supplement: Figure 6—source data 1. [file elife-84798-fig6-data1.zip › Figure 6-source data 30/Figure 6-source data 30.tif]

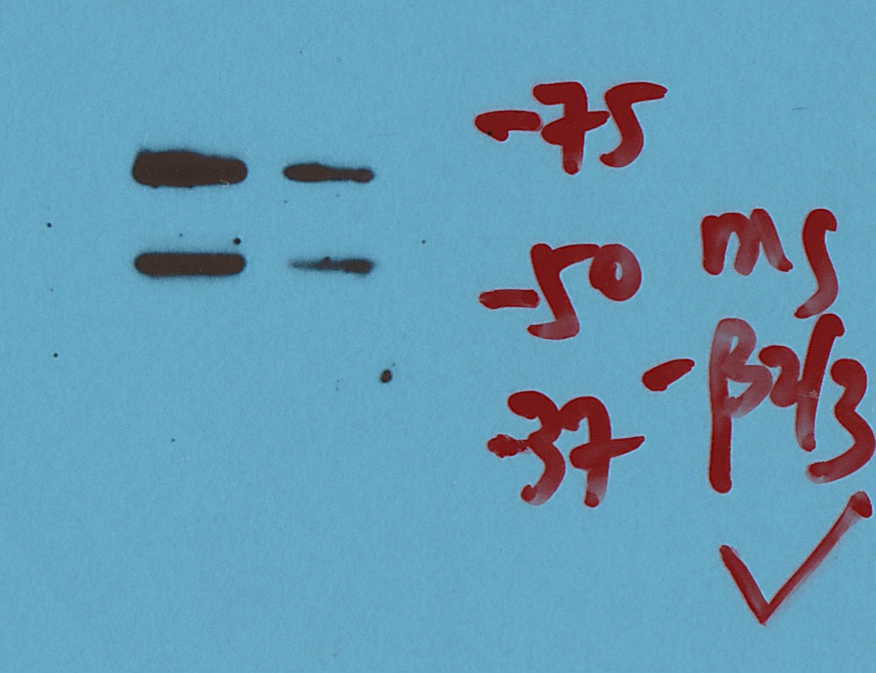

Supplement: Figure 6—source data 1. [file elife-84798-fig6-data1.zip › Figure 6-source data 31/Figure 6-source data 31.tif]

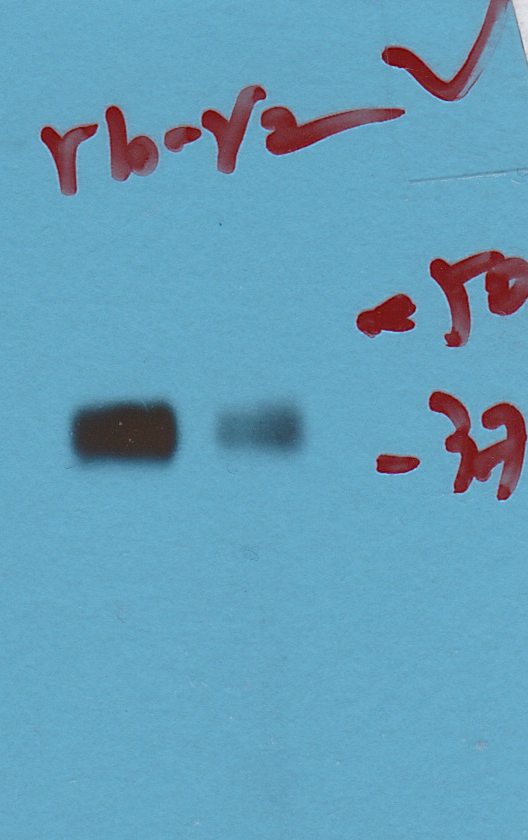

Supplement: Figure 6—source data 1. [file elife-84798-fig6-data1.zip › Figure 6-source data 32/Figure 6-source data 32.tif]

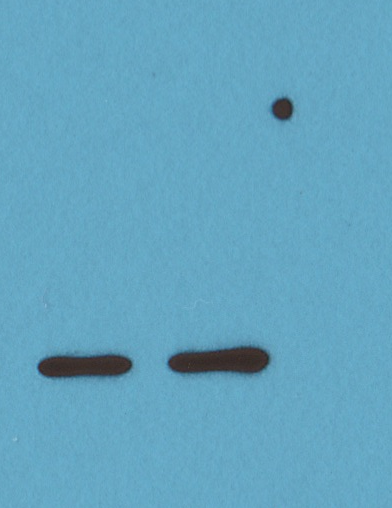

Supplement: Figure 6—source data 1. [file elife-84798-fig6-data1.zip › Figure 6-source data 33/Figure 6-source data 33.tif]

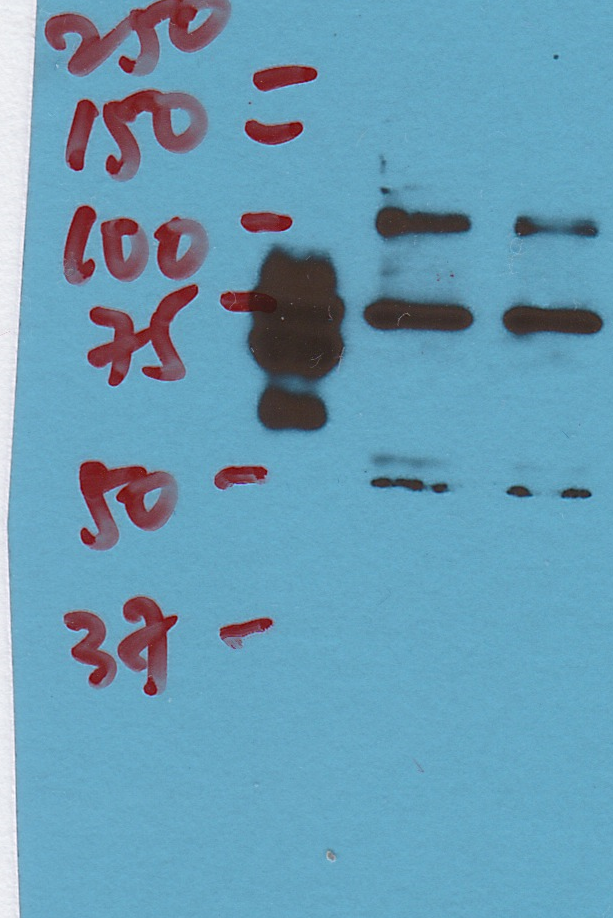

Supplement: Figure 6—source data 1. [file elife-84798-fig6-data1.zip › Figure 6-source data 34/Figure 6-source data 34.tif]

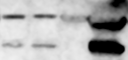

Supplement: Figure 6—source data 1. [file elife-84798-fig6-data1.zip › Figure 6-source data 35/Figure 6-source data 35.tif]

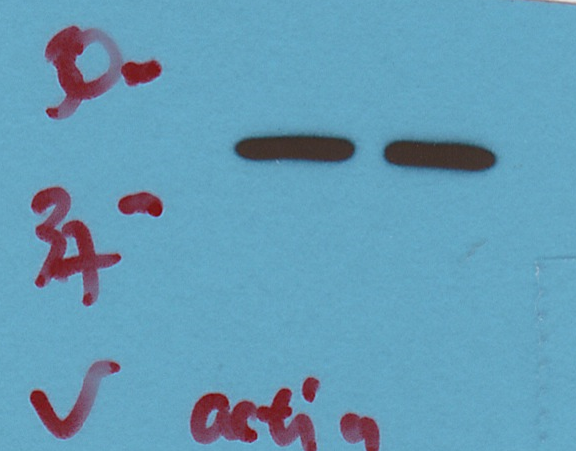

Supplement: Figure 6—source data 1. [file elife-84798-fig6-data1.zip › Figure 6-source data 36/Figure 6-source data 36.tif]

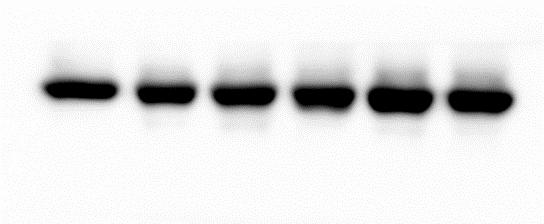

Supplement: Figure 6—source data 1. [file elife-84798-fig6-data1.zip › Figure 6-source data 37/Figure 6-source data 37.tif]

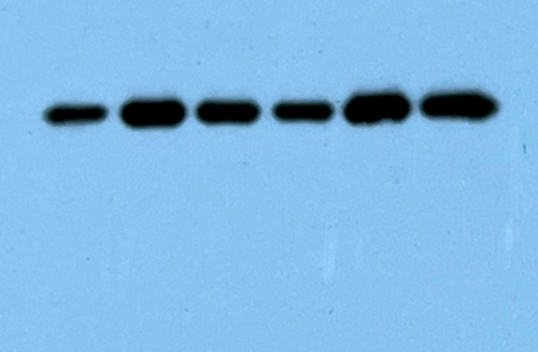

Supplement: Figure 6—source data 1. [file elife-84798-fig6-data1.zip › Figure 6-source data 38/Figure 6-source data 38.tif]

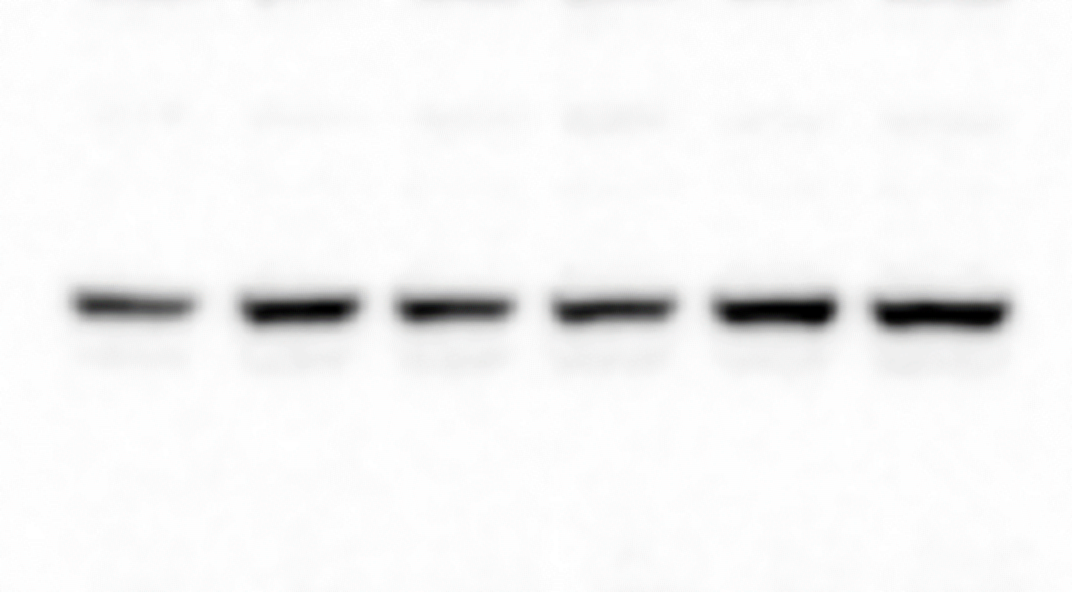

Supplement: Figure 6—source data 1. [file elife-84798-fig6-data1.zip › Figure 6-source data 39/Figure 6-source data 39.tif]

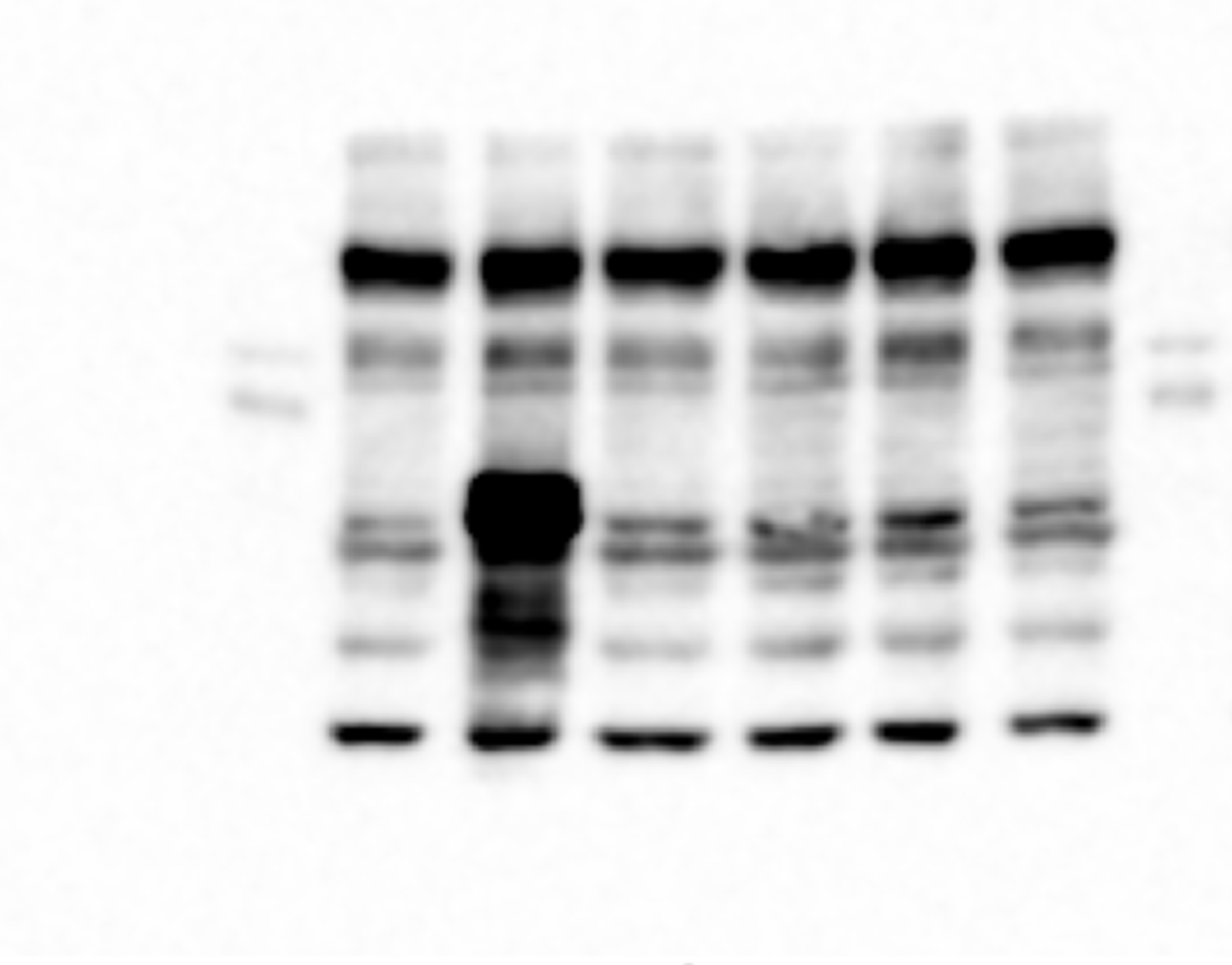

Supplement: Figure 6—source data 1. [file elife-84798-fig6-data1.zip › Figure 6-source data 40/Figure 6-source data 40.tif]

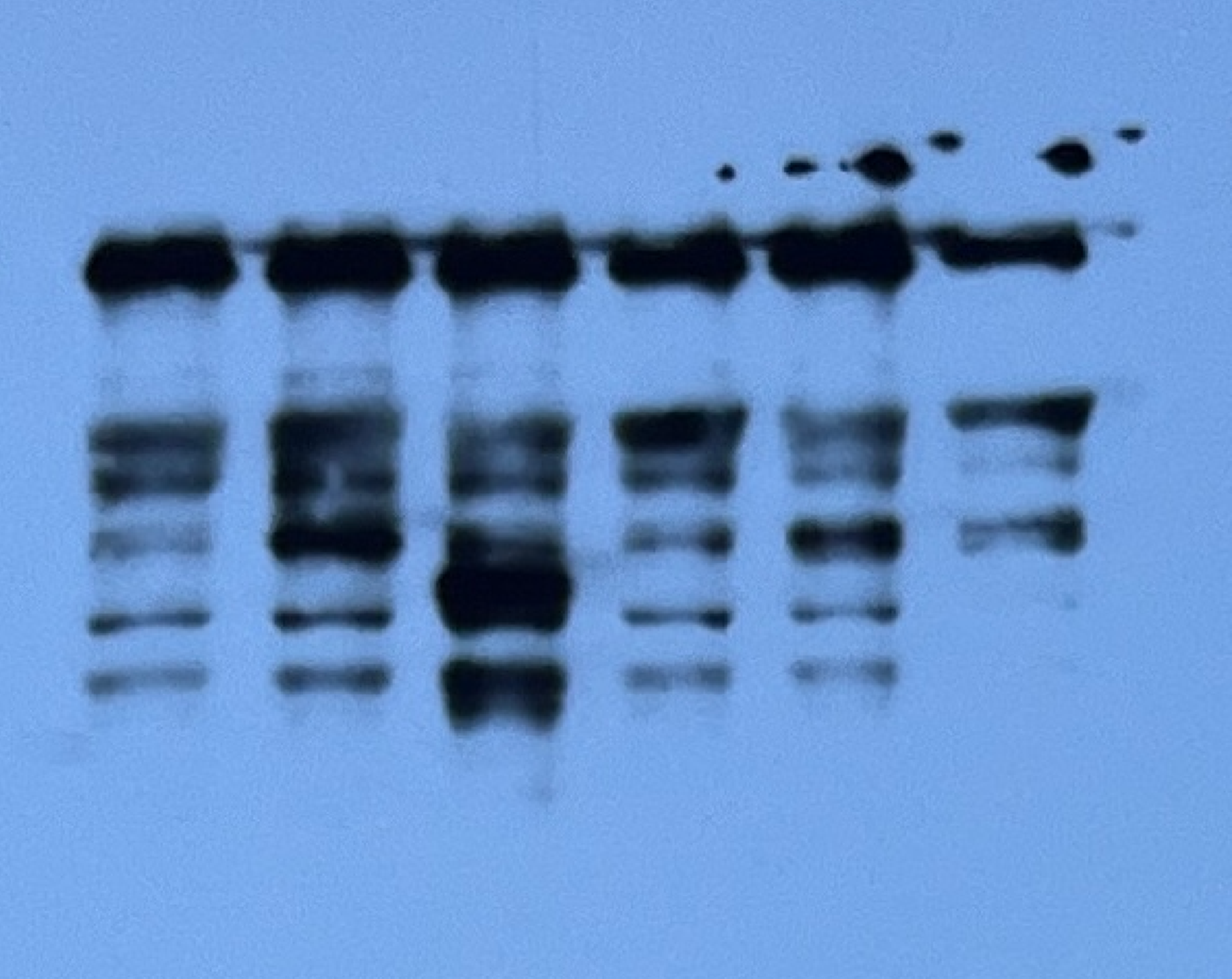

Supplement: Figure 6—source data 1. [file elife-84798-fig6-data1.zip › Figure 6-source data 41/Figure 6-source data 41.tif]

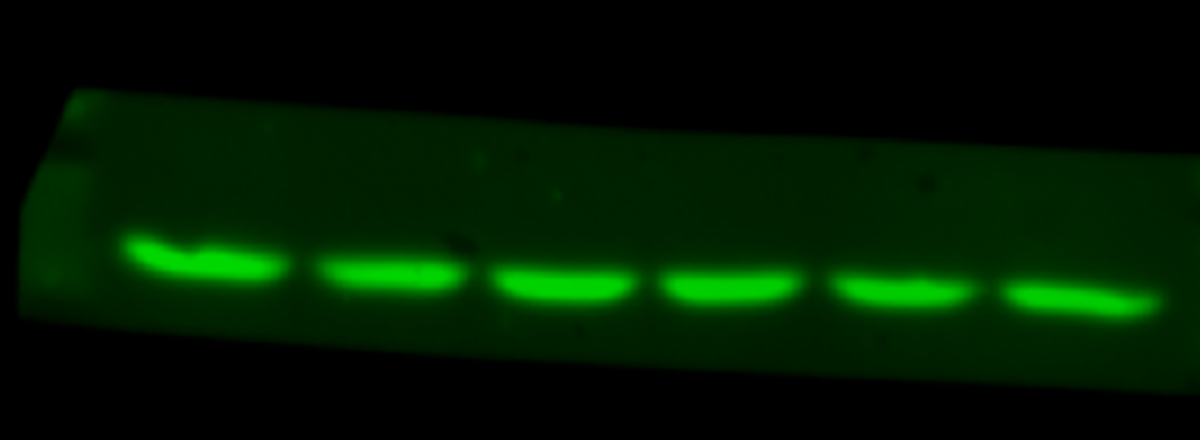

Supplement: Figure 6—source data 1. [file elife-84798-fig6-data1.zip › Figure 6-source data 42/Figure 6-source data 42.tif]

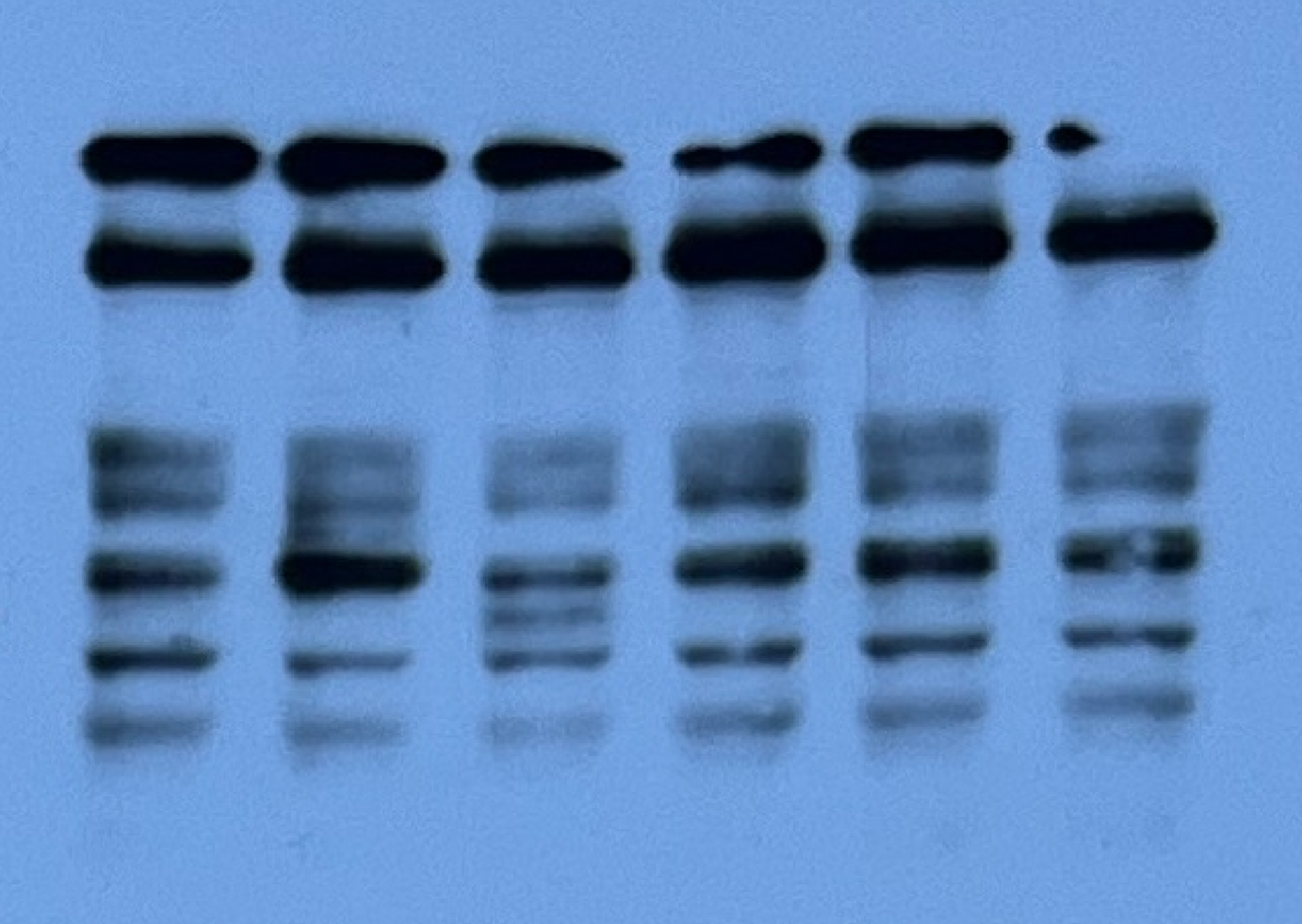

Supplement: Figure 6—source data 1. [file elife-84798-fig6-data1.zip › Figure 6-source data 47/Figure 6-source data 47.tif]

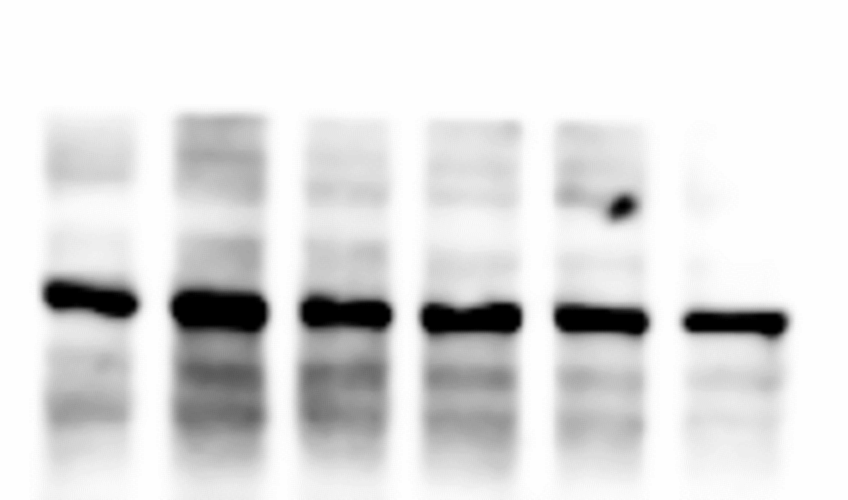

Supplement: Figure 7—source data 1. [file elife-84798-fig7-data1.zip › Figure 7-source data 1/Figure 7-source data 1.tif]

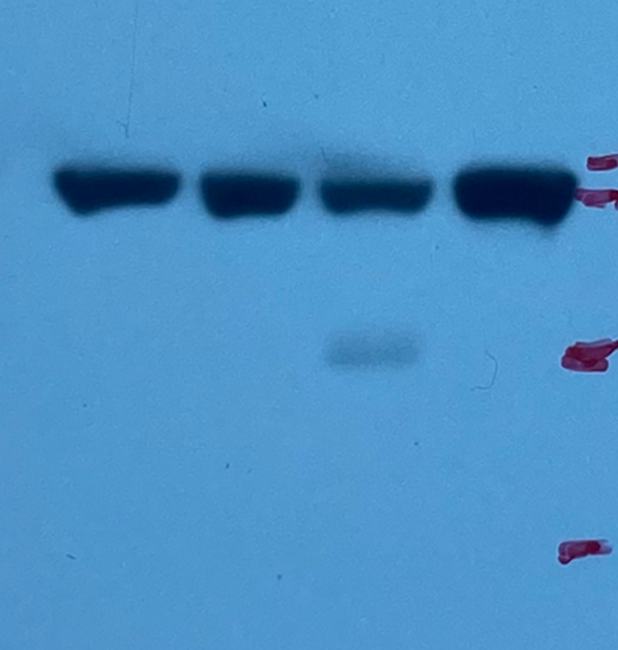

Supplement: Figure 7—source data 1. [file elife-84798-fig7-data1.zip › Figure 7-source data 2/Figure 7-source data 2.tif]

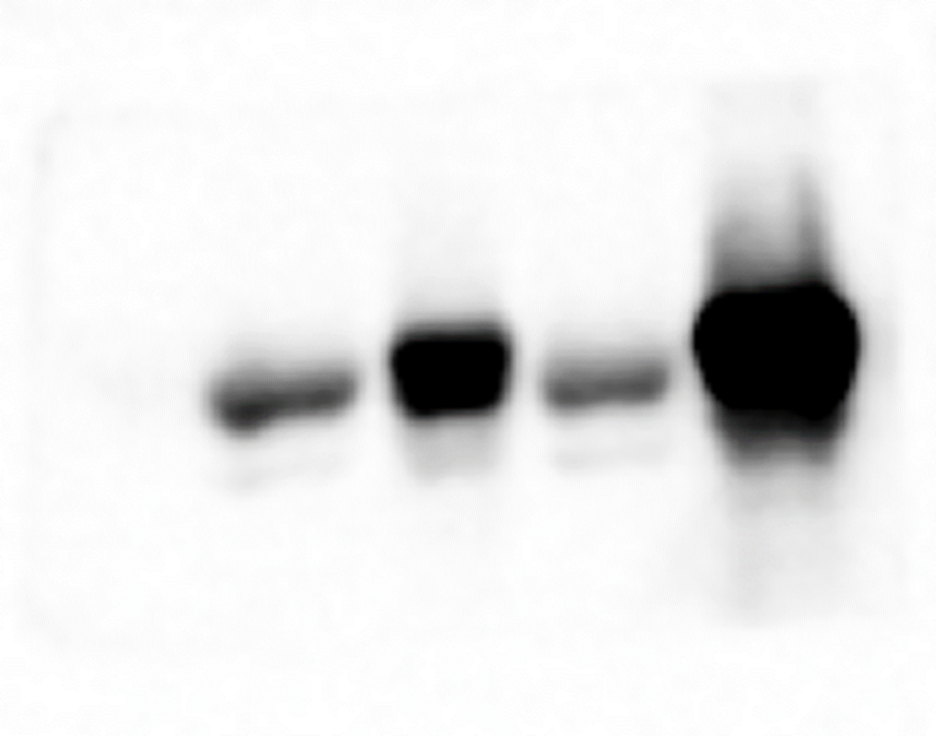

Supplement: Figure 7—source data 1. [file elife-84798-fig7-data1.zip › Figure 7-source data 3/Figure 7-source data 3.tif]

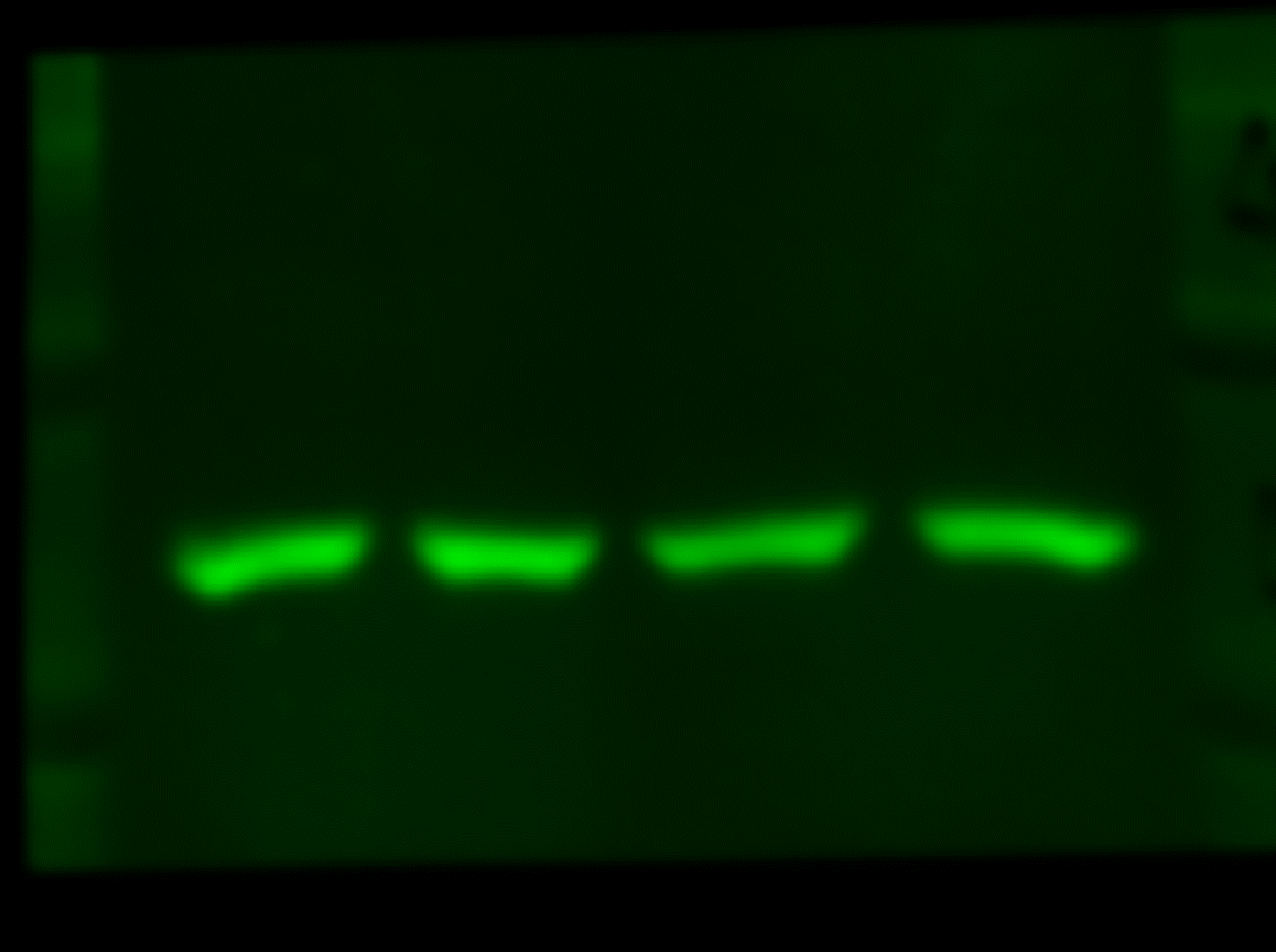

Supplement: Figure 7—source data 1. [file elife-84798-fig7-data1.zip › Figure 7-source data 4/Figure 7-source data 4.tif]

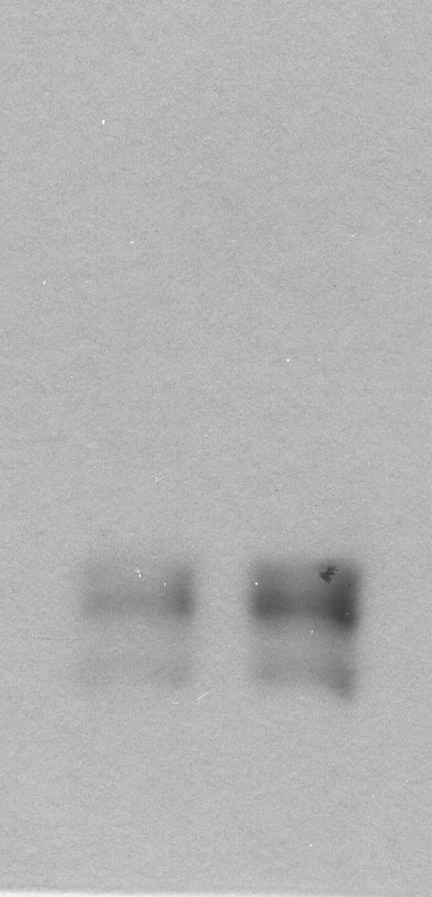

Supplement: Figure 7—source data 1. [file elife-84798-fig7-data1.zip › Figure 7-source data 5/Figure 7-source data 5.tif]

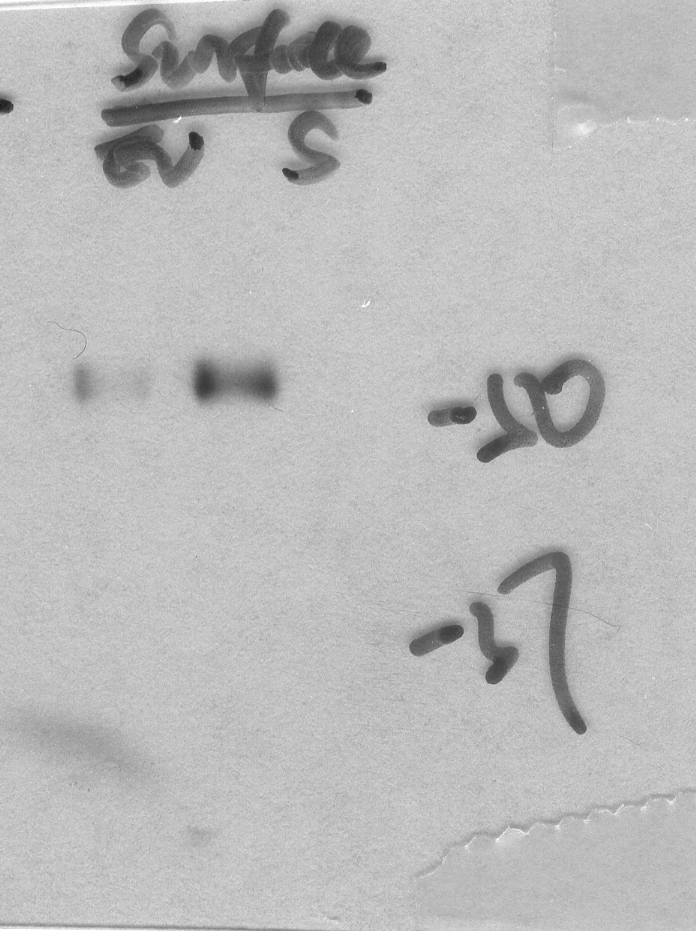

Supplement: Figure 7—source data 1. [file elife-84798-fig7-data1.zip › Figure 7-source data 6/Figure 7-source data 6.tif]

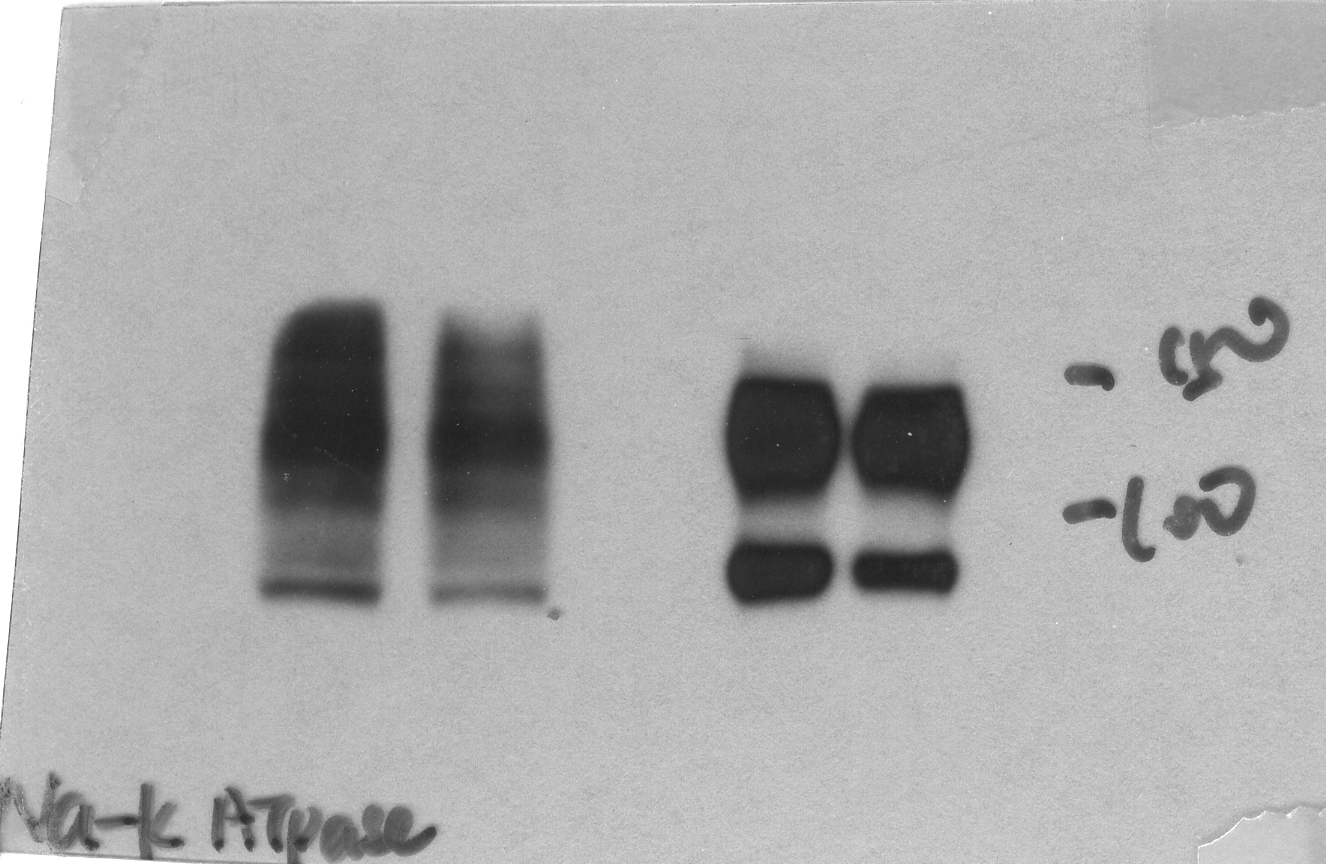

Supplement: Figure 7—source data 1. [file elife-84798-fig7-data1.zip › Figure 7-source data 7/Figure 7-source data 7.tif]

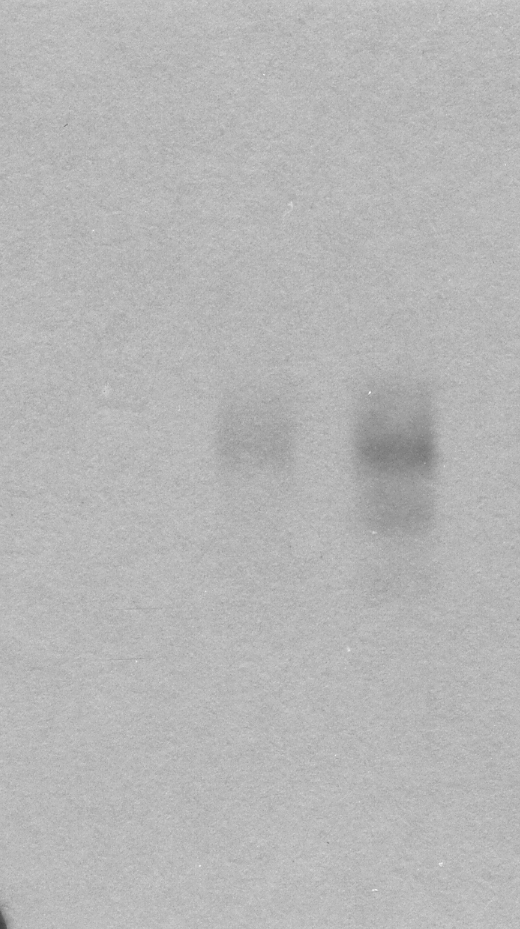

Supplement: Figure 7—source data 1. [file elife-84798-fig7-data1.zip › Figure 7-source data 8/Figure 7-source data 8.tif]

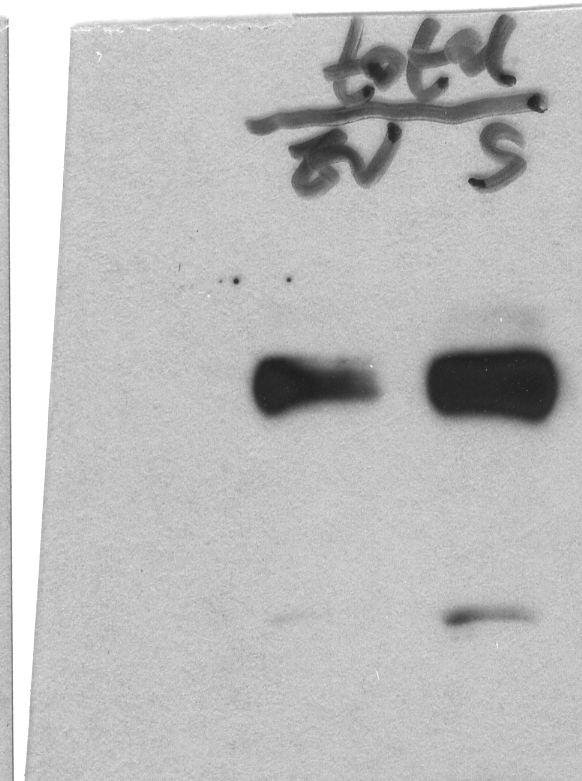

Supplement: Figure 7—source data 1. [file elife-84798-fig7-data1.zip › Figure 7-source data 9/Figure 7-source data 9.tif]

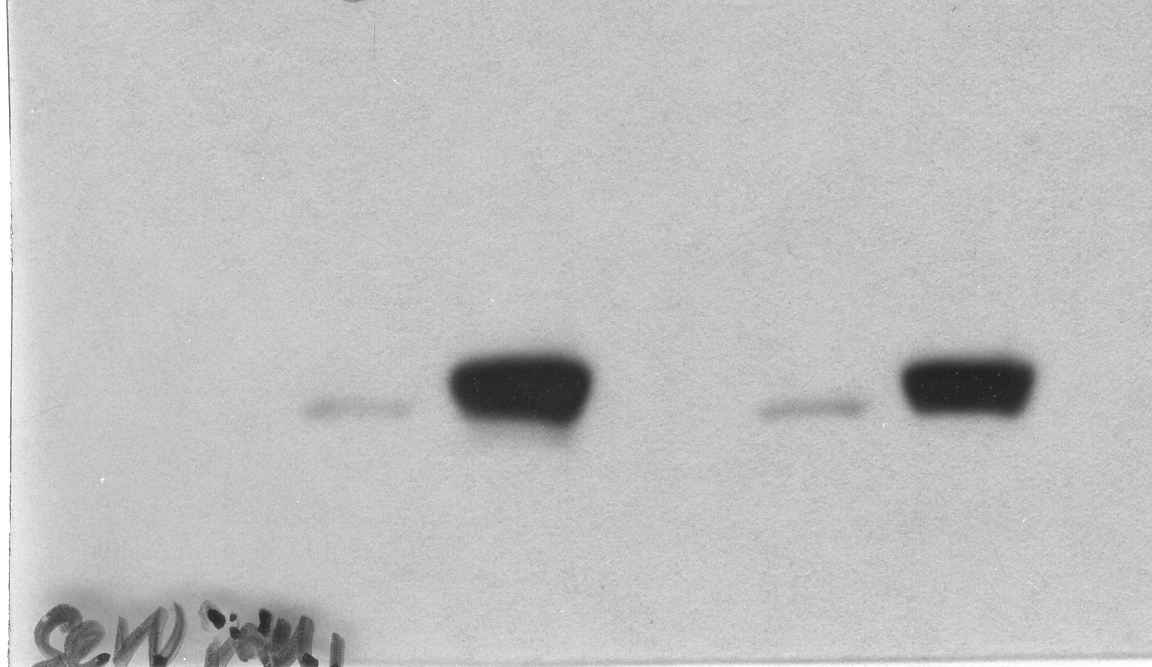

Supplement: Figure 7—source data 1. [file elife-84798-fig7-data1.zip › Figure 7-source data 10/Figure 7-source data 10.tif]

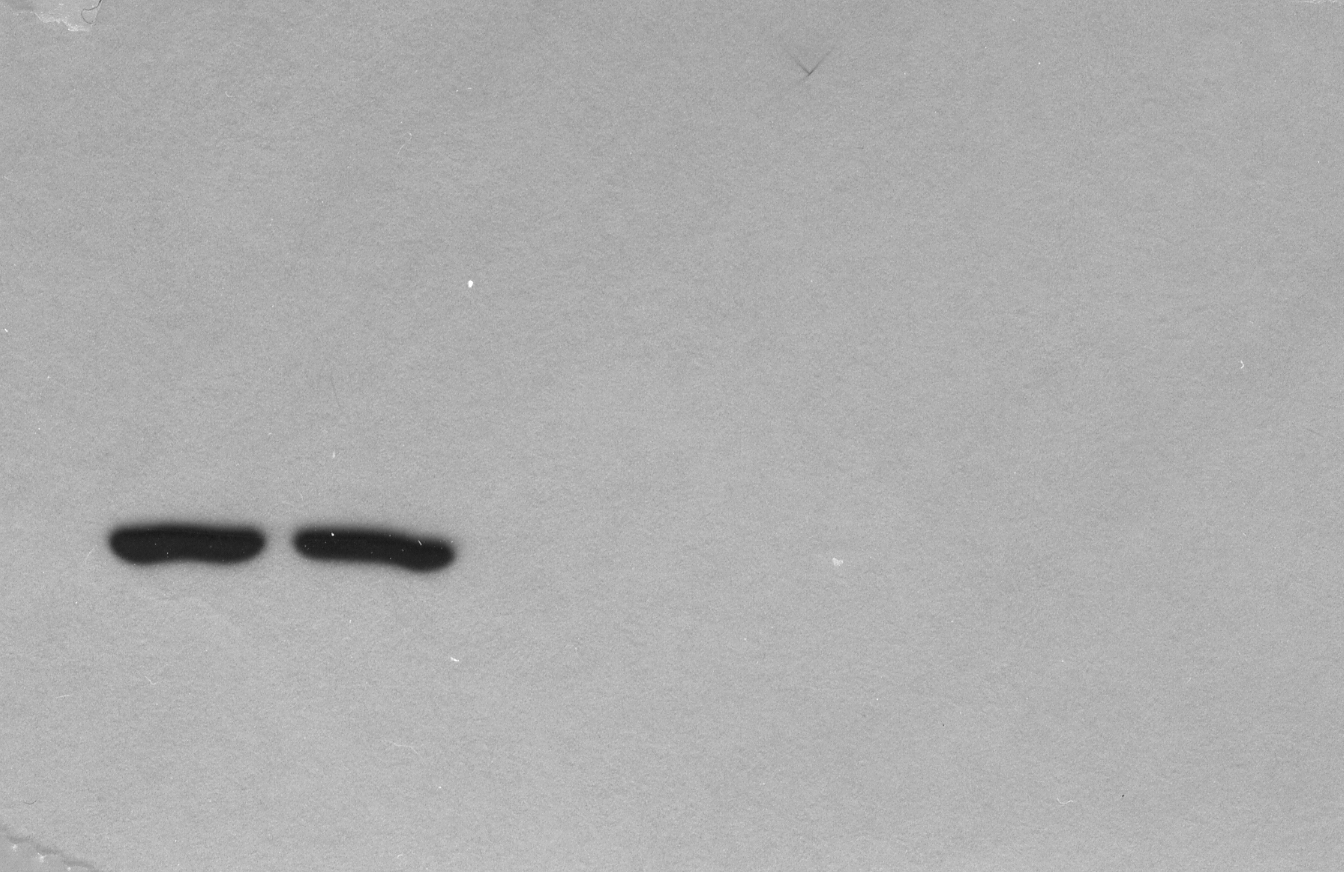

Supplement: Figure 7—source data 1. [file elife-84798-fig7-data1.zip › Figure 7-source data 11/Figure 7-source data 11.tif]

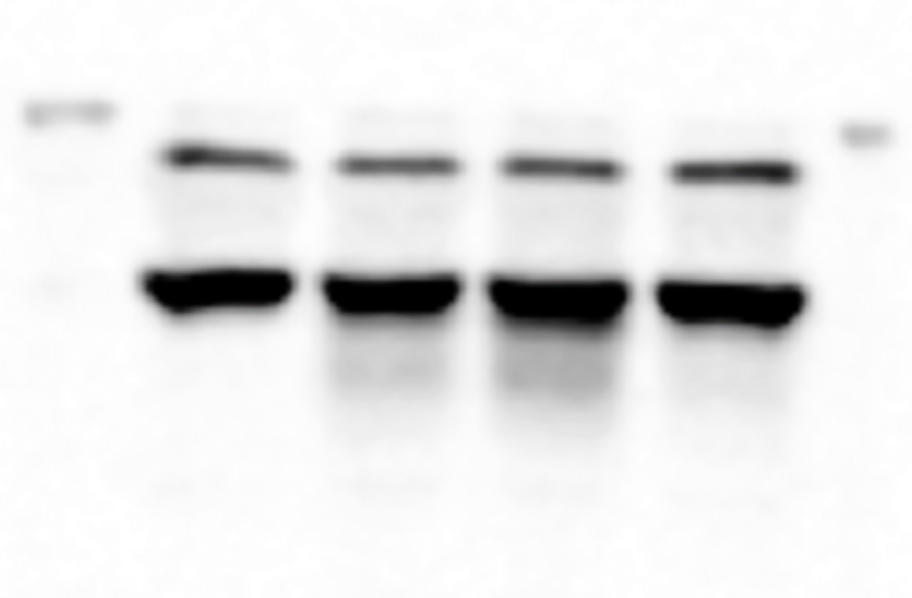

Supplement: Figure 7—figure supplement 1—source data 1. [file elife-84798-fig7-figsupp1-data1.zip › Figure 7-figure supplement 1-source data 1/Figure 7-figure supplement 1-source data 1.tif]

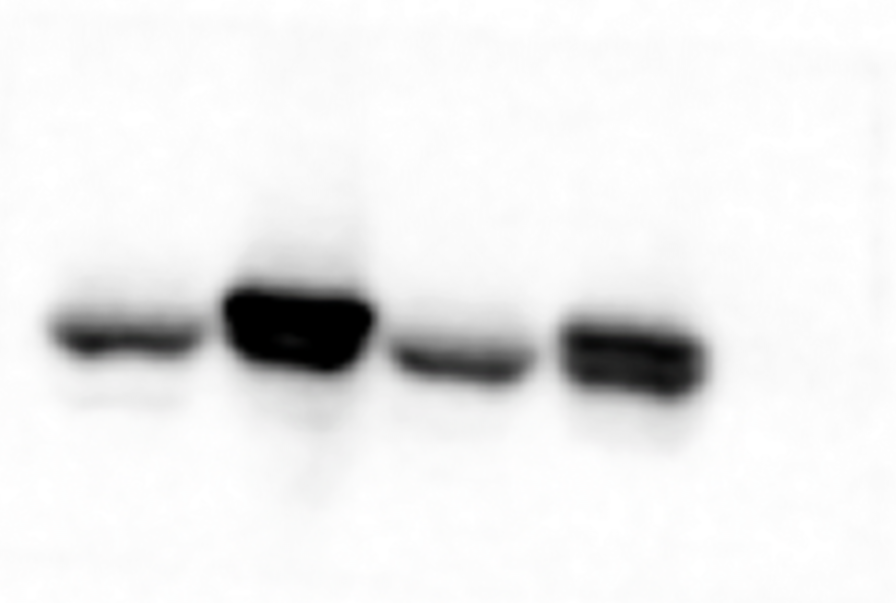

Supplement: Figure 7—figure supplement 1—source data 1. [file elife-84798-fig7-figsupp1-data1.zip › Figure 7-figure supplement 1-source data 2/Figure 7-figure supplement 1-source data 2.tif]

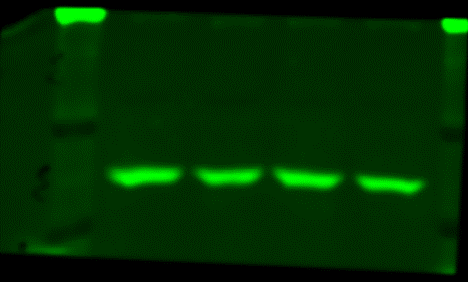

Supplement: Figure 7—figure supplement 1—source data 1. [file elife-84798-fig7-figsupp1-data1.zip › Figure 7-figure supplement 1-source data 3/Figure 7-figure supplement 1-source data 3.tif]

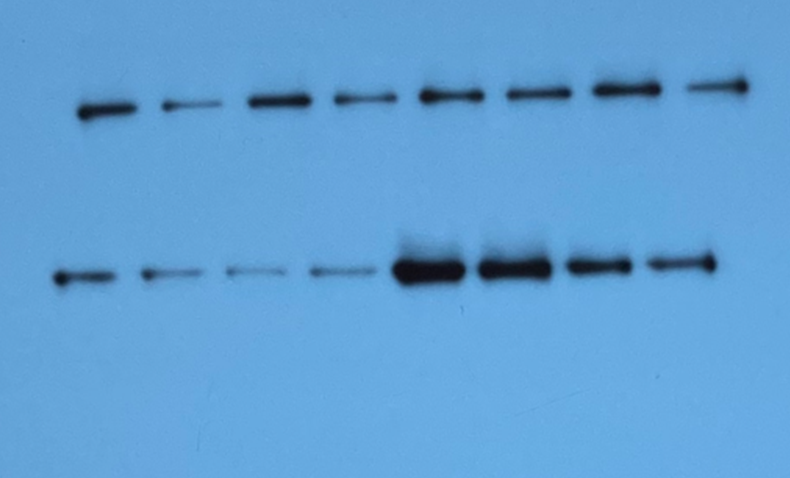

Supplement: Figure 7—figure supplement 2—source data 1. [file elife-84798-fig7-figsupp2-data1.zip › Figure 7-figure supplement 2-source data 1/Figure 7-figure supplement 2-source data 1.tif]

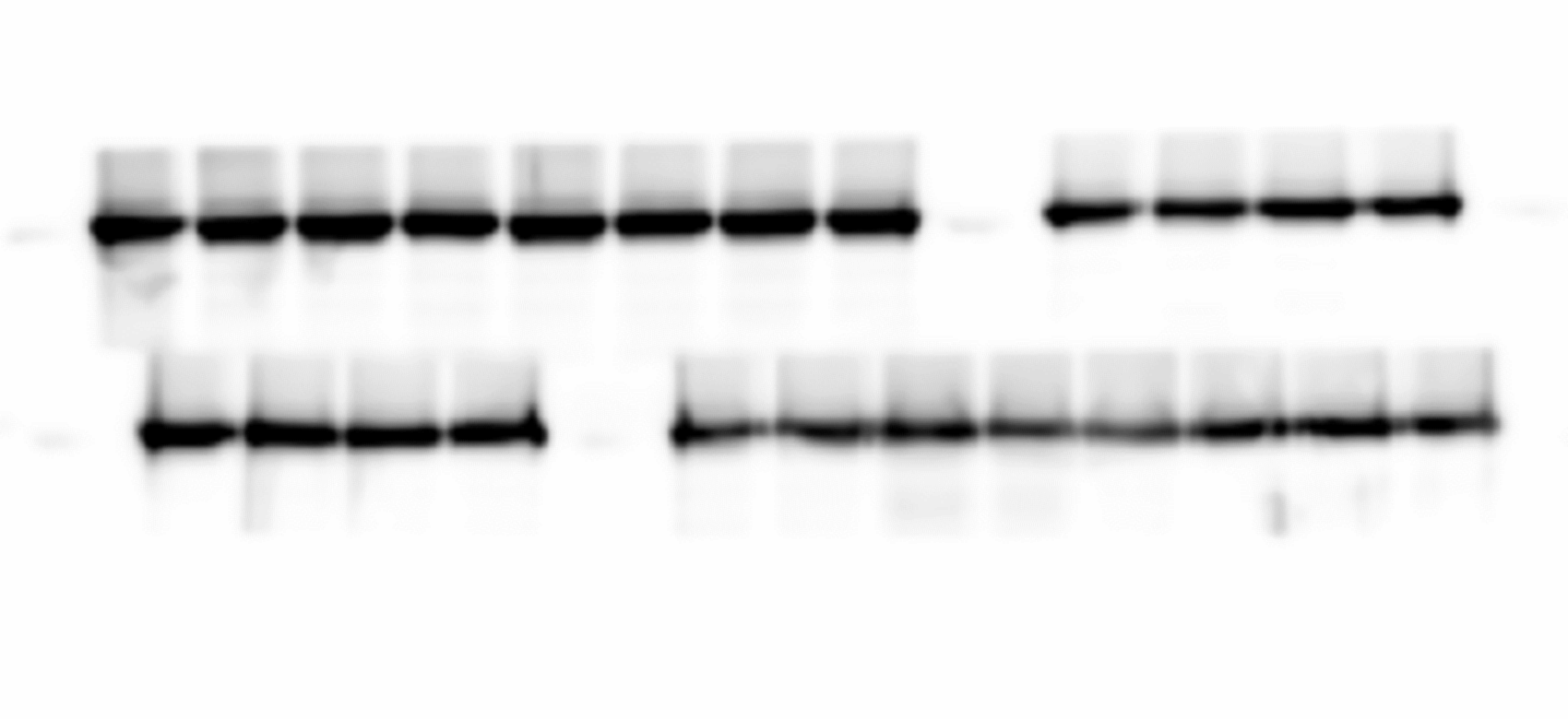

Supplement: Figure 7—figure supplement 2—source data 1. [file elife-84798-fig7-figsupp2-data1.zip › Figure 7-figure supplement 2-source data 2/Figure 7-figure supplement 2-source data 2.tif]

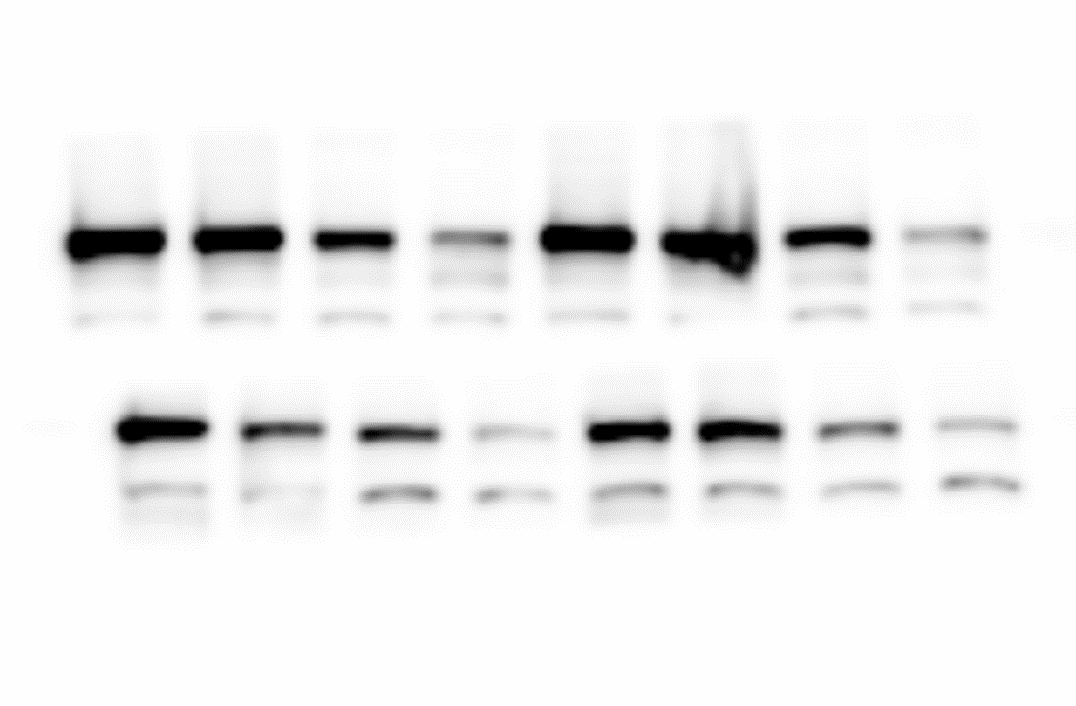

Supplement: Figure 7—figure supplement 2—source data 1. [file elife-84798-fig7-figsupp2-data1.zip › Figure 7-figure supplement 2-source data 3/Figure 7-figure supplement 2-source data 3.tif]

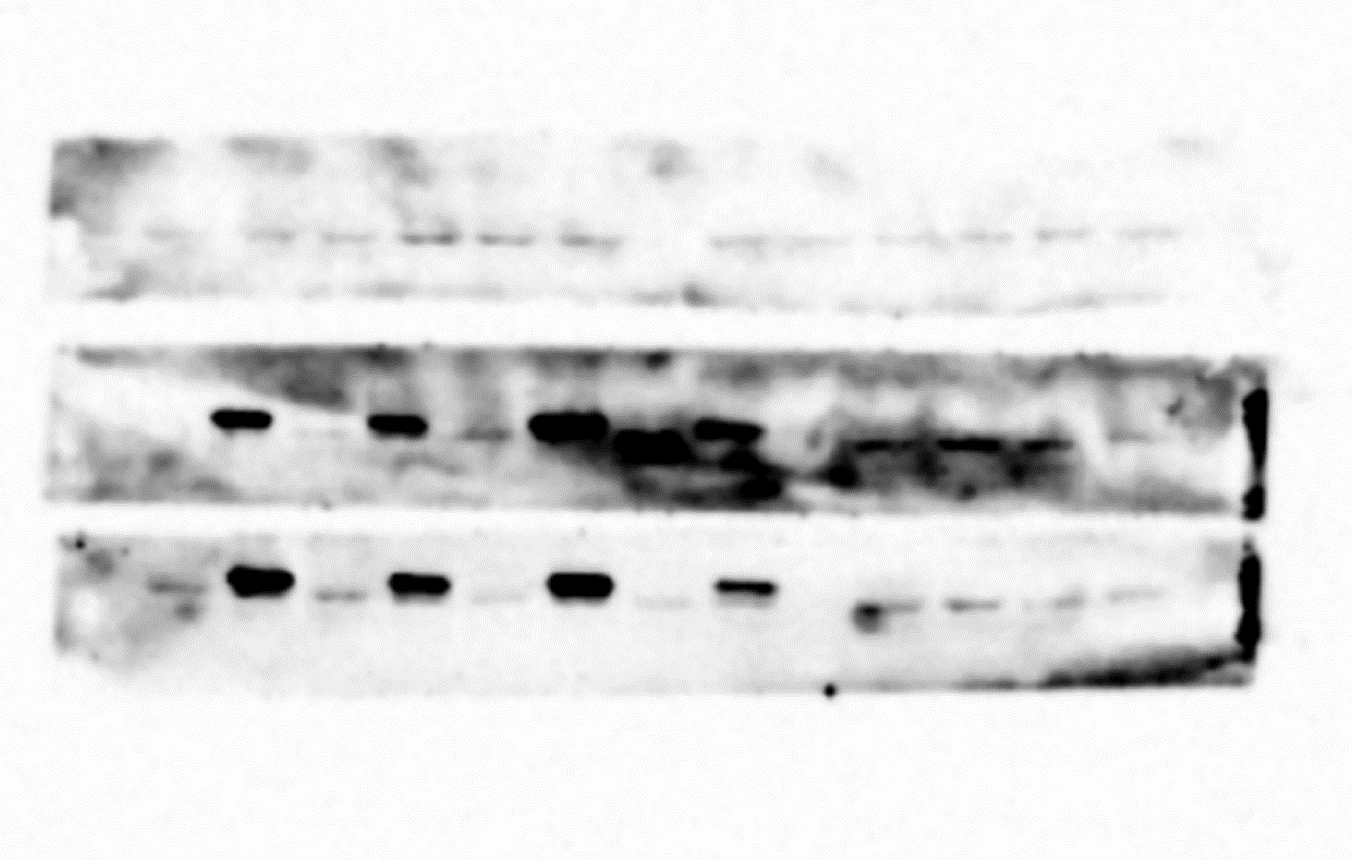

Supplement: Figure 7—figure supplement 2—source data 1. [file elife-84798-fig7-figsupp2-data1.zip › Figure 7-figure supplement 2-source data 4/Figure 7-figure supplement 2-source data 4.tif]

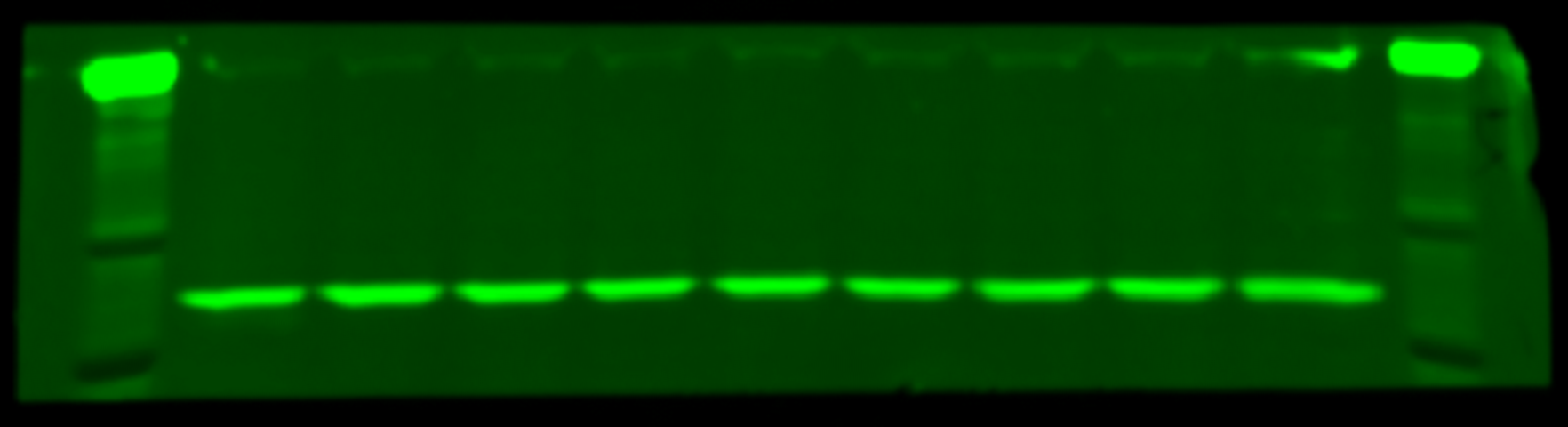

Supplement: Figure 7—figure supplement 2—source data 1. [file elife-84798-fig7-figsupp2-data1.zip › Figure 7-figure supplement 2-source data 5/Figure 7-figure supplement 2-source data 5.tif]

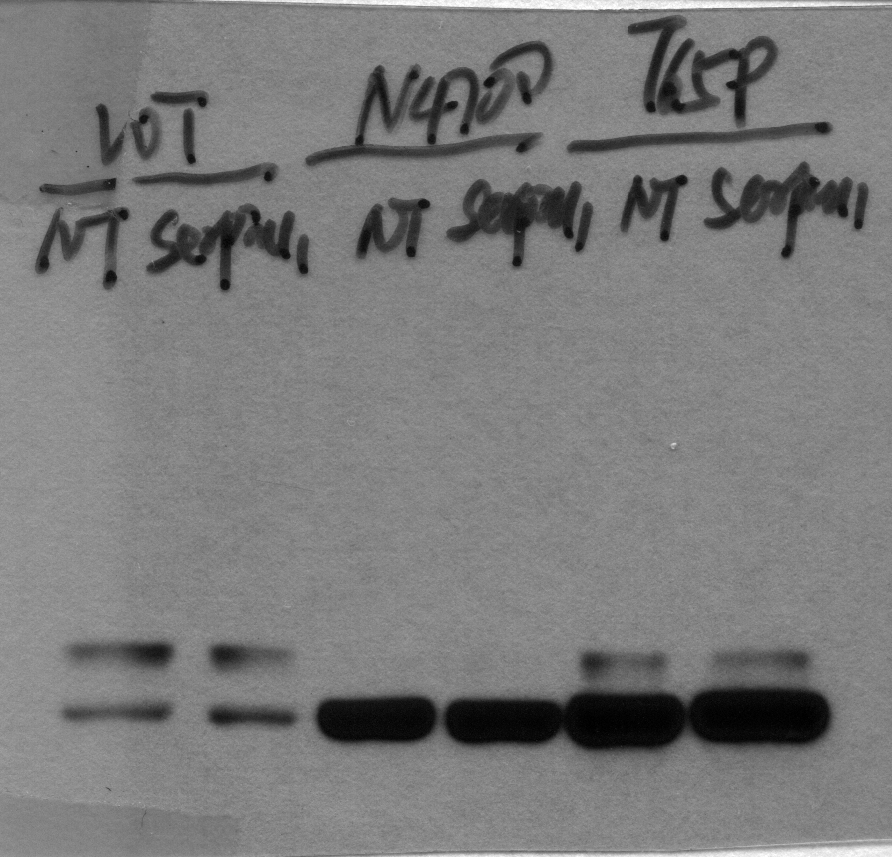

Supplement: Figure 7—figure supplement 2—source data 1. [file elife-84798-fig7-figsupp2-data1.zip › Figure 7-figure supplement 2-source data 6/Figure 7-figure supplement 2-source data 6.tif]

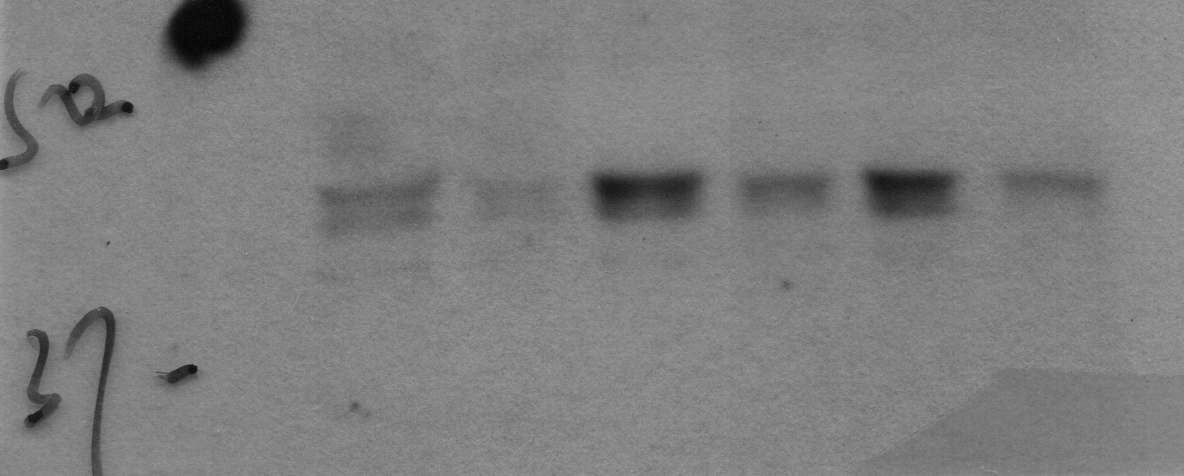

Supplement: Figure 7—figure supplement 2—source data 1. [file elife-84798-fig7-figsupp2-data1.zip › Figure 7-figure supplement 2-source data 7/Figure 7-figure supplement 2-source data 7.tif]

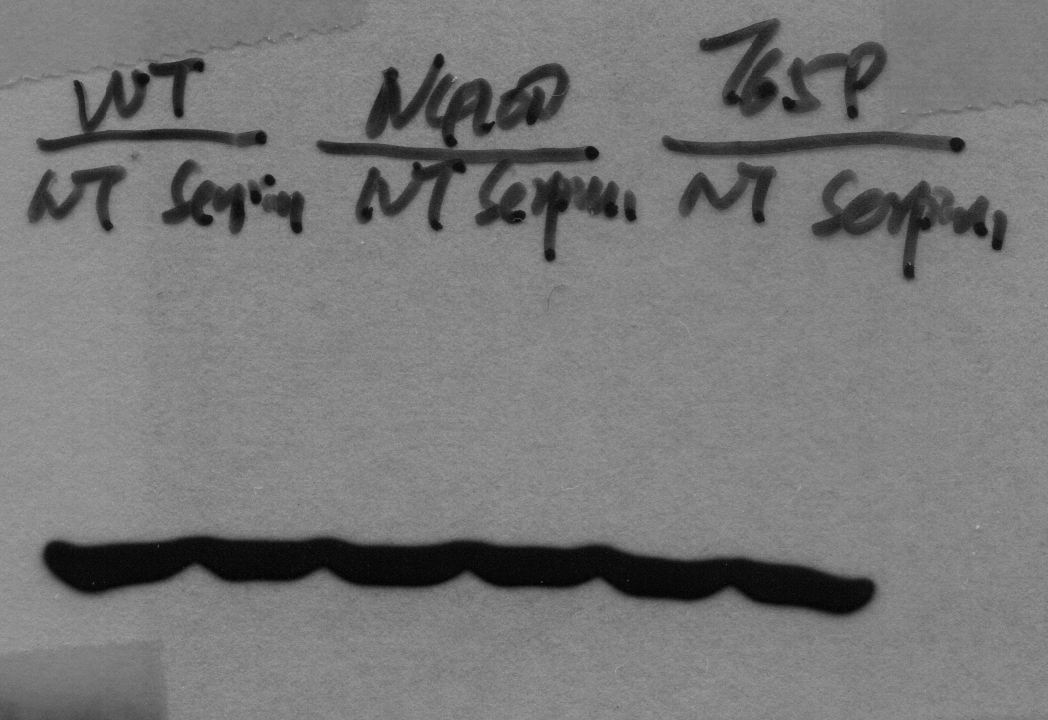

Supplement: Figure 7—figure supplement 2—source data 1. [file elife-84798-fig7-figsupp2-data1.zip › Figure 7-figure supplement 2-source data 8/Figure 7-figure supplement 2-source data 8.tif]

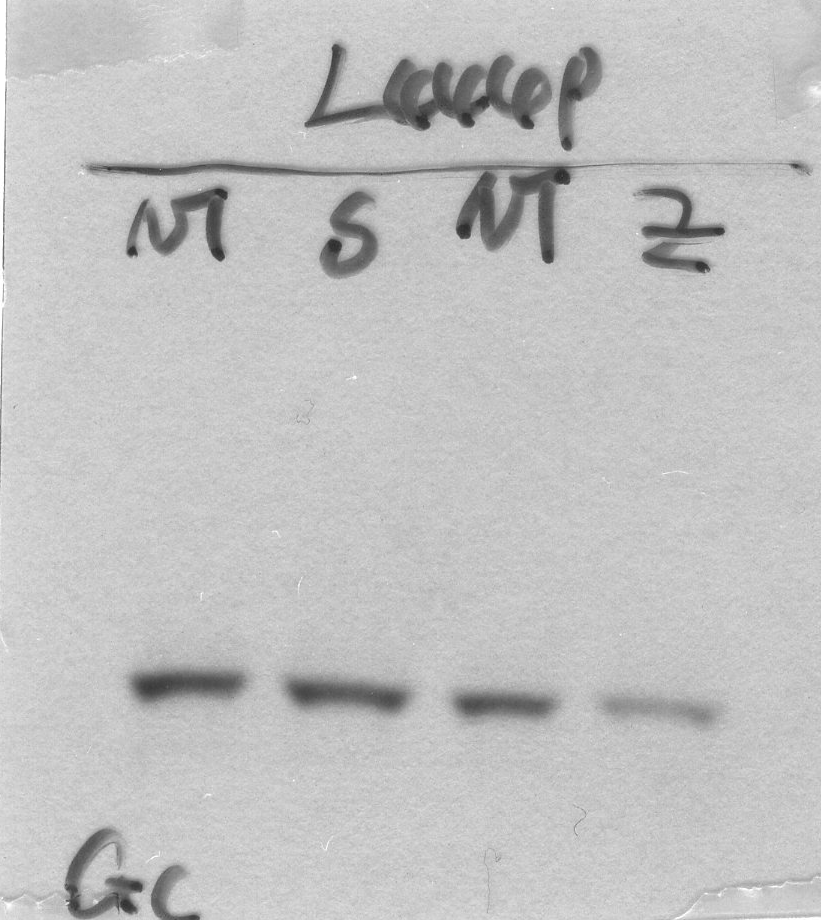

Supplement: Figure 7—figure supplement 2—source data 1. [file elife-84798-fig7-figsupp2-data1.zip › Figure 7-figure supplement 2-source data 9/Figure 7-figure supplement 2-source data 9.tif]

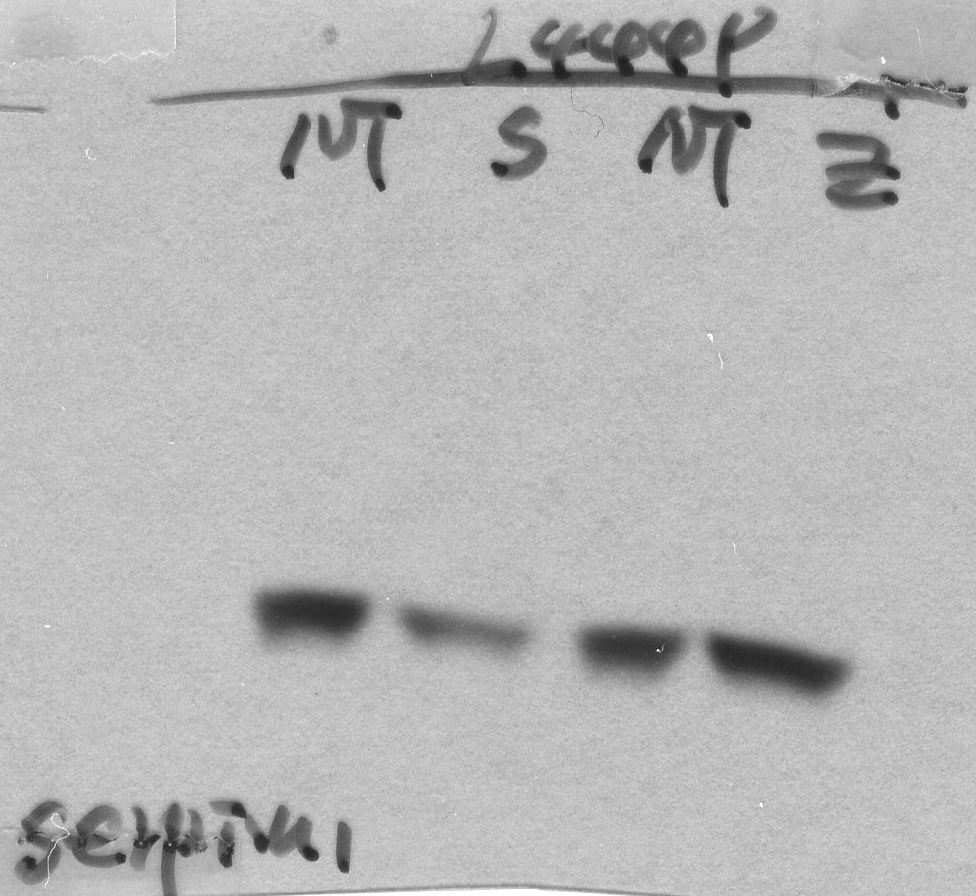

Supplement: Figure 7—figure supplement 2—source data 1. [file elife-84798-fig7-figsupp2-data1.zip › Figure 7-figure supplement 2-source data 10/Figure 7-figure supplement 2-source data 10.tif]

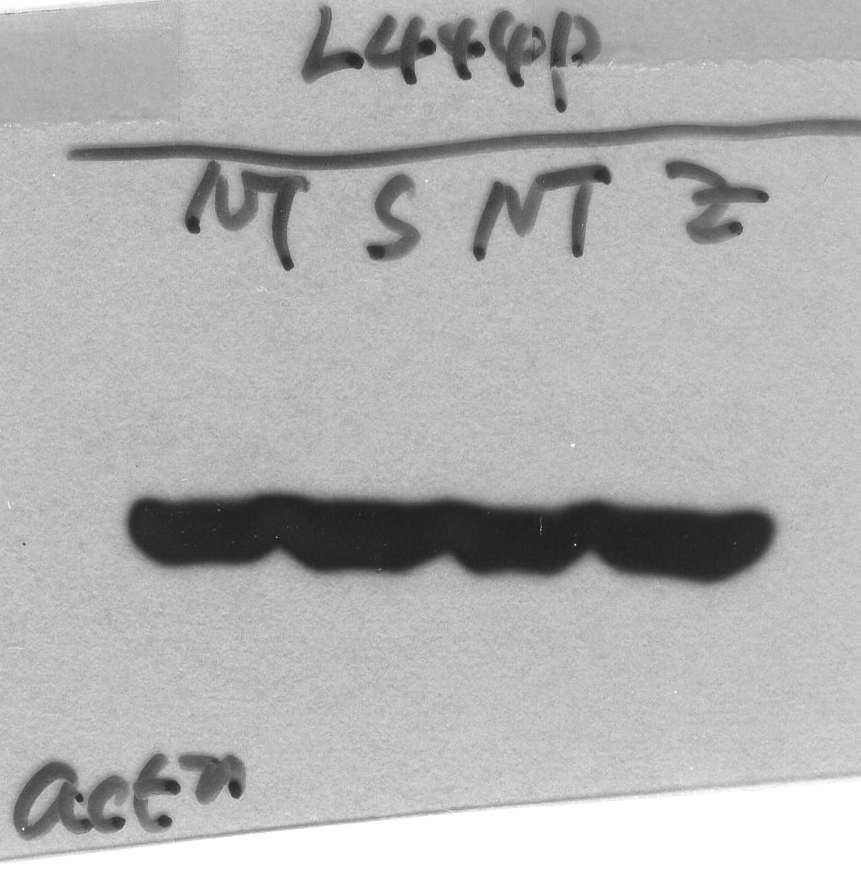

Supplement: Figure 7—figure supplement 2—source data 1. [file elife-84798-fig7-figsupp2-data1.zip › Figure 7-figure supplement 2-source data 11/Figure 7-figure supplement 2-source data 11.tif]
