# Supplementary material for: Hsp47 promotes biogenesis of multi-subunit neuroreceptors in the endoplasmic reticulum
Source: eLife. 2024 Jul 4;13:e84798. doi: 10.7554/eLife.84798 (PMC11257679; doi:10.7554/eLife.84798)
Supplement: Figure 6—source data 2. [file elife-84798-fig6-data2.zip › Figure 6-source data 49/Figure 6-source data 49.pdf]

Figure 6

Figure 6A
Left column
First row

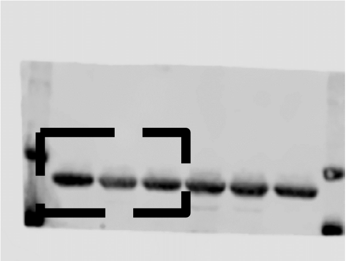

IB: Hsp47

Figure 6A
Left column
Second row

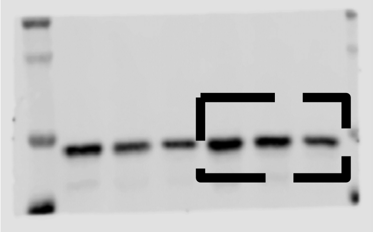

IB: α1

Figure 6A
Left column
Third row

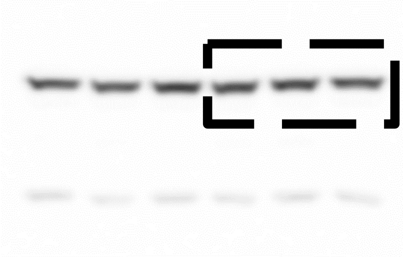

IB: BiP

Figure 6A
Left column
Fourth row

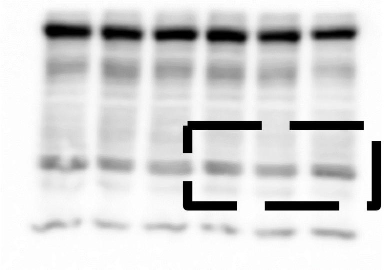

IB: ATF6-N

Figure 6A
Left column
Fifth row

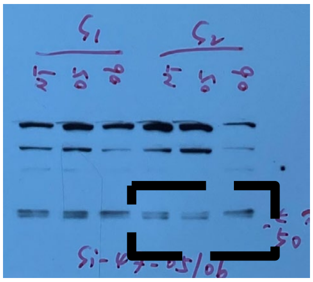

IB: XBP1s

Figure 6A
Left column
Sixth row

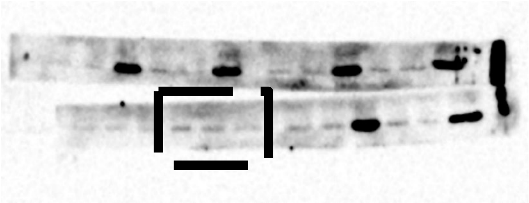

IB: CHOP

Figure 6A
Left column
Seventh row

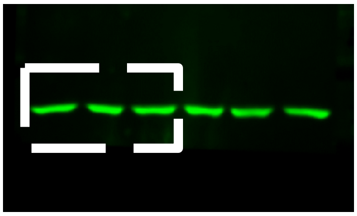

IB: β-actin

Figure 6-source data 1-7

Figure 6

Figure 6A
Right column
First row

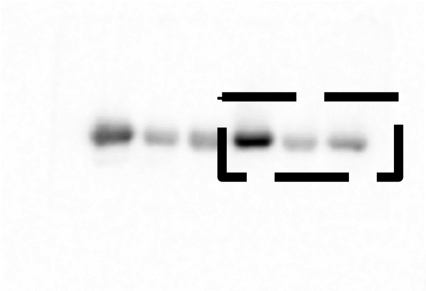

IB: Hsp47

Figure 6A
Right column
Second row

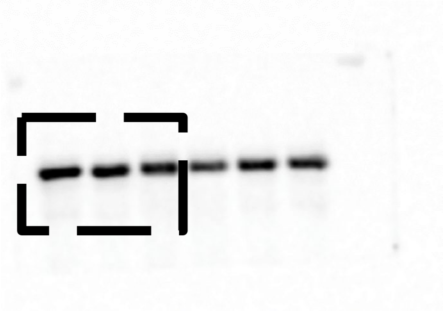

IB: $\alpha 1$

Figure 6A
Right column
Third row

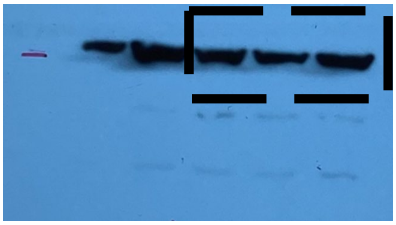

IB: BiP

Figure 6A
Right column
Fourth row

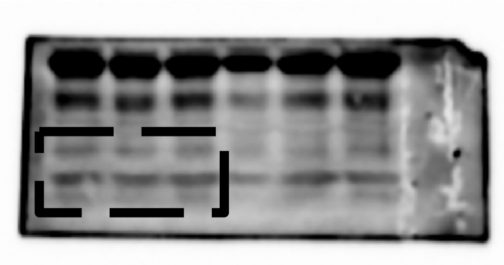

IB: ATF6-N

Figure 6A
Right column
Fifth row

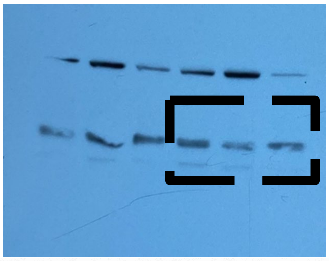

IB: XBP1s

Figure 6A
Right column
Sixth row

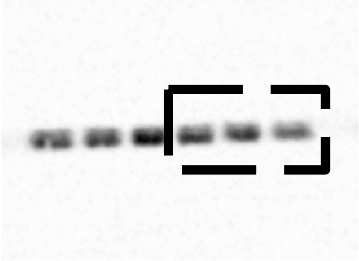

IB: CHOP

Figure 6A
Right column
Seventh row

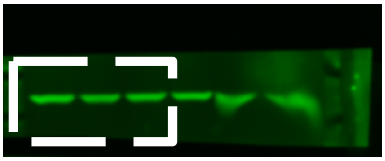

IB: β-actin

Figure 6-source data 8-14

Figure 6

Figure 6B
Left column
First row

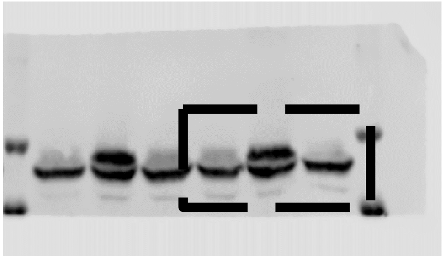

IB: Hsp47

Figure 6B
Left column
Second row

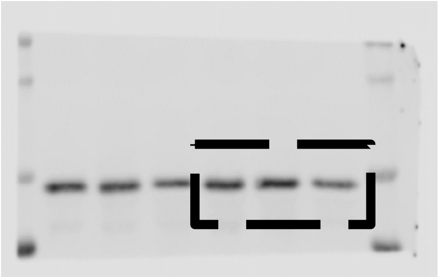

IB: α1

Figure 6B
Left column
Third row

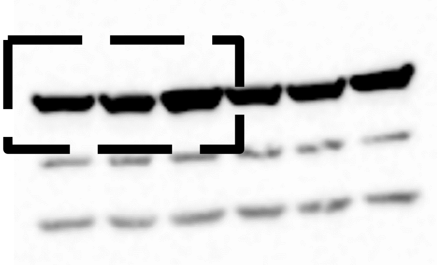

IB: BiP

Figure 6B
Left column
Fourth row

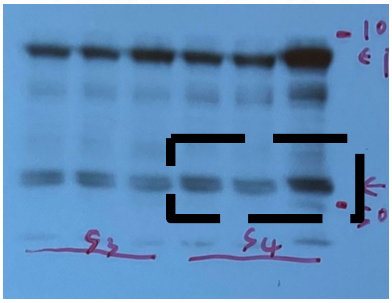

IB: ATF6-N

Figure 6B
Left column
Fifth row

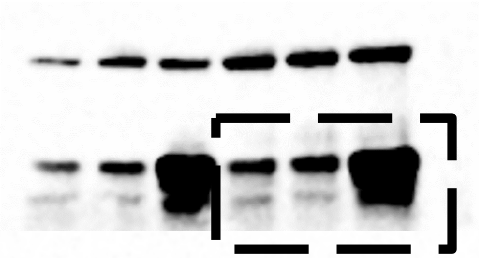

IB: XBP1s

Figure 6B
Left column
Sixth row

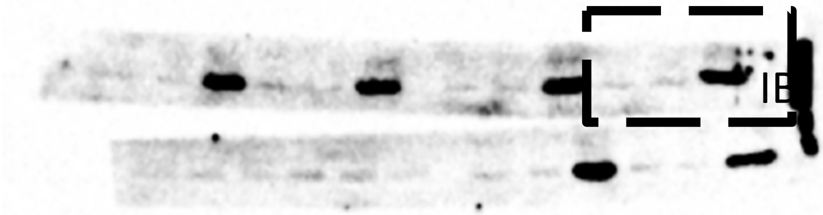

IB: CHOP

Figure 6B
Left column
Seventh row

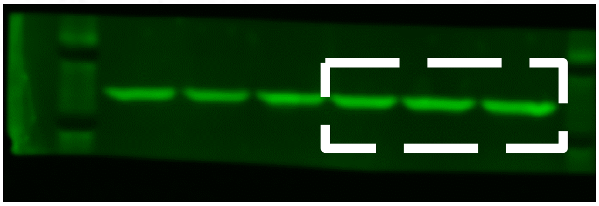

IB: β-actin

Figure 6-source data 15-21

Figure 6

Figure 6B
Right column
First row

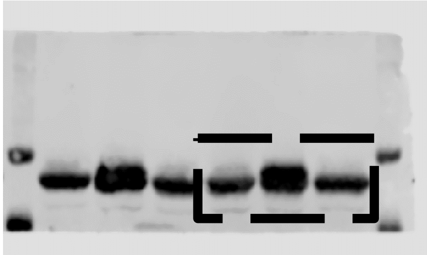

IB: Hsp47

Figure 6B
Right column
Second row

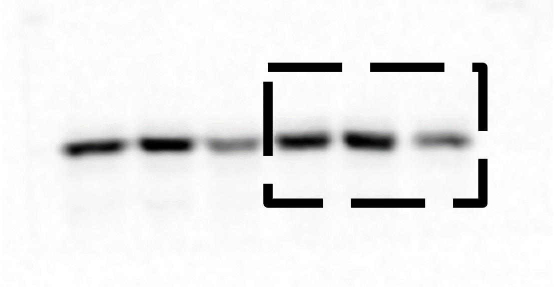

IB: α1

Figure 6B
Right column
Third row

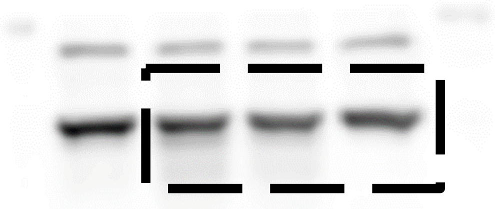

IB: BiP

Figure 6B
Right column
Fourth row

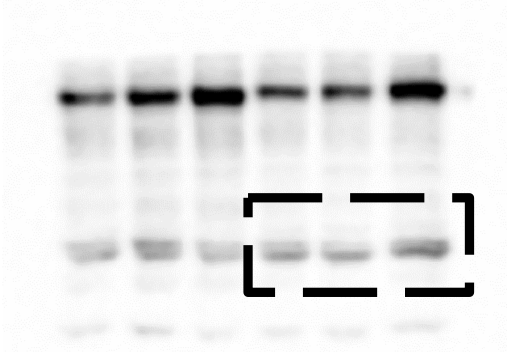

IB: ATF6-N

Figure 6B
Right column
Fifth row

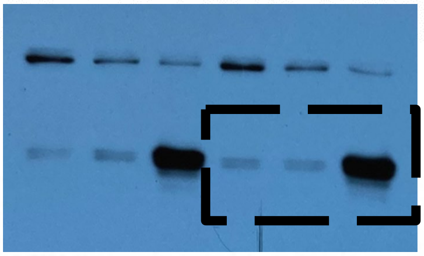

IB: XBP1s

Figure 6B
Right column
Sixth row

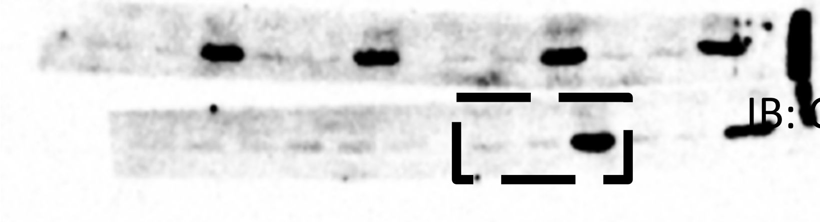

IB: CHOP

Figure 6B
Right column
Seventh row

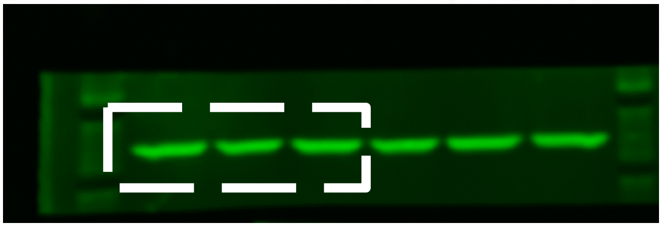

IB: β-actin

Figure 6-source data 22-28

Figure 6

IB: Hsp47

Figure 6C
First row

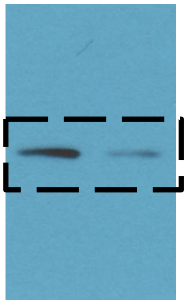

IB: α1

Figure 6C
Second row

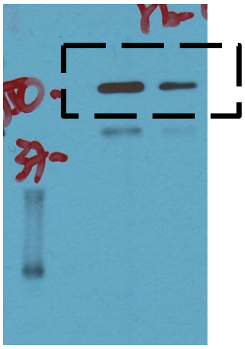

IB: β2/3

Figure 6C
Third row

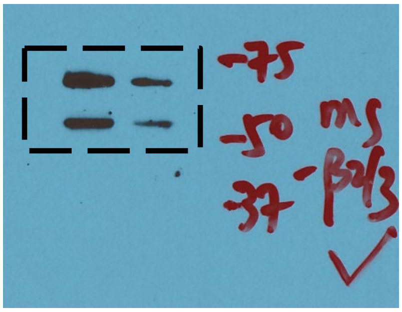

IB: γ2

Figure 6C
Fourth row

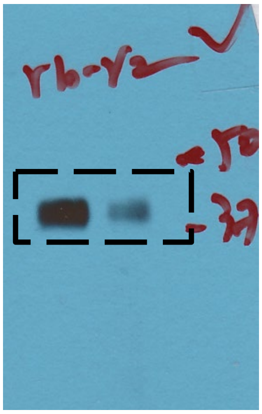

IB: BiP

Figure 6C
Fifth row

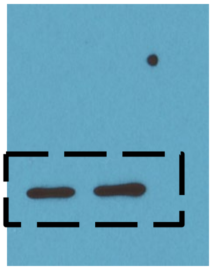

IB: ATF6

Figure 6C
Sixth row

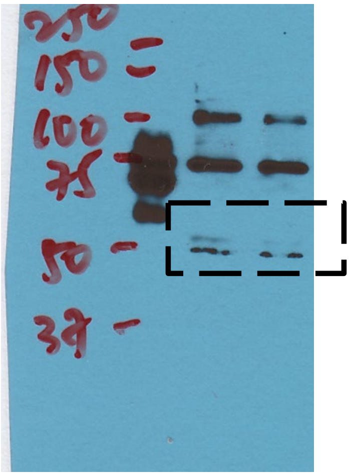

IB: XBP1

Figure 6C
Seventh row

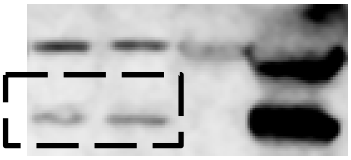

IB: β-actin

Figure 6C
Eighth row

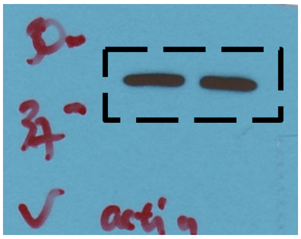

Figure 6-source data 29-36

Figure 6

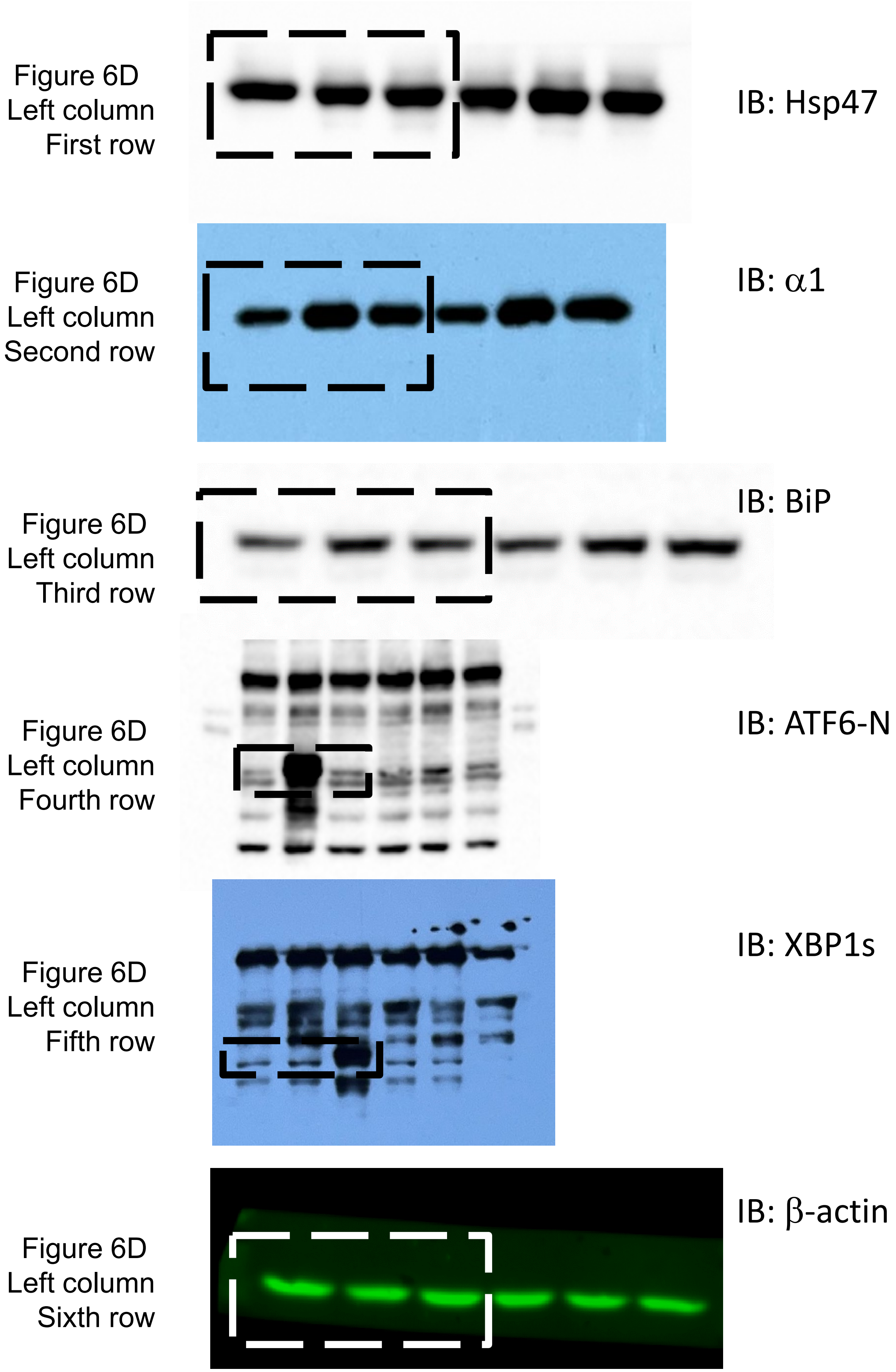

Figure 6-source data 37-42

Figure 6

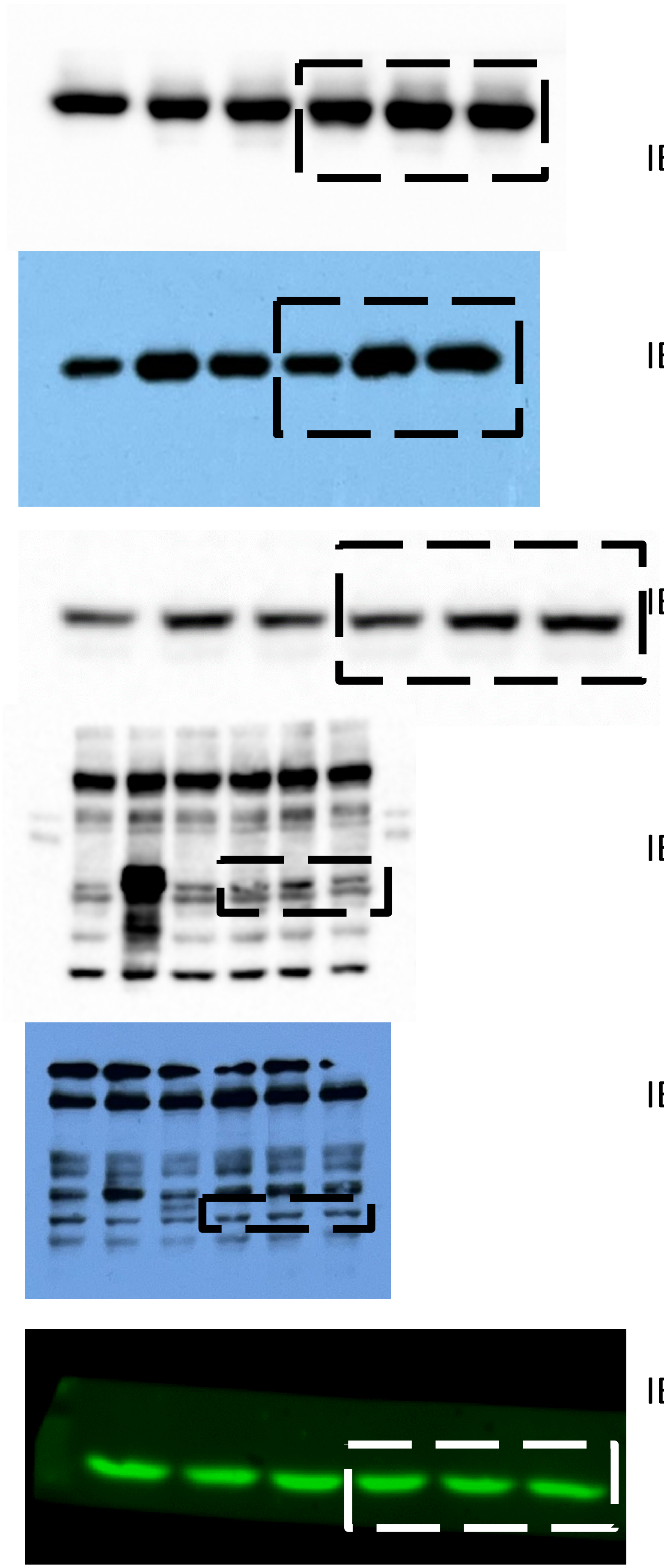

Figure 6-source data 43-48
