# Supplementary material for: Hsp47 promotes biogenesis of multi-subunit neuroreceptors in the endoplasmic reticulum
Source: eLife. 2024 Jul 4;13:e84798. doi: 10.7554/eLife.84798 (PMC11257679; doi:10.7554/eLife.84798)
Supplement: Figure 7—source data 2. [file elife-84798-fig7-data2.zip › Figure 7-source data 12/Figure 7-source data 12.pdf]

Figure 7

Figure 7A
First row

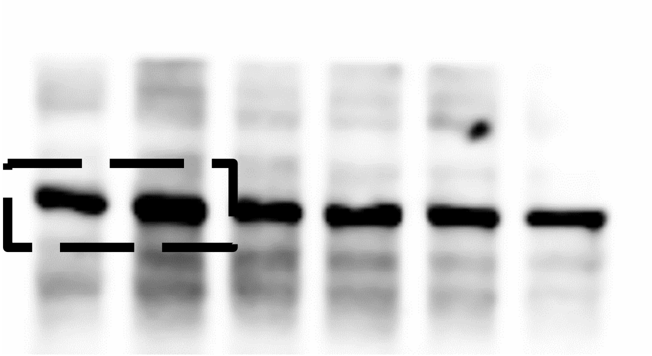

IB: α4

Figure 7A
Second row

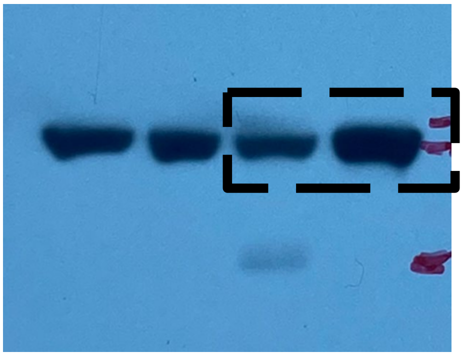

IB: β2

Figure 7A
Third row

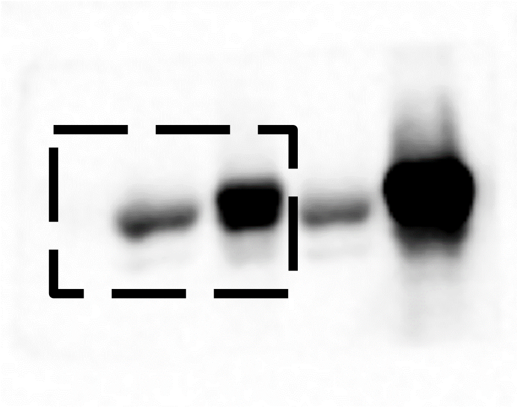

IB: Hsp47

Figure 7A
Fourth row

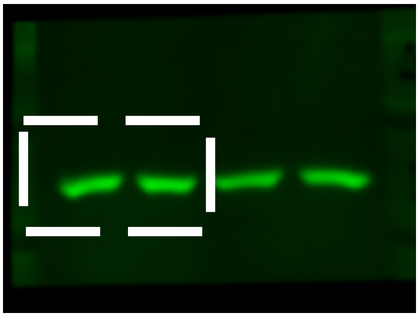

IB: β-actin

Figure 7-source data 1-4

Figure 7

Figure 7D
First row
IB: 5-HT_3A

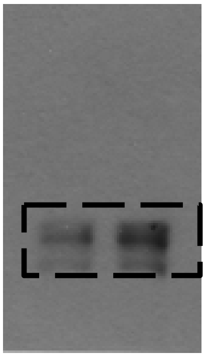

Figure 7D
Second row
IB: Flag

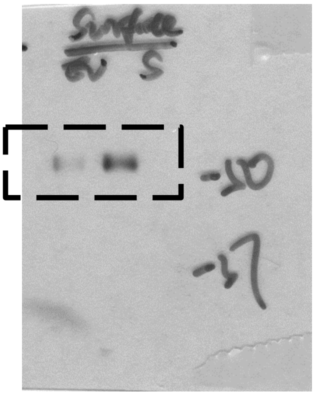

Figure 7D
Third row
IB: ATPase

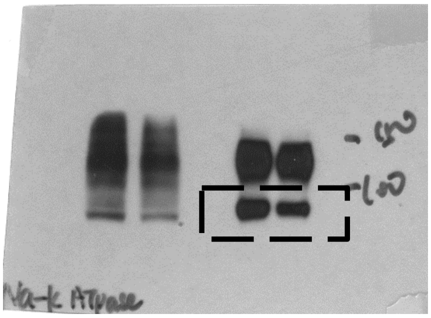

Figure 7D
Fourth row
IB: 5-HT_3A

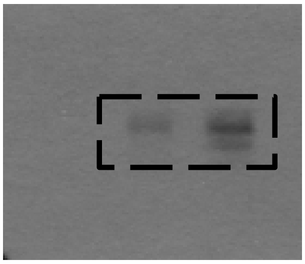

Figure 7D
Fifth row
IB: Flag

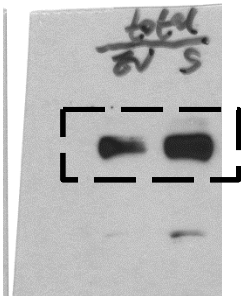

Figure 7D
Sixth row
IB: Hsp47

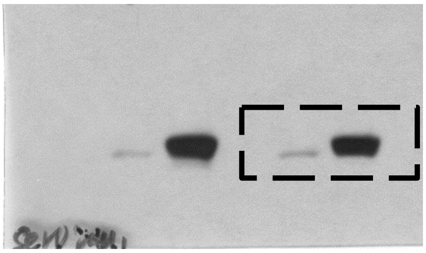

Figure 7D
Seventh row
IB: β-actin

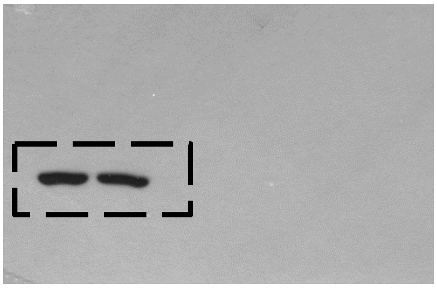

Figure 7-source data 5-11
