# Supplementary material for: Hsp47 promotes biogenesis of multi-subunit neuroreceptors in the endoplasmic reticulum
Source: eLife. 2024 Jul 4;13:e84798. doi: 10.7554/eLife.84798 (PMC11257679; doi:10.7554/eLife.84798)
Supplement: Figure 7—figure supplement 1—source data 2. [file elife-84798-fig7-figsupp1-data2.zip › Figure 7-figure supplement 1-source data 4/Figure 7-figure supplement 1-source data 4.pdf]

Figure 7-figure supplement 1

Figure 7-figure supplement 1B
Top panel

IB: α7

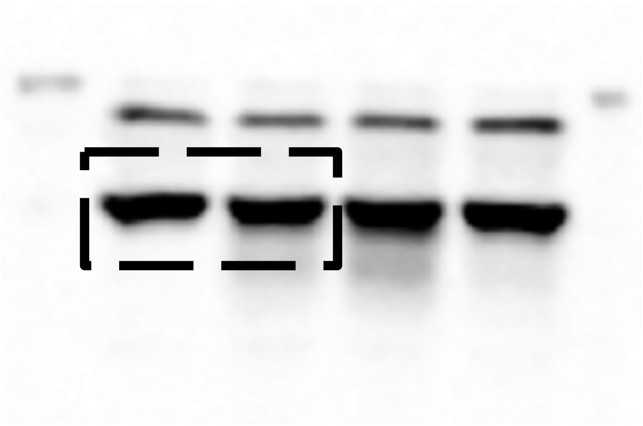

Figure 7-figure supplement 1B
Middle panel

IB: Hsp47

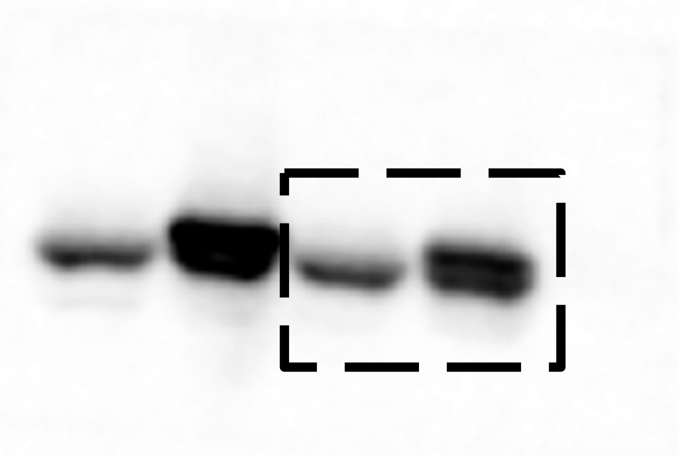

Figure 7-figure supplement 1B
Bottom panel

IB: β-actin

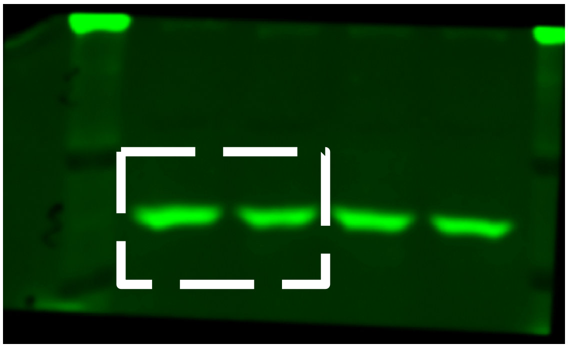

Figure 7-figure supplement 1-source data 1-3
