# Supplementary material for: Hsp47 promotes biogenesis of multi-subunit neuroreceptors in the endoplasmic reticulum
Source: eLife. 2024 Jul 4;13:e84798. doi: 10.7554/eLife.84798 (PMC11257679; doi:10.7554/eLife.84798)
Supplement: Figure 7—figure supplement 2—source data 2. [file elife-84798-fig7-figsupp2-data2.zip › Figure 7-figure supplement 2-source data 12/Figure 7-figure supplement 2-source data 12.pdf]

Figure 7-figure supplement 2

Figure 7-figure supplement 2A
First row
IB: GluN2A

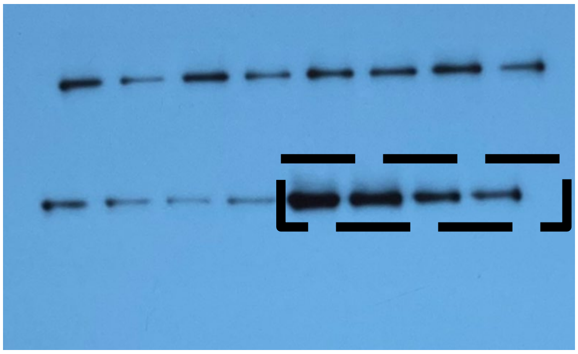

Figure 7-figure supplement 2A
Second row
IB: ATPase

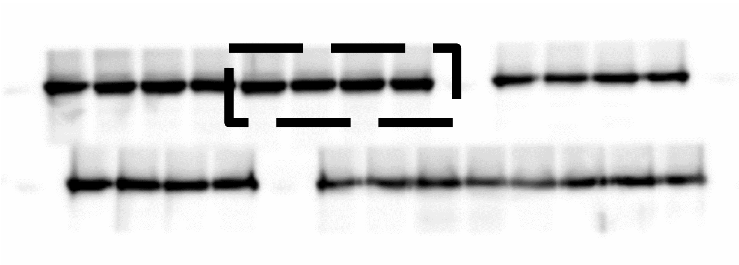

Figure 7-figure supplement 2A
Third row
IB: GluN2A

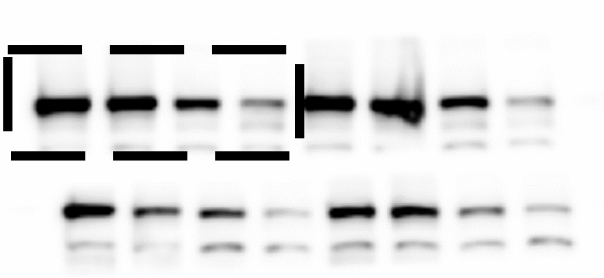

Figure 7-figure supplement 2A
Fourth row
IB: Hsp47

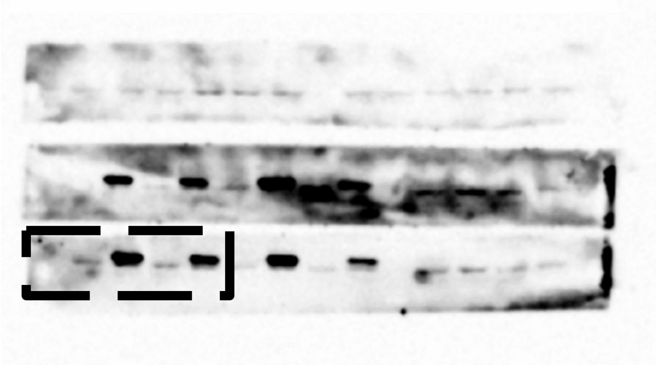

Figure 7-figure supplement 2A
Fifth row
IB: β-actin

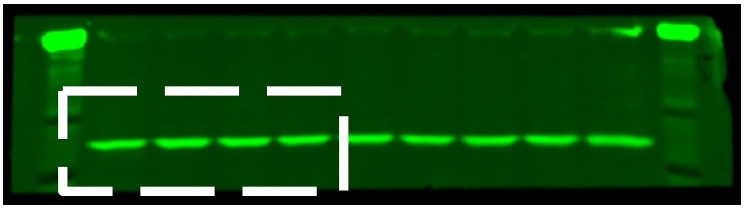

Figure 7-figure supplement 2-source data 1-5

Figure 7-figure supplement 2

Figure 7-figure supplement 2B
Top panel

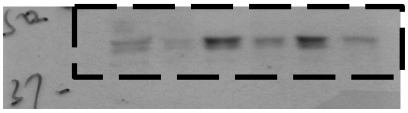

IB: Hsp47

Figure 7-figure supplement 2B
Middle panel

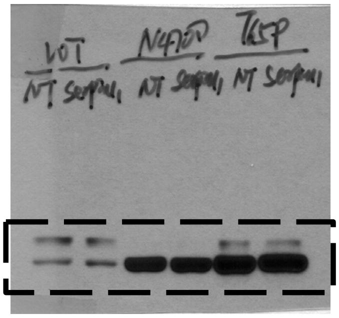

IB: Flag

Figure 7-figure supplement 2B
Bottom panel

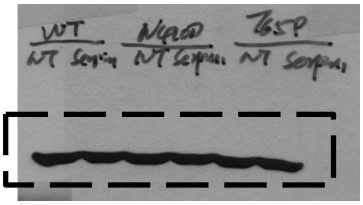

IB: β-actin

Figure 7-figure supplement 2C
Top panel

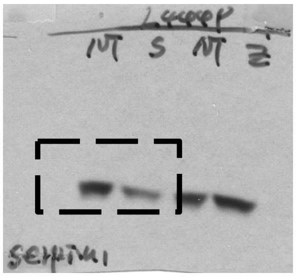

IB: Hsp47

Figure 7-figure supplement 2C
Middle panel

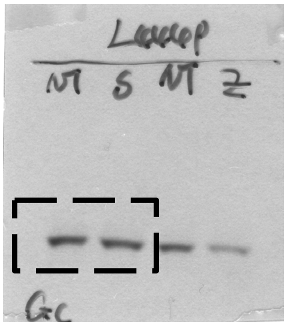

IB: GC

Figure 7-figure supplement 2C
Bottom panel

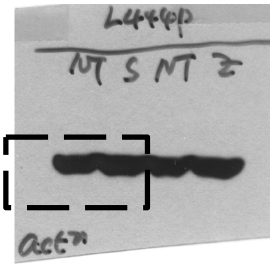

IB: β-actin

Figure 7-figure supplement 2-source data 6-11
